# Methyl 2-Halo-4-Substituted-5-Sulfamoyl-Benzoates as High Affinity and Selective Inhibitors of Carbonic Anhydrase IX

**DOI:** 10.3390/ijms23010130

**Published:** 2021-12-23

**Authors:** Audrius Zakšauskas, Edita Čapkauskaitė, Vaida Paketurytė-Latvė, Alexey Smirnov, Janis Leitans, Andris Kazaks, Elviss Dvinskis, Laimonas Stančaitis, Aurelija Mickevičiūtė, Jelena Jachno, Linas Jezepčikas, Vaida Linkuvienė, Andrius Sakalauskas, Elena Manakova, Saulius Gražulis, Jurgita Matulienė, Kaspars Tars, Daumantas Matulis

**Affiliations:** 1 Department of Biothermodynamics and Drug Design, Institute of Biotechnology, Life Sciences Center, Vilnius University, Saulėtekio al. 7, LT-10257 Vilnius, Lithuania; audrius.zaksauskas@bti.vu.lt (A.Z.); edita.capkauskaite@bti.vu.lt (E.Č.); vaida.paketuryte@gmc.vu.lt (V.P.-L.); alexeyus1@gmail.com (A.S.); laimonas.stancaitis@gmc.vu.lt (L.S.); aurelija.mickeviciute@bti.vu.lt (A.M.); jelena.jachno@bti.vu.lt (J.J.); linasje@gmail.com (L.J.); linkuviene.vaida@gmail.com (V.L.); andrius.sakalauskas@gmc.vu.lt (A.S.); jurgita.matuliene@bti.vu.lt (J.M.); 2 Latvian Biomedical Research and Study Centre, Ratsupites 1 k-1, LV-1067 Riga, Latvia; janis.leitans@biomed.lu.lv (J.L.); andris@biomed.lu.lv (A.K.); elviss.dvinskis@protonmail.com (E.D.); kaspars@biomed.lu.lv (K.T.); 3 Department of Protein—DNA Interactions, Institute of Biotechnology, Life Sciences Center, Vilnius University, Saulėtekio al. 7, LT-10257 Vilnius, Lithuania; elena.manakova@bti.vu.lt (E.M.); saulius.grazulis@bti.vu.lt (S.G.)

**Keywords:** methyl 5-sulfamoyl-benzoate, human carbonic anhydrase, *intrinsic* binding thermodynamics, enzyme inhibitor, X-ray crystallography, fluorescent thermal shift assay

## Abstract

Among the twelve catalytically active carbonic anhydrase isozymes present in the human body, the CAIX is highly overexpressed in various solid tumors. The enzyme acidifies the tumor microenvironment enabling invasion and metastatic processes. Therefore, many attempts have been made to design chemical compounds that would exhibit high affinity and selective binding to CAIX over the remaining eleven catalytically active CA isozymes to limit undesired side effects. It has been postulated that such drugs may have anticancer properties and could be used in tumor treatment. Here we have designed a series of compounds, methyl 5-sulfamoyl-benzoates, which bear a primary sulfonamide group, a well-known marker of CA inhibitors, and determined their affinities for all twelve CA isozymes. Variations of substituents on the benzenesulfonamide ring led to compound **4b**, which exhibited an extremely high *observed* binding affinity to CAIX; the *K_d_* was 0.12 nM. The *intrinsic* dissociation constant, where the binding-linked protonation reactions have been subtracted, reached 0.08 pM. The compound also exhibited more than 100-fold selectivity over the remaining CA isozymes. The X-ray crystallographic structure of compound **3b** bound to CAIX showed the structural position, while several structures of compounds bound to other CA isozymes showed structural reasons for compound selectivity towards CAIX. Since this series of compounds possess physicochemical properties suitable for drugs, they may be developed for anticancer therapeutic purposes.

## 1. Introduction

Low-molecular-weight compounds that strongly interact and inhibit the disease target enzymes may be developed as therapeutic drugs. The carbonic anhydrase (CA) family (CA, EC 4.2.1.1), consisting of twelve catalytically active human isozymes, performs the catalysis of reversible hydration of CO_2_ into bicarbonate and acid protons. These enzymes participate in numerous physiological processes such as pH regulation and carbon metabolism and are also related to various diseases and conditions such as glaucoma, epilepsy, cancer, edema, osteoporosis, and obesity [1,2]. Since it has been observed that CAIX isozyme is highly overexpressed in numerous cancers, it has been postulated that compounds inhibiting CAIX may possess the anticancer activity and may be used in tumor treatment [3,4,5].

Many CA inhibitors have been synthesized bearing flexible tails capable of finding an energetically favorable position in several CA isozymes, thus losing selectivity [6,7]. We have attempted to design inhibitors with a restrained position of the ring and two functional groups attached to the benzenesulfonamide [8,9,10]. This approach helped design inhibitors selective for CAVII, CAIX, CAXII, and CAXIV.

The introduction of a halogen in the *ortho*- position relative to sulfonamide constrained the benzenesulfonamide ring in a stable position in the CA active site. Additional substituents in the *meta*- and *para*-positions provided additional leverage for increased affinity and selectivity [11,12,13]. However, these compounds did not fully exploit differences in the CA active site and did not reach sufficient selectivity.

The isozymes CAIX and CAXII exhibit slightly more opened active site entrance than CAI and CAII because, at the entrance of the binding pocket, CAIX has Val131, and CAXII has Ala131, while CAI and CAII have bulky Tyr204 and Phe131 at isostructural positions [14]. Substituents in the *ortho*-position would be expected to interact with the narrower entrance to the active site closer to the Zn(II) and, therefore, would result in greater selectivity for CAIX and CAXII. We attempted to exploit these differences by designing inhibitors selective for CAIX and CAXII—*ortho*-substituted benzenesulfonamides. Two such compounds were designed to bear bulky groups in *ortho*- or *para*-positions to study the effect of substituent’s position, size, and flexibility. The compounds were synthesized, and binding affinities and selectivities were determined for the entire set of 12 recombinant human CA isozymes. Key compounds were crystallized with several CA isozymes demonstrating their exact structural positions when bound to CAIX and other isozymes that lead to significant changes in affinity and selectivity for a particular isozyme. Several inhibitors possessed subnanomolar affinities for CAIX and greater than 100-fold selectivities over the remaining human CA isozymes. Thus, such compounds could be further developed for therapeutic purposes.

## 2. Results and Discussion

### 2.1. Organic Synthesis of Designed Compounds

**Scheme 1:**
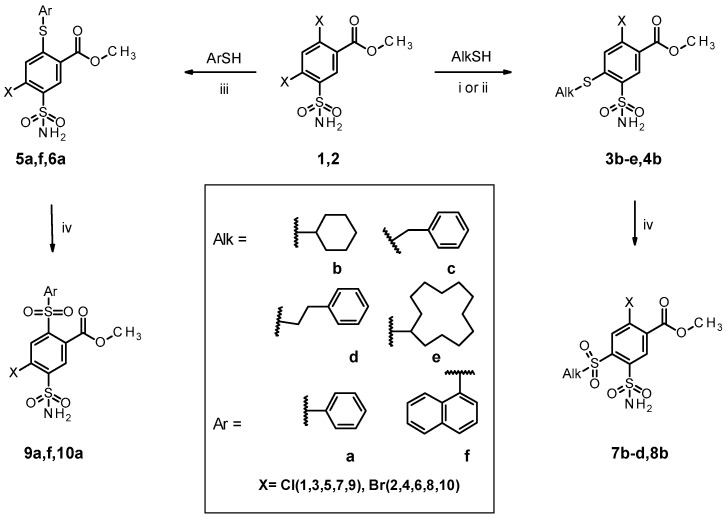
The synthesis of methyl halo 2- and 4-substituted-5-sulfamoyl-benzoates: (i) TEA, DMSO, 60 °C; (ii) K_2_CO_3_, DMSO, 60 °C; (iii) TEA, MeOH, Δ; (iv) H_2_O_2_, AcOH, 75 °C. The legend in the middle explains the structures of alkyl (Ak; **b**, **c**, **d**, **e**) and aromatic (Ar; **a**, **f**) substituents and halogen atoms (Cl—compounds **1**, **3**, **5**, **7**, **9** or Br—**2**, **4**, **6**, **8**, **10**).

Based on substituted halogenated benzenesulfonamide, a series of compounds were designed as potentially high-affinity and selective inhibitors of particular CA isozymes. Starting from methyl 2,4-dichloro-5-sulfamoyl-benzoate **1** and methyl 2,4-dibromo-5-sulfamoyl-benzoate **2**, the designed methyl halo 2- and 4-substituted-5-sulfamoyl-benzoates were synthesized (Scheme 1). Reaction conditions were first chosen according to our previous series where sulfur-containing nucleophiles reacted with dihalosulfamoylbenzamides [9]. The reactions of 2,4-dihalo-5-sulfamoyl-benzoates **1**, **2** with thiols were performed in boiling methanol with triethylamine. However, the reaction was successful only with aromatic thiols (benzenethiol and naphthalene-1-thiol), yielding the *para*-substituted benzenesulfonamides **5**(**a**,**f**) and **6a**. The reaction was very slow or absent, with thiols bearing aliphatic CH_2_ or CH group next to the sulfur atom.

To improve the synthesis and based on the reaction of dihalobenzamide with cyclohexanethiol [9], the polar protic solvent MeOH was replaced with a polar aprotic solvent DMSO. This replacement enabled the synthesis of *ortho*-substituted benzenesulfonamides (**3b**–**e**), **4b** as the main product. However, by-products also formed, including *para*-substituted or even-disubstituted benzenesulfonamides.

The regioselectivity of the nucleophilic aromatic substitution of a halogen in methyl 2,4-dihalo-5-sulfamoyl-benzoates **1**, **2** was investigated. All reactions were repeatedly performed in DMSO with triethylamine at 60 °C temperature for 72 h. Determination of conversion yield, product ratio, and structure identification has been performed by NMR and HPLC/UV/MS (Table 1, and Supplementary material).

Thiols could be arranged in descending order by their reactivity: phenyl ≥ naphthyl > benzyl ≥ ethylphenyl > cyclohexyl ≥ cyclododecyl. The formation of a disubstituted product was observed in the reaction with phenyl-, naphthyl-, and benzyl- thiols. The 2-substituted product was mainly formed in the reactions with aromatic thiols (naphthyl- and phenylthiol), while in the remaining cases, the formation of the 4-substituted product was dominant. In the case of bulky dodecylthiol, only the formation of the 4-substituted isomer was observed.

**Scheme 2:**
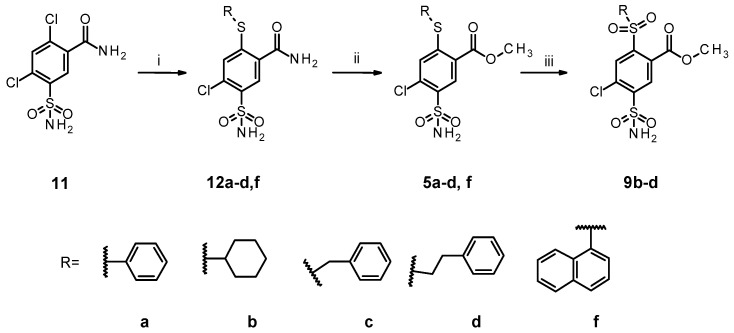
Synthesis of 2-halo-4,5-disubstitutedbenzenesulfonamides: (i) TEA, DMSO, 60 °C; (ii) SOCl_2_, MeOH, Δ; (iii) H_2_O_2_, AcOH, 75 °C.

A group of 2-substituted methyl benzoates **5**(**a**–**d**,**f**) was synthesized via 2,4-dichloro-5-sulfamoyl-benzamide (**11**) (Scheme 2) [9]. The nucleophilic substitution of chlorine occurred only in the *para*- position relative to the sulfonamide group. The amide group of the substituted products **12**(**a**–**d**,**f**) was converted to the ester group using thionyl chloride and MeOH to yield the 2-substituted benzenesulfonamide methyl esters **5**(**a**–**d**,**f**). Oxidation of sulfanyl derivatives **3**(**b**–**d**), **4b**, **5**(**a**–**d**,**f**), **6a** to sulfonyl derivatives **7**(**b**–**d**), **8b**, **9**(**a**–**d**,**f**), **10a** (Scheme 1 and Scheme 2) was carried out using in situ generated peracetic acid.

### 2.2. Thermodynamics of Compound Binding to CA Isozymes

**Figure 1:**
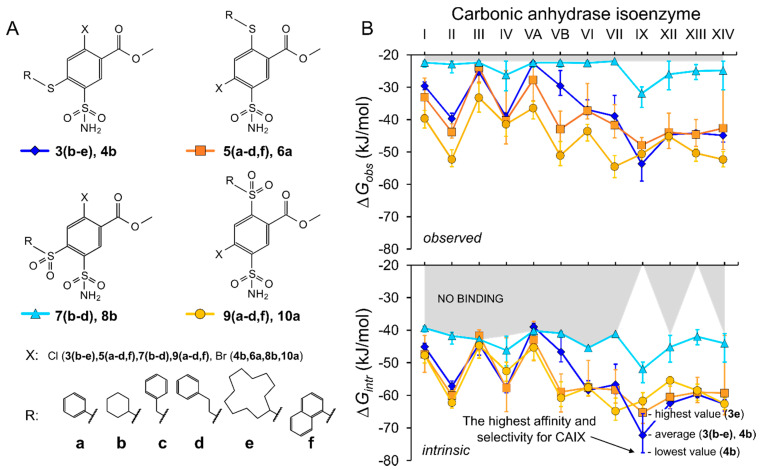
(**A**) Chemical structures of the four groups of compounds—methyl halo 2- or 4-substituted-5-sulfamoyl-benzoates containing chlorine or bromine and substituents listed at the bottom of the panel. (**B**) Averaged change in the standard *observed* (upper panel) or *intrinsic* (lower panel) Gibbs energy for compounds belonging to one of the four groups: **3**(**b**–**e**), **4b**—blue rhombs, **5**(**a**–**d**,**f**), **6a**—orange squares, **7**(**b**–**d**), **8b**—cyan triangles, and **9**(**a**–**d**,**f**), **10a**—yellow circles. The bars show the margin between the strongest and weakest affinity of compounds in that group for the indicated CA isozyme. The *intrinsic* binding affinity of compounds **3**(**b**–**e**), **4b** to CAIX is highlighted: the blue rhombus is the average of all five interactions, the bar above shows the weakest CAIX interaction with **3e**, and below is the strongest with **4b**. Compounds in this group have the highest selectivity and affinity for CAIX.

The chemical structures of 21 compounds used in this study are summarized in Figure 1A. The main goal was to determine the influence of the type and position of the sulfanyl or sulfonyl substituent on the affinity for human CA isozymes. The compounds were divided into four groups with different sulfanyl (-S-) or sulfonyl (-SO_2_-) substituents in *para*- or *ortho*-positions with respect to the sulfonamide group. All compounds had the same ester group in the *meta*-position. The influence of the diversity of the *meta* group on the binding affinity to CAs has been investigated and described in greater detail in a previous publication [9]. We found no significant difference in binding for compounds with chlorine or bromine atoms in that study. Therefore, there is only one analogous brominated compound for comparison (**4b**, **6a**, **8b**, **10a**) in each of the four groups.

Dissociation constants (Table 2) of every compound for each of the 12 catalytically active human CA isozymes were measured using the fluorescent thermal shift assay (FTSA). Some experimental data of selected compounds are provided in Figures S1 and S2 in the Supplementary material. The observed constants (*K_d_obs_*) were converted to the observed standard change in the Gibbs energy of binding (Δ*G_obs_* = *RT*ln *K_d_obs_*, where *R is* the universal ideal gas constant and *T is* the temperature), shown in Figure 1B, upper panel, and Table S1. For visual simplicity, the affinity is averaged for all compounds belonging to one of the four groups and shown in the Figure by coloring the groups: **3**(**b**–**e**), **4b** are blue rhombs, **5**(**a**–**d**,**f**), **6a** are orange squares, **7**(**b**–**d**), **8b** are cyan triangles, and **9**(**a**–**d**,**f**), **10a** are yellow circles (Figure 1B). The bars show the range from the strongest to the weakest affinity for each CA isozyme in that group of compounds (not the error bars).

Interaction of CA with any primary sulfonamide is pH-dependent in identical shape. The affinity is greatest in the near-neutral range and decreases by exactly 10-fold with every pH unit in the acidic and alkaline pH regions. This is explained by the binding mechanism where the deprotonated sulfonamide compound replaces a water molecule (but not a hydroxide ion) coordinated by the Zn(II) in the active site of CA. The deprotonated state of the sulfonamide amino group bound to CA is directly observed by the neutron diffraction structures [15]. However, the sulfonamide state in the solution is predominantly in the protonated form because sulfonamide p*K*_a_ is usually in the region of 9–10. Both the compound and the protein have to undergo protonation–deprotonation reactions to be able to bind. These binding-linked reactions significantly reduce the affinity. Interaction energies of the binding-able states of the protein and ligand are termed *intrinsic*. However, since the protonation reactions occur indistinguishably from protein-ligand binding, we experimentally observe diminished energies, termed *observed*.

The *intrinsic* dissociation constants were calculated according to the model described in the Methods section and Supplementary material using measured p*K_a_* values of the sulfonamide group of the compounds (Table 2). It is more appropriate to use the *intrinsic* parameters in interpreting the interactions, as they help separate the linked protonation reactions that can lead to erroneous conclusions [16]. The *intrinsic* standard change in the Gibbs energy of binding is shown in Figure 1B, lower panel, and Table S2. These parameters were also used in the analysis of crystal structures.

### 2.3. X-ray Crystal Structures and Correlations with Compound Binding Thermodynamics to CA Isozymes

Eight crystal structures of CA complexes with compounds are presented in this study: **9a** bound to CAI and CAII, **3b**, **3d**, **5b**, and **9a** bound to CAXII, **3b** bound to CAIX and mimic-CAIX (mutant of CAII containing amino acids in the active site as in CAIX: A65S, N67Q, I91L, F130V, V134L, and L203A). The electron density maps of the ligands bound in the active site of CA are shown in Figure S3. The data collection and refinement statistics are presented in Table S3. The crystal structures of CAI, CAII (and mimic-CAIX), CAIX, and CAXII complexes in the asymmetric unit contained 2, 1, 4, and 4 protein subunits, respectively.

The structure–thermodynamics correlations that determine the recognition between the CA active site and the ligand will be analyzed in three sections:

(i) methyl 2-halo-4-substituted-5-sulfamoyl-benzoates, abbreviated *ortho*- by substituent at the *ortho*- position relative to sulfonamide group;
(ii) methyl 4-halo-2-substituted-5-sulfamoyl-benzoates, abbreviated as *para*-,
(iii) the position of the substituent in benzenesulfonamide is compared in these two groups of compounds.

#### 2.3.1. Methyl 2-Halo-4-Substituted-5-Sulfamoyl-Benzoates Binding to CA Isozymes

In the group of *ortho*- sulfanyl– **3**(**b**–**e**), **4b**, and sulfonyl– **7**(**b**–**d**), **8b** substituted benzenesulfonamides, the chlorinated **3b**, and brominated **4b** bound similarly. Their *K_d_* differed by less than 3-fold; not a significant difference within the error limits (Table 2). Compounds **7** bound most isozymes with barely detectable affinity, except CAIX, where chlorinated and brominated bound similarly (*K_d_obs_* (**7b**) = 9200 nM and *K_d_obs_* (**8b**) = 6700 nM). Sulfonyl (-SO_2_-, **7**(**b**–**d**), **8b**) compounds bound CA significantly weaker than the parent compound **1** or analogous sulfanyl compounds **3b**–**e**. The strongest interaction with CAIX was likely due to its wider active site than in other CAs [10]. The sulfonyl compounds likely were in an unfavorable conformation for binding and prevented an optimal formation of the sulfonamide group-nitrogen and protein-Zn(II) coordination bond due to steric hindrance.

The p*K_a_* values of the sulfonamide amino group of a series of these compounds were determined. Although the compounds showed limited solubility, the absorption spectra of **7b** and **8b** at different pH were used to determine their p*K_a_* values, 9.8 and 9.9, respectively (Supplementary material, Figure S5). The same, 9.8, value was assigned to other compounds (**7c** and **7d**) in the same group. *Para*-substituted sulfonyl (-SO_2_-) compounds **9**(**a**–**d**,**f**), and **10a** (p*K_a_* = 8.2–8.4) had lower p*K_a_* values than sulfanyl (-S-) compounds **5**(**a**–**d,f**), and **6a** (p*K_a_* = 9.3–9.4), while *ortho*-substituted compounds showed the opposite (Figure 1B, upper panel and Table 2). The p*K_a_* values were 9.9 for sulfonyl (-SO_2_-) compounds **7**(**b**–**d**,**f**), **8b**, and 9.5-9.6 for sulfanyl (-S-) compounds **3**(**b**–**e**), **4b**. Therefore, oxidation did not reduce the p*K_a_* of the sulfonamide group in all cases. The p*K_a_* values were well correlated with chemical shifts determined by NMR (Supplementary material, Figure S6). Chemical shifts show the same trend: values of *ortho*-sulfonyl compounds were lower than sulfanyl, and, in contrast, *para*-sulfonyl compounds had higher values than sulfanyl.

**Figure 2:**
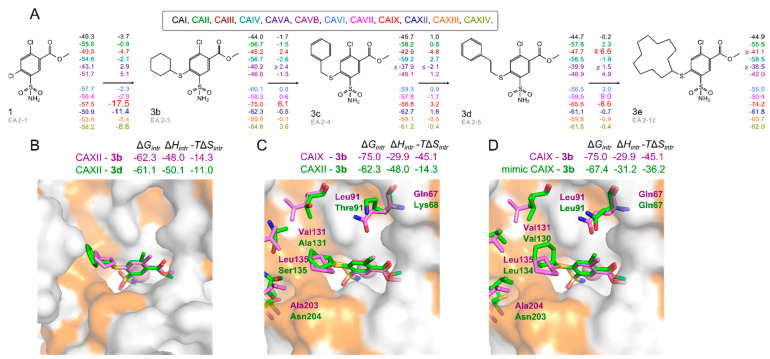
Correlation of compound chemical structures with the changes in standard intrinsic Gibbs energy upon binding and the comparison of the crystal structures between CA isozymes. (**A**) Differences of Δ*G_intr_* between neighboring compounds are listed on the connecting arrows. All values have units of kJ/mol. Colors represent CA isozymes. (**B**) Compounds **3b** (magenta, PDB ID: 7PP9) and **3d** (green, PDB ID: 7PUW) bound to CAXII. (**C**) Compound **3b** bound to CAXII (green, PDB ID: 7PP9) and CAIX (magenta, PDB ID: 7POM). (**D**) Compound **3b** bound to CAIX (magenta, PDB ID: 7POM) and mimic-CAIX (green, PDB ID: 7Q0C). Zinc ion is shown as a pink sphere. The notable nonconservative residues between active sites are shown in the “stick” mode. The protein surface of the CA active site is colored orange for hydrophobic residues (V, I, L, F, M, A, G, and P) and gray for the residues with charged and polar side chains (R, D, N, E, Q, H, K, S, T, Y, W, and C).

Sulfanyl- compounds **3b**–**e** and **4b** bound weakly to CAI, CAIII (narrow entrance to the active site due to Phe198), CAVA, and CAVB. However, compounds had μM affinity to CAII, CAIV, CAVI, CAVII and even approximately 10-fold stronger (*K_d_obs_* is 13–53 μM) to CAXII, CAXIII, and CAXIV (the measured affinity to CAIX is up to 0.12 μM (Table 2 and Figure 1B). Comparison between parent compound **1** and the *ortho*-substituted sulfanyl compounds showed that the hydrophobic substituent had a significant effect (Figure 2). The intrinsic affinity for CAXII (−11.4 kJ/mol), CAXIII (−5.4 kJ/mol), CAXIV (−8.6 kJ/mol), and especially for CAIX (−17.5 kJ/mol or about 1000-fold) significantly increased, and CAVA (2.9 kJ/mol) and CAVB (5.1 kJ/mol) decreased (**1**→**3b**). The affinity strongly depended on the flexibility of the substituent and shorter substituents in compounds **3b** and **3e** had higher affinity to CAIX than longer and more flexible substituents in compounds **3c** and **3d**: the difference was at least about 6 kJ/mol or about 10-fold (Δ*G_intr_* (**3b**) = −75.0 kJ/mol, Δ*G_intr_* (**3c**) = −68.8 kJ/mol, Δ*G_intr_* (**3d**) = −65.6 kJ/mol, Δ*G_intr_* (**3e**) = −74.2 kJ/mol). The affinity for CAVII decreased significantly (9.0 kJ/mol) from compound **3d** to compound **3e**, possibly due to the size of the substituent. In other cases, the differences were within the error margin.

We compared the crystal structures of several compounds in two different CA isozymes (Figure 2B–D). Figure 2B shows the binding mode of **3b** and **3d** in the active site of CAXII. These compounds are structurally similar and differ only in the size of large hydrophobic *ortho* substituents; compound **3b** has the octyl, while **3d** has the 2-phenylethyl moiety. The binding modes of both ligands in the active site of CAXII were the same: *ortho* groups were directed to the hydrophobic part of the active site, while *meta* substituents were orientated to the hydrophilic part. The binding affinities and enthalpy changes to CAXII were almost identical (Δ*G_intr_* = −62.3 kJ/mol, Δ*H_intr_ =* −48.0 kJ/mol (**3b**) vs. Δ*G_intr_* = −61.1 kJ/mol, Δ*H_intr_ =* −50.1 kJ/mol (**3d**), Figure 2B). Experimental data of the Δ*H_obs_* are shown in the Supplementary material in Figure S8.

Figure 2C shows the superposition of **3b** in the active sites of CAIX and CAXII. The positions of the compounds were similar. However, the binding affinities of **3b** to CAIX and CAXII differed significantly and were 150 times stronger to CAIX than CAXII (*K_d_intr_* = 0.030 nM or Δ*G_intr_* = −62.3 kJ/mol to CAXII and *K_d_intr_* 0.20 pM or Δ*G_intr_* = −75.0 kJ/mol to CAIX, Table 2 and Table S2). Again, the side chains interacting with **3b** in CAIX and CAXII were different. They were more hydrophobic in CAIX (A203, L135, V131, L91, and Q67) and more hydrophilic in CAXII (N204, S135, V131, T91, and K68). Thus, these differences could be related to the solvation entropy because binding to CAIX was much more entropy-driven than to CAXII (Δ*H_intr_ =* −29.9 kJ/mol, −*T*Δ*S_intr_ =* −45.1 kJ/mol to CAIX and Δ*H_intr_ =* −48.0 kJ/mol, −*T*Δ*S_intr_ =* −14.3 kJ/mol to CAXII).

The binding of **3b** to CAIX and mimic-CAIX is compared in Figure 2D. Mimic-CAIX is CAII with multiple point mutations that replace the side chains of the active site of CAII with the corresponding residues of CAIX. It has been shown that six-point mutations (A65S, N67Q, I91L, F130V, V134L, and L203A) are sufficient to switch the binding mode of the selective ligand [17,18,19] (mutations slightly different A65S, N67Q, E69T, I91L, F131V, K170E, and L204A). The ligand positions were similar except for the shift of the *ortho* cyclooctyl group. Nevertheless, the native CAIX bound **3b** 10-fold more strongly than the mimic-CAIX (Supplementary material Figure S1). The crystal structure could not answer this question with certainty, but the differences in binding affinities between CAIX and CAII were even greater: Δ*G_intr_* = −56.7 kJ/mol to CAII, Δ*G_intr_* = −67.4 kJ/mol to mimic-CAIX and Δ*G_intr_* = −75.0 kJ/mol to CAIX. The mutations introduced in CAII mimicked the active site of CAIX. The binding affinities were more similar to CAIX than CAII, and very similar enthalpy changes between CAIX and mimic-CAIX were observed: Δ*H_intr_ =* −29.9 kJ/mol to CAIX and Δ*H_intr_ =* −31.2 kJ/mol to mimic-CAIX).

#### 2.3.2. Methyl 4-Halo-2-Substituted-5-Sulfamoyl-Benzoates Binding CA Isozymes

The compounds that contain *para*- and *ortho*- substituents are similar in the weak binding affinity to CAI, CAIII, and CAVA. The binding of *ortho* **3b**–**f**, **4b**, and *para* **5a**–**f**, **6a**, **9a**–**f**, and **10a** was similar to CAII, CAVI, and CAXIII. The affinity of **5a**–**f** (*para*-sulfanyl) to CAVB was even 10-fold higher than of *ortho*-substituted **3b**–**e**, but no significant differences between *para*-substituted sulfanyl (-S-), **5a**–**f**, **6a**, and sulfonyl (-SO_2_-), **9a**–**f**, **10a**, compounds was observed while comparing the intrinsic constants. Thus, only in some cases the oxidation of the sulfanyl group in the *para*- position had an effect on the affinity for several isozymes, namely, CAVII, CAIX, CAXII, and CAXIV (Figure 1B, lower graph). The binding affinity was quite similar for CAII, CAXII, and other isozymes, containing similar amino acids in the active site.

**Figure 3:**
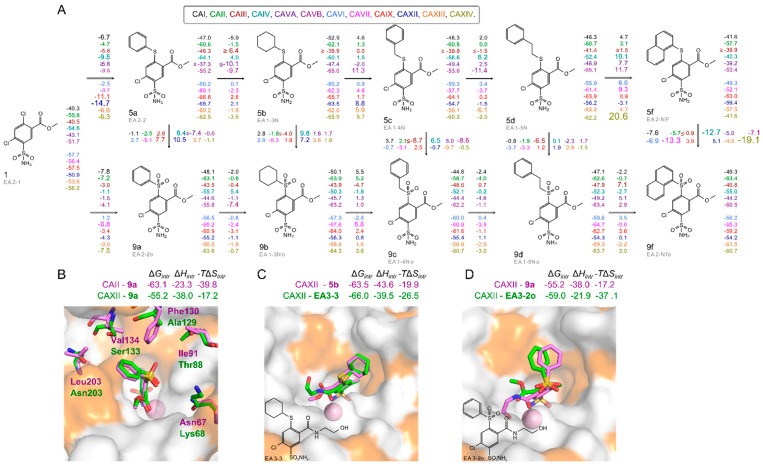
Correlations of compound chemical structures with the changes in the standard intrinsic Gibbs energies of binding and the crystal structures of CAII and CAXII isozymes. (**A**) Differences of Δ*G_intr_* (kJ/mol) between neighboring compounds are listed on the connecting arrows. Colors represent CA isozymes. (**B**) Compound **9a** bound to CAII (PDB ID: 7Q0E) and CAXII (PDB ID: 7PUV). Protein side chains and ligands bound to CAII are colored magenta, CAXII green. The notable nonconservative residues between active sites are shown in the “stick” mode. (**C**) Compounds **5b** (magenta, PDB ID: 7PUU) and **EA3-3** (green, previously studied, PDB ID: 6R6Y) in the active site of CAXII. (**D**) Compounds **9a** (green, PDB ID: 7PUV) and **EA3-2o** (magenta, previously described, PDB ID: 6R71) in the active site of CAXII. Zinc ion is shown as a pink sphere. The protein surface of the CA active site is colored orange for hydrophobic residues (V, I, L, F, M, A, G, and P) and gray for the residues with charged and polar side chains (R, D, N, E, Q, H, K, S, T, Y, W, and C).

Comparing the influence of chlorine and bromine, the binding affinities to all CA isozymes were practically identical between compounds **5a** and **6a**, **9a** and **10a** (Table 2). The influence of the R substituent could not be clearly compared, but compounds with more hydrophobic substituents were weaker binders, e.g., **5d** and **5f**. This was possibly due to solubility issues that were visually apparent at the highest concentrations used in the experiment. There was a slight trend that the compounds bearing less flexible substituents, **5a** and **5b** or **9a** and **9b**, often had higher affinity than **5c**, **5d**, **5f**, and **9c**, **9d**, **9f**. The greater effect was seen for CAVB going from phenyl to cyclohexyl (**5a** → **5b** −9.7 kJ/mol and **9a** →**9b** −7.4 kJ/mol) in Figure 3A. The affinity similarly decreased in vertical transition for CAIV (**5a** → **9a** 8.4 kJ/mol, **5b** → **9b** 9.8 kJ/mol, **5c** → **9c** 6.5 kJ/mol), CAIX (**5b** → **9b** 1.8 kJ/mol, **5c** → **9c** 2.5 kJ/mol, **5d** → **9d** 1.2 kJ/mol).

The X-ray crystal structures showed the energetically best pose of the compound when bound to a CA isozyme. Compound **9a** was found at an identical position in the active sites of CAII and CAXII (Figure 3B). On the other hand, the binding affinity for CAXII differed from CAII by a factor of 26 (*K_d_intr_* = 0.020 nM or Δ*G_intr_* = −63.1 kJ/mol for CAII vs. *K_d_intr_* = 0.51 nM or Δ*G_intr_* = −55.2 kJ/mol for CAXII, Table 2 and Figure 3B). The same binding mode of **9a** in CAII and CAXII could be explained by the same interactions between the compound and conservative residues of these active sites. The active site of CAII is more hydrophobic than CAXII (see residues in “stick” mode shown in Figure 3B). For this reason, the binding of this compound to CAII was 26-fold stronger than for CAXII. The interaction of the phenyl group with the hydrophobic side chains was more favorable. The solvation of the ligand during the dissociation process in the presence of a more hydrophobic environment is more difficult. In this case, the stronger interaction was characterized by a lower enthalpy contribution (Δ*H_intr_ =* −23.3 kJ/mol to CAII, Δ*H_intr_ =* −38.0 kJ/mol to CAXII, Figure 3B). The structure of **9a** was also determined in the active site of CAI and compared with the positions determined in CAII and CAXII (Figure S4). In this case, the results were somewhat inconclusive because two alternative positions of the compound were found in CAI. One position is partially similar to the positions determined in CAII and CAXII, and the alternative is rotated (Figure S4). The **9a** is unlikely to occupy a well-defined position and had a weak binding to CAI (Δ*G_intr_* = −48.1 kJ/mol). Still, it showed a significant change in the enthalpy of binding (Δ*H_intr_ =* −39.7 kJ/mol).

We divided the compounds bound to CAXII into pairs according to their similar binding affinity (Figure 3C,D). Compounds **5b** and **EA3-3** in the active site of CAXII were compared in Figure 3C. Compound **EA3-3** was described in our previous study [9] and was named “4b”. The **5b** is well defined in the crystal structure (Figure S3D). The binding affinities of the two compounds for CAXII differed by a factor of 2.5, which is insignificant: *K_d_intr_* = 0.020 nM, Δ*G_intr_* = −63.5 kJ/mol (**5b**) and *K_d_intr_* = 0.0080 nM, Δ*G_intr_* = −66.0 kJ/mol (**EA3-3**). These compounds differ structurally by *meta*-substituents; **EA3-3** has a longer and more flexible substituent (-NH(CH_2_)_2_OH), but both *meta*- groups were mostly exposed to the solvent, so they did not contribute much to the positioning in the active site. Both compounds are chlorinated at the *ortho*- position of the benzenesulfonamide, and chlorine is known to restrict the position of the ligand in the active site [11,20]. In addition, the *para*-cyclooctyl groups of both compounds occupied the same position. Since the chlorinated compounds had less freedom in the active site, the differences in entropy and enthalpy of the slightly different binding of the *meta*- groups compensated each other: Δ*H_intr_* = −43.6 kJ/mol, -*T*Δ*S_intr_* = −19.9 kJ/mol (**5b**) Δ*H_intr_* = −39.5 kJ/mol, -*T*Δ*S_intr_* = −26.5 kJ/mol (**EA3-3**).

The comparison of **9a** and **EA3-2o** bound to CAXII (described in [9] and named “16a”) is shown in Figure 3D. Like the previous pair, these compounds differ only by their *meta*- groups: compound **EA3-2o** contains -NH(CH_2_)_2_OH, while **9a** contains -OCH_3_. The positions of both compounds were not similar in CAXII; the *para*-benzene rings had slightly different orientations, and they were directed into the center of the active site cavity. Their orientation was defined by the bulky SO_2_ group of the linker, which was in contact with the protein surface. The **EA3-2o** bound to CAXII with 3.4-fold greater affinity than **9a** (*K_d_intr_* = 0.12 nM, Δ*G_intr_* = −59.0 kJ/mol vs. *K_d_intr_* = 0.51 nM Δ*G_intr_* = −55.2 kJ/mol). The differences of binding modes between compounds were found in the position of both *meta*- and *para*- groups, but the different terms of the binding reaction mutually compensated each other: Δ*H_intr_* = −38.0 kJ/mol, -*T*Δ*S_intr_* = −17.2 kJ/mol (**9a**) Δ*H_intr_* = −21.9 kJ/mol, -*T*Δ*S_intr_* = −37.1 kJ/mol (**EA3-2o**).

The pairs of compounds bound to the same CA isozyme analyzed in Figure 2D and Figure 3C,D had the following common features: the positions and the binding affinities of the compounds in the pairs were the same or similar. Analogous observations were made previously [21], and such pairs were called “similar” binders. In pairs of “similar” binders, the additional hydrophobic surface did not produce additional interactions with the active site of CA. The possible origin of the entropy–enthalpy compensation was the changes in the solvation–desolvation processes of ligands and the active sites.

Comparison of compound binding to different isozymes when the differences of binding affinities were experimentally significant (Figure 2C,D and Figure 3A) showed some similarities. The compound occupied a similar position in the active site of different isozymes, and the binding of the ligand to a more hydrophobic site was stronger.

#### 2.3.3. Comparison of Methyl Halo 2- and 4-Substituted-5-Sulfamoyl-Benzoates Binding to CA Isozymes

**Figure 4:**
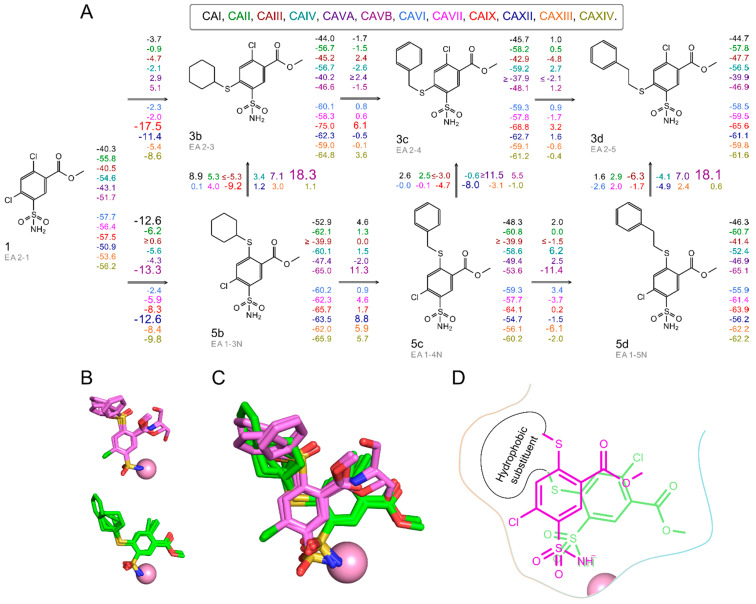
Correlation of compound chemical structures with the changes in standard intrinsic Gibbs energy upon binding and comparison of the crystal structures between CA isozymes. (**A**) Differences of Δ*G_intr_* (kJ/mol) between neighboring compounds are listed on the connecting arrows. Colors represent CA isozymes. (**B**) Superimposed ligands of these crystal structures: (upper panel) *para*-substituted ligands are colored magenta: CAXII—**3b** (PDB ID: 7PP9), CAXII—**3d** (PDB ID: 7PUW), CAIX—**3b** (PDB ID: 7POM), mimic-CAIX—**3b** (PDB ID: 7Q0C), (lower panel) *ortho*-substituted ligands are colored green: CAXII—**EA3-3** (PDB ID: 6R6F), CAXII—**EA3-2o** (PDB ID: 6R71), CAXII—**5b** (PDB ID: 7PUU), CAXII—**9a** (PDB ID: 7PUV), CAII—**9a** (PDB ID: 7Q0E). (**C**) The abovementioned superimposed structures are shown for comparison. (**D**) Scheme representing compound position shown in panel C. Orange line represents more hydrophobic side of the active site. Zinc ion is shown as a pink sphere.

The crystal structures discussed below suggested that the binding interactions between *ortho*- and *para*-sulfanyl/sulfonyl compounds with several CA were quite different. Various *ortho*- and *para*-substituted compounds had different affinities for CA isozymes. Figure 4A compares the affinity of analogous sulfanyl compounds for CA isozymes, and panels B and C compare the positions of these compounds in different crystal structures. The transition from **1** to **3b** and **5b** shows different changes in affinity and selectivity; compound **3b** bound distinctly more strongly to CAIX (−17.5 kJ/mol), CAXII (-11.4 kJ/mol), whereas compound **5b** bound more strongly to CAI (−12.6 kJ/mol), CAVB (−13.3 kJ/mol) and CAXII (−12.6 kJ/mol). The influence of the hydrophobic R substituent in the same group (transition from left to right (→) has already been discussed above, but in some cases, the transition from *para*- to *ortho*-positions (↑) showed similar trends and was in all cases favorable only for binding to CAIX. However, a change in the position of more flexible substituents (**5c**→**3c**: 2.6 kJ/mol and **5d**→**3d**: 1.6 kJ/mol) had a lower negative effect on the binding affinity to CAI than a rigid cyclohexyl (**5b**→**3b**: 8.9 kJ/mol).

Figure 4B upper part shows 5 superimposed structures: CAXII—**EA3-3** (PDB ID: 6R6Y), CAXII—**EA3-2o** (PDB ID: 6R71), CAXII—**5b** (PDB ID: 7PUU), CAXII—**9a** (PDB ID: 7PUV), CAII—**9a** (PDB ID: 7Q0E); and 4 structures in the lower part: CAXII—**3b** (PDB ID: 7PP9), CAXII—**3d** (PDB ID: 7PUW), CAIX—**3b** (PDB ID: 7POM), mimic CAIX—**3b** (PDB ID: 7Q0C). Therefore, regardless of the compound, all *ortho*-compounds occupied a similar position in CAIX and CAXII, and *para*- occupied another but similar position to each other in CAII and CAXII (Figure 4C,D, respectively). Despite all the differences in the substituents and amino acids in the active sites, the main frame of the compound depicted in the scheme in Figure 4D maintained a similar position. The position of hydrophobic substituents varied, but in the optimal conformation, it was close to the hydrophobic part of the active site. The *meta* substituents were not analyzed in this study, but a comparison with previously published compounds shows that the hydrophilic substituents in these cases did not result in a different binding mode and were flexible. Thus, the hydrophobic effect plays a key role in enabling the compounds to be in the optimal position.

## 3. Conclusions

We have designed a series of compounds and investigated the effect of substituents and their positions in methyl halo 2- and 4-substituted-5-sulfamoyl-benzoate binding to human CA isozymes. The sulfanyl (-S) and sulfonyl (-SO_2_-) substituents of different sizes and hydrophobicity were examined in greater detail. We provided a crystallographic position of these two groups of compounds bound in the active sites of CA. Although there were differences in the orientation of the substituents and the thermodynamic parameters, the positions in the same group of compounds remained similar among CA isozymes.

The strongest binding *ortho*-sulfanyl-substituted benzenesulfonamides, especially compounds **3b** and **4b**, showed extremely high femtomolar *intrinsic* affinity (80 fM, corresponding to very high, 120 pM *observed* affinity) and selectivity for tumor-associated CAIX. Meanwhile, analogous sulfonyl compounds bound weakly. Other *para*-substituted compounds had completely different binding profiles and bound similarly to several CA isozymes not demonstrating such selectivity.

The crystal structures of the complexes containing compounds bound to CAI, CAII, mimic-CAIX (mutant of CAII^A65S, N67Q, I91L, F130V, V134L, L203A^), CAIX, and CAXII provided a broader understanding of the differences and similarities in the thermodynamic parameters of binding. We observed a tendency that interaction, where there was a higher contribution of the hydrophobic effect and higher entropy contribution, usually also had a higher affinity. This may be explained by the fact that (i) the hydrophobic side chains and bulky hydrophobic substituents of the ligand prevented water molecules from entering the cavity, leading to stronger interaction, and (ii) the water molecules could penetrate between the ligand and the active site cavity through the hydrophilic surface part of the active site. Water molecules in the deeper part of the active site could compete with the compound and cause weaker interaction than in the first case.

## 4. Materials and Methods

### 4.1. Organic Synthesis

All starting materials and reagents were commercial products and were used without further purification. Melting points of the compounds were determined in open capillaries on a Thermo Scientific 9100 Series and are uncorrected. ^1^H and ^13^C NMR spectra were recorded on a (400 and 100 MHz, respectively) spectrometer in DMSO-d_6_ using residual DMSO signals (2.52 ppm and 40.21 ppm for ^1^H and ^13^C NMR spectra, respectively) as the internal standard. TLC was performed with silica gel 60 F254 aluminum plates (Merck) and visualized with UV light. Column chromatography was performed using silica gel 60 (0.040–0.063 mm, Merck). High-resolution mass spectra (HRMS) were recorded on a Dual-ESI Q-TOF 6520 mass spectrometer (Agilent Technologies). The purity of final compounds was verified by HPLC to be >95% using the Agilent 1290 Infinity instrument with a Poroshell 120 SB-C18 (2.1 mm × 100 mm, 2.7 μm) reversed-phase column. Analytes were eluted using a linear gradient of water/methanol (20 mM ammonium formate in both phases) from 60:40 to 30:70 over 12 min, then from 30:70 to 20:80 over 1 min, and then 20:80 over 5 min at a flow rate of 0.2 mL/min. UV detection was at 254 nm.

### 4.2. General Procedure for the Syntheses of ***1***, ***2***

The appropriate 2,4-dichloro-5-sulfamoylbenzoic acid or 2,4-dibromo-5-sulfamoylbenzoic acid [8] (10.0 mmol) was refluxed in MeOH (100 mL) with concentrated H_2_SO_4_ (1 mL) for 20 h. The reaction mixture was concentrated under reduced pressure.

*Methyl 2,4-dichloro-5-sulfamoyl-benzoate (***1**, *EA2-1).* Recrystallization was accomplished from MeOH. Yield: 2.70 g, 95%, mp 202 °C. ^1^H NMR δ ppm: 3.91 (3H, s, CH_3_), 7.87 (2H, s, SO_2_NH_2_), 8.04 (1H, s, C_3_-H), 8.40 (1H, s, C_6_-H). ^13^C NMR δ ppm: 53.5, 128.8, 131.7, 134.0, 135.0, 136.8, 140.5, 164.1. HRMS calculated for C_8_H_7_Cl_2_NO_4_S [(M+H)^+^]: 283.9546, found: 283.9546.

*Methyl 2,4-dibromo-5-sulfamoyl-benzoate (***2**, *LJ14-4).* Recrystallization was accomplished from MeOH. Yield: 2.35 g, 63%, mp 201–203 °C. ^1^H NMR δ ppm: 3.90 (3H, s, CH_3_), 7.84 (2H, s, SO_2_NH_2_), 8.33 (1H, s, C_6_-H), 8.35 (1H, s, C_3_-H). ^13^C NMR δ ppm: 53.6, 123.6, 125.2, 131.3, 131.6, 140.2, 142.8, 164.9. HRMS calculated for C_8_H_7_Br_2_NO_4_S[(M+H)^+^]: 373.8515 (100%), found: 373.8514 (100%).

### 4.3. General Procedure for the Syntheses of ***3b***, ***3e***, and ***4b***

The mixture of appropriate methyl 2,4-dihalogeno-5-sulfamoylbenzoate (compounds **1**,**2**) (1.00 mmol), DMSO (2 mL), appropriate cyclohexanethiol or cyclododecanethiol (1.10 mmol), and K_2_CO_3_ (553 mg, 4.00 mmol) was heated at 60 °C temperature for 4–6 h. The mixture was cooled to room temperature, and brine was added. The product was extracted with EtOAc (3 × 7 mL). The organic layer was washed with H_2_O, dried over anhydrous MgSO_4_, filtered, and concentrated.

*Methyl 2-chloro-4-(cyclohexylsulfanyl)-5-sulfamoylbenzoate (***3b***, EA2-3).* The product was purified by chromatography on a column of silica gel with CHCl_3_:EtOAc (10:1), R_f_ = 0.40. Yield: 131 mg, 36%, mp 112–114 °C. ^1^H NMR δ ppm: 1.21–1.31 (1H, m, Cy-H), 1.38–1.50 (4H, m, Cy-H), 1.60–1.63 (1H, m, Cy-H), 1.70–1.77 (2H, m, Cy-H), 1.93–2.01 (2H, m, Cy-H), 3.71–3.77 (1H, m, Cy-H), 3.88 (3H, s, CH_3_), 7.56 (2H, s, SO_2_NH_2_), 7,71 (1H, s, C_3_-H), 8.33 (1H, s, C_6_-H).^13^C NMR δ ppm: 25.6, 25.7, 32.4, 44.3, 53.2, 125.1, 130.3, 131.5, 136.4, 140.2, 142.9, 164.4. HRMS calculated. for C_14_H_18_ClNO_4_S_2_[(M+H)^+^]: 364.0439, found: 364.0440.

*Methyl 2-chloro-4-(cyclododecylsulfanyl)-5-sulfamoylbenzoate (***3e***, EA2-12).* The product was purified by chromatography on a column of silica gel with CHCl_3_:EtOAc (5:1), R_f_ = 0.65, then recrystallization was accomplished from toluene:EtOAc (8:1). Yield: 210 mg, 47%, mp 185–187 °C. ^1^H NMR δ ppm: 1.25–1.64 (20H, m, cyclododecyl-H), 1.71–1.83 (2H, m, cyclododecyl-H), 3.71–3.79 (1H, m, cyclododecyl-H), 3.88 (3H, s, CH_3_), 7.56 (2H, s, SO_2_NH_2_), 7.67 (1H, s, C_3_-H), 8.33 (1H, s, C_6_-H). ^13^C NMR δ ppm: 22.1, 23.3, 23.4, 23.8, 24.1, 29.2, 43.2, 53.2, 124.9, 130.3, 131.4, 136.3, 140.3, 143.5, 164.4. HRMS calculated for C_20_H_30_ClNO_4_S_2_[(M+H)^+^]: 448.1378, found: 448.1370.

*Methyl 2-bromo-4-(cyclohexylsulfanyl)-5-sulfamoylbenzoate (***4b***, LJ15-12).* The product was purified by chromatography on a column of silica gel with CHCl_3_:EtOAc (15:1), R_f_ = 0.29. Yield: 180 mg, 44%, mp123–125 °C. ^1^H NMR δ ppm: 1.24–1.31 (1H, m, Cy-H), 1.38–1.50 (4H, m, Cy-H), 1.60–1.63 (1H, m, Cy-H), 1.70–1.75 (2H, m, Cy-H), 1.95–1.97 (2H, m, Cy-H), 3.72–3.77 (1H, m, Cy-H), 3.88 (3H, s, CH_3_), 7.54 (2H, s, SO_2_NH_2_), 7.85 (1H, s, C_6_-H), 8.27 (1H, s, C_3_-H). ^13^C NMR δ ppm: 25.6, 25.7, 32.4, 44.5, 53.2, 125.1, 127.5, 131.0, 133.6, 140.1, 142.5, 165.0. HRMS calculated for C_14_H_18_BrNO_4_S_2_[(M+H)^+^]: 409.9913 (100%), found: 409.9915 (100%).

### 4.4. General Procedure for the Syntheses of ***3c*** and ***3d***

The mixture of methyl 2,4-dichloro-5-sulfamoylbenzoate (**1**) (284 mg, 1.00 mmol), DMSO (2 mL), appropriate phenylmethanethiol or 2-phenylethanethiol (1.10 mmol), and TEA (121 mg, 1.20 mmol) was heated at 50 °C temperature for 6–12 h. The progress of the reaction was monitored by TLC. The mixture was cooled to room temperature, and brine was added. The product was extracted with EtOAc (3 × 7 mL). The organic layer was washed with H_2_O, dried over anhydrous MgSO_4_, filtered, and concentrated.

*Methyl 4-(benzylsulfanyl)-2-chloro-5-sulfamoylbenzoate (***3c***, EA2-4).* The product was purified by chromatography on a column of silica gel with CHCl_3_:EtOAc (4:1), R_f_ = 0.48. Yield: 119 mg, 32%, mp 125–126 °C. ^1^H NMR δ ppm: 3.87 (3H, s, CH_3_), 4.50 (2H, s, CH_2_), 7.29 (1H, t, *J =* 7.2 Hz, Ph-H), 7.36 (2H, t, *J =* 7.2 Hz, Ph-H), 7.51 (2H, d, *J =* 7.2 Hz, Ph-H), 7.64 (2H, s, SO_2_NH_2_), 7.69 (1H, s, C_3_-H), 8.31 (1H, s, C_6_-H). ^13^C NMR δ ppm: 36.3, 53.2, 124.9, 128.0, 129.1, 129.2, 129.8, 131.3, 135.8, 136.4, 139.1, 143.7, 164.3. HRMS calculated for C_15_H_14_ClNO_4_S_2_ [(M+H)^+^]: 372.0126, found: 372.0125.

*Methyl 2-chloro-4-[(2-phenylethyl)sulfanyl]-5-sulfamoylbenzoate (***3d***, EA2-5).* The product was purified by chromatography on a column of silica gel with CHCl_3_:EtOAc (5:1), R_f_ = 0.67. Yield: 97 mg, 25%, mp 111–112 °C. ^1^H NMR δ ppm: 2.97 (2H, t, *J =* 7.6 Hz, CH_2_Ph), 3.46 (2H, t, *J =* 7.6 Hz, CH_2_S), 3.88 (3H, s, CH_3_), 7.23 (1H, m, Ph-H), 7.29–7.33 (4H, m, Ph-H), 7.59 (2H, s, SO_2_NH_2_), 7.65 (1H, s, C_3_-H), 8.31 (1H, s, C_6_-H). ^13^C NMR δ ppm: 33.3, 34.1, 53.2, 124.8, 126.9, 128.9, 129.1, 129.2, 131.2, 136.5, 139.4, 140.0, 143.9, 164.4. HRMS calculated for C_16_H_16_ClNO_4_S_2_ [(M+H)^+^]: 386.0282, found: 386.0282.

### 4.5. General Procedure for the Syntheses of ***5a***, ***5f***, and ***6a***

The mixture of appropriate 2,4-dihalogeno-5-sulfamoylbenzoate (compounds **1**,**2**) (1.00 mmol), MeOH (5 mL), thiophenol or 1-thionaphthol (1.10 mmol,) and TEA (121 mg, 1.20 mmol) was refluxed for 2–6 h. MeOH was evaporated under reduced pressure, and the resultant precipitate was washed with H_2_O.

*Methyl 4-chloro-2-(phenylsulfanyl)-5-sulfamoylbenzoate (***5a***, EA2-2).* The product was purified by chromatography on a column of silica gel with CHCl_3_:EtOAc (10:1), R_f_ = 0.32. Yield: 161 mg, 45%, mp 223–226 °C. ^1^H NMR δ ppm: 3.94 (3H, s, CH_3_), 6.68 (1H, s, C_3_-H), 7.62–7.67 (5H, m, Ph-H), 7.74 (2H, s, SO_2_NH_2_), 8.50 (1H, s, C_6_-H). ^13^C NMR δ ppm: 53.3, 124.3, 128.7, 130.1, 131.2 (2C), 131.8, 135.2, 136.2, 137.5, 149.6, 164.8. HRMS calculated for C_14_H_12_ClNO_4_S_2_ [(M+H)^+^]: 357.9969, found: 357.9970.

*Methyl 4-chloro-2-(1-naphthylsulfanyl)-5-sulfamoyl-benzoate (***5f***, EA2-NF).* The product was purified by chromatography on a column of silica gel with CHCl_3_:EtOAc (5:1), R_f_ = 0.77. Yield: 265 mg, 65%, mp 235–237 °C. ^1^H NMR δ ppm: 3.99 (3H, s, CH_3_), 6.34 (1H, s, C_3_-H), 7.61–7.68 (2H, m, Naph-H), 7.70 (2H, s, SO_2_NH_2_), 7.71–7.74 (1H, m, Naph-H), 8.04 (1H, dd, *J* =7.2 Hz, *J* =1.2 Hz, Naph-H), 8.08–8.15 (2H, m, Naph-H), 8.26 (1H, d, *J* = 8.4 Hz, Naph-H), 8.54 (1H, s, C_6_-H). ^13^C NMR δ ppm: 53.4, 124.3, 125.0, 126.7, 127.2, 127.6, 128.5, 128.8, 129.8, 132.0, 132.6, 133.8, 134.6, 135.1, 136.9, 137.5, 148.9, 164.9. HRMS calculated for C_18_H_14_ClNO_4_S_2_ [(M+H)^+^]: 408.0126, found: 408.0131.

*Methyl 4-bromo-2-(phenylsulfanyl)-5-sulfamoylbenzoate (***6a***, LJ15-11).* The product was purified by chromatography on a column of silica gel with CHCl_3_:EtOAc (15:1), R_f_ = 0.20, then recrystallization was accomplished from toluene. Yield: 145 mg, 36%, mp 202–204 °C. ^1^H NMR δ ppm: 3.93 (3H, s, CH_3_), 6.89 (1H, s, C_3_-H), 7.58–7,63 (5H, m, Ph-H), 7.71 (2H, s, SO_2_NH_2_), 8.52 (1H, s, C_6_-H). ^13^C NMR δ ppm: 53.3, 124.3, 124.8, 130.2, 131.1(2C), 131.7, 132.3, 136.1, 139.3, 149.2, 165.0. HRMS calculated for C_14_H_12_BrNO_4_S_2_[(M+H)^+^]: 403.9443 (100%), found: 403.9437 (100%).

### 4.6. General Procedure for the Syntheses of ***7b***–***d***, ***8b***, ***9a***–***d***, ***9f***, ***10a***

The 30% H_2_O_2_(aq) (1.50 mmol, 0.148 mL) was added to a solution of appropriate benzenesulfonamide (compounds **3b**–**d**,**4b**,**5a**–**d**,**f**,**6a**) (0.500 mmol) in AcOH (1.7 mL) at 70 °C and allowed stirring for 2–3h. The solvent was removed under reduced pressure, and the resultant precipitate was washed with H_2_O.

*Methyl 2-chloro-4-cyclohexylsulfonyl-5-sulfamoylbenzoate (***7b***, EA2-3o).* Yield: 164 mg, 83%, mp 184–187 °C. ^1^H NMR δ ppm: 1.14–1.21 (3H, m, Cy-H), 1.42–1.50 (2H, m, Cy-H), 1.63 (1H, br s, Cy-H), 1.81–1.87 (4H, m, Cy-H), 3.89–3.92 (1H, m, Cy-H), 3.96 (3H, s, CH_3_), 7.48 (2H, s, SO_2_NH_2_), 8.11 (1H, s, C_3_-H), 8.57 (1H, s, C_6_-H). ^13^C NMR δ ppm: 24.9 (2C), 25.1, 53.9, 62.2, 133.3, 135.1, 135.5, 136.7, 138.6, 142.1, 163.9. HRMS calculated for C_14_H_18_ClNO_6_S_2_ [(M+H)^+^]: 396.0337, found: 396.0336.

*Methyl 4-benzylsulfonyl-2-chloro-5-sulfamoylbenzoate (***7c***, EA2-4o).* Recrystallization was accomplished from MeOH. Yield: 123 mg, 61%, mp 154–156 °C. ^1^H NMR δ ppm: 3.95 (3H, s, CH_3_), 5.04 (2H, s, CH_2_), 7.24–7.26 (2H, m, Ph-H), 7.33–7.40 (3H, m, Ph-H), 7.58 (2H, s, SO_2_NH_2_), 7.77 (1H, s, C_3_-H), 8.57 (1H, s, C_6_-H).^13^C NMR δ ppm: 53.9, 60.9, 127.5, 129.1, 129.5, 131.7, 132.9, 135.0, 135.4, 136.3, 139.2, 142.0, 163.8. HRMS calculated for C_15_H_14_ClNO_6_S_2_ [(M+H)^+^]: 404.0024, found: 404.0023.

*Methyl 2-chloro-4-(2-phenylethylsulfonyl)-5-sulfamoylbenzoate (***7d***, EA2-5o).* Recrystallization was accomplished from MeOH. Yield: 161 mg, 77%, mp 132–134 °C. ^1^H NMR δ ppm: 3.03 (2H, t, *J =* 7.8 Hz, CH_2_), 3.95 (3H, s, CH_3_), 4.08 (2H, t, *J =* 7.8 Hz, CH_2_), 7.13–7.27 (5H, m, Ph-H), 7.50 (2H, s, SO_2_NH_2_), 8.07 (1H, s, C_3_-H), 8.49 (1H, s, C_6_-H). ^13^C NMR δ ppm: 28.0, 53.9, 55.8, 127.1, 128.9(2C), 132.8, 135.0, 135.3, 136.7, 137.7, 140.0, 141.6, 163.9. HRMS calculated for C_16_H_16_ClNO_6_S_2_ [(M+H)^+^]: 418.0180, found: 418.0175.

*Methyl 2-bromo-4-cyclohexylsulfonyl-5-sulfamoylbenzoate (***8b***, LJ15-12o).* Yield: 172 mg, 78%, mp 217–219 °C. ^1^H NMR δ ppm: 1.16–1.24 (3H, m, Cy-H), 1.42–1.50 (2H, m, Cy-H), 1.57–1.63 (1H, m, Cy-H), 1.79–1.86 (4H, m, Cy-H), 3.89–3.92 (1H, m, Cy-H), 3.95 (3H, s, CH_3_), 7.47 (2H, s, SO_2_NH_2_), 8.25 (1H, s, C_3_-H), 8.50 (1H, s, C_6_-H). ^13^C NMR δ ppm: 24.9(2C), 25.1, 53.9, 62.2, 125.3, 132.8, 137.7, 138.1, 138.4, 142.6, 164.6. HRMS calculated for C_14_H_18_BrNO_6_S_2_[(M+H)^+^]: 441.9811 (100%), found: 441.9806 (100%).

*Methyl 2-(benzenesulfonyl)-4-chloro-5-sulfamoylbenzoate (***9a***, EA2-2No).* The product was purified by chromatography on a column of silica gel with CHCl_3_:EtOAc (4:1), R_f_ = 0.33. Yield: 158 mg, 81%, mp 166–168 °C. ^1^H NMR δ ppm: 3.86 (3H, s, CH_3_), 7.68 (2H, t, *J =* 7.6 Hz, Ph-H), 7.78 (1H, t, *J =* 7.6 Hz, Ph-H), 8.03 (2H, s, SO_2_NH_2_), 8.07 (2H, d, *J =* 7.6 Hz, Ph-H), 8.23 (1H, s, C_3_-H), 8.51 (1H, s, C_6_-H). ^13^C NMR δ ppm: 53.8, 128.5, 130.1, 130.3, 131.7, 133.5, 134.2, 134.9, 140.0, 142.3, 146.0, 165.7. HRMS calculated for C_14_H_12_ClNO_6_S_2_ [(M+H)^+^]: 389.9867, found: 389.9869.

*Methyl 4-chloro-2-cyclohexylsulfonyl-5-sulfamoyl-benzoate (***9b***, EA1-3No).* Yield: 150 mg, 76%, mp 181–183 °C. ^1^H NMR δ ppm: 1.18–1.30 (3H, m, Cy-H), 1.41–1.50 (2H, m, Cy-H), 1.62–1.66 (1H, m, Cy-H), 1.80–1.91 (4H, m, Cy-H), 3.64–3.70 (1H, m, Cy-H), 3.90 (3H, s, CH_3_), 8.06 (2H, s, SO_2_NH_2_), 8.12 (1H, s, C_3_-H), 8.30 (1H, s, C_6_-H). ^13^C NMR δ ppm: 24.8, 25.0, 25.1, 54.0, 62.9, 130.5, 132.6, 133.8, 134.1, 140.1, 145.9, 165.9. HRMS calculated for C_14_H_18_ClNO_6_S_2_ [(M+H)^+^]: 396.0337, found: 396.0341.

*Methyl 2-benzylsulfonyl-4-chloro-5-sulfamoylbenzoate (***9c***, EA1-4No).* Yield: 162 mg, 80%, mp 224–225 °C. ^1^H NMR δ ppm: 3.97 (3H, s, CH_3_), 4.97 (2H, s, CH_2_), 7.27–7.30 (2H, m, Ph-H), 7.37–7.42 (3H, m, Ph-H), 7.79 (1H, s, C_3_-H), 8.07 (2H, s, SO_2_NH_2_), 8.31 (1H, s, C_6_-H). ^13^C NMR δ ppm: 54.2, 61.7, 127.7, 129.1, 129.4, 130.4, 131.7, 132.1, 133.6, 133.9, 140.9, 145.9, 166.0. HRMS calculated for C_15_H_14_ClNO_6_S_2_ [(M+H)^+^]: 404.0024, found: 404.0018.

*Methyl 4-chloro-2-(2-phenylethylsulfonyl)-5-sulfamoylbenzoate (***9d***, EA1-5o).* Recrystallization was accomplished from MeOH. Yield: 142 mg, 68%, mp 156–158 °C. ^1^H NMR δ ppm: 3.06 (2H, t, *J =* 7.6 Hz, CH_2_), 3.91 (3H, s, CH_3_), 4.00 (2H, t, *J =* 8.0 Hz, CH_2_), 7.16–7.20 (1H, m, Ph-H) 7.21–7.27 (4H, m, Ph-H), 8.03 (1H, s, C_3_-H), 8.04 (2H, s, SO_2_NH_2_), 8.26 (1H, s, C_6_-H). ^13^C NMR δ ppm: 28.2, 54.1, 56.5, 127.2, 128.89, 128.92, 130.3, 131.8, 133.8, 133.9, 137.8, 141.5, 145.8, 165.9. HRMS calculated for C_16_H_16_ClNO_6_S_2_ [(M+H)^+^]: 418.0180, found: 418.0178.

*Methyl 4-chloro-2-(1-naphthylsulfonyl)-5-sulfamoylbenzoate (***9f***, EA2-NFo).* Yield: 180 mg, 82%, mp 244–245 °C. ^1^H NMR δ ppm: 3.72 (3H, s, CH_3_), 7.66–7.75 (2H, m, Naph-H), 7.81 (1H, t, *J =* 7.6 Hz, Naph-H), 8.00 (2H, s, SO_2_NH_2_), 8.17 (1H, d, *J =* 8.0 Hz, Naph-H), 8.23 (1H, s, C_3_-H), 8.35 (1H, s, C_6_-H), 8.36 (2H, t, *J* = 8.0 Hz, Naph-H), 8.41 (1H, d, *J* = 8.4 Hz, Naph-H).^13^C NMR δ ppm: 53.8, 123.5, 125.2, 127.7, 127.8, 129.5, 130.1, 130.6, 131.5, 131.6, 132.4, 133.9, 134.0, 134.2, 136.4, 142.4, 145.8, 165.4. HRMS calculated for C_18_H_14_ClNO_6_S_2_ [(M+H)^+^]: 440.0024,found: 440.0020.

*Methyl 2-(benzenesulfonyl)-4-bromo-5-sulfamoylbenzoate (***10a***, LJ15-11o).* Yield: 189 mg, 87%, mp 191–193 °C. ^1^H NMR δ ppm: 3.86 (3H, s, CH_3_), 7.67-7.79 (3H, m, Ph-H), 7.98 (2H, s, SO_2_NH_2_), 8.04–8.07 (2H, m, Ph-H), 8.22 (1H, s, C_6_-H), 8.62 (1H, s, C_3_-H). ^13^C NMR δ ppm: 53.8, 122.7, 128.5, 130.1, 130.2, 132.2, 134.9, 136.7, 140.0, 141.9, 147.8, 165.8. HRMS calculated for C_14_H_12_BrNO_6_S_2_[(M+H)^+^]: 435.9342 (100%), found: 435.9345 (100%).

### 4.7. General Procedure for the Syntheses of ***12a***–***d***,***f***

The mixture of methyl 2,4-dichloro-5-sulfamoylbenzamide (**11**) [8] (269 mg, 1.00 mmol), DMSO (1.5 mL), appropriate thiol(1.10 mmol), and TEA (121 mg, 1.20 mmol) was heated at 60 °C temperature for 12 h. The mixture was cooled to room temperature, and brine (15 mL) was added. The product was extracted with EtOAc (3 × 15 mL). The organic layer was washed with H_2_O, dried over anhydrous MgSO_4_, filtered, and concentrated.

*4-chloro-2-(1-naphthylsulfanyl)-5-sulfamoylbenzamide (***12f***, EA1A-NF).* The product was crystallized from toluene:MeOH (7:1).Yield: 277 mg, 70%, mp 277–280 °C.^1^H NMR δ ppm: 6.38 (1H, s, C_3_-H), 7.59 (2H, s, SO_2_NH_2_), 7.61–7.66 (2H, m, Naph-H), 7.67–7.71(1H, m, Naph-H), 7.85 (1H, s, CONH_2_), 8.00 (1H, dd, *J* = 7.2 Hz, *J* =1.2 Hz, Naph-H), 8.10 (1H, s, C_6_-H), 8.10–8.14 (2H, m, Naph-H), 8.21 (1H, d, *J* = 8.4 Hz, Naph-H), 8.34 (1H, s, CONH_2_). ^13^C NMR δ ppm: 125.2, 127.0, 127.5, 127.7, 128.5 128.6, 128.9, 129.7, 131.1, 132.1, 132.3, 133.7, 134.6, 135.0, 136.6, 137.5, 168.0. HRMS calculated for C_17_H_13_ClN_2_O_3_S_2_ [(M+H)^+^]: 393.0129, found: 393.0133.

*4-chloro-2-phenylsulfanyl-5-sulfamoylbenzamide (***12a***, EA1A-2)* [9].

*4-chloro-2-cyclohexylsulfanyl-5-sulfamoylbenzamide (***12b***, EA1A-3)* [9].

*2-benzylsulfanyl-4-chloro-5-sulfamoylbenzamide (***12c***, EA1A-4)* [9].

*4-chloro-2-(2-phenylethylsulfanyl)-5-sulfamoylbenzamide (***12d***, EA1A-5)* [9].

### 4.8. General Procedure for the Syntheses of ***5a***–***d***,***f***

The mixture of appropriate 2-substituted 4-chloro-5-sulfamoylbenzamide (compounds **12a**–**d,f**) (0.500 mmol), methanol (5 mL), and thionyl chloride (0.200 mL, 2.76 mmol) was heated at 50 °C temperature for 48–96 h. The progress of the reaction was monitored by TLC. The mixture was concentrated under reduced pressure.

*Methyl 4-chloro-2-phenylsulfanyl-5-sulfamoylbenzoate (***5a***, EA2-2).* The product was recrystallized from H_2_O. Yield: 122 mg, 68%, mp 223-226 °C.

*Methyl 4-chloro-2-cyclohexylsulfanyl-5-sulfamoylbenzoate (***5b***, EA1-3N). T*he product was recrystallized from H_2_O. Yield: 120 mg, 66%, mp 150–151 °C. ^1^H NMR δ ppm: 1.20–1.51 (5H, m, Cy-H), 1.58–1.66 (1H, m, Cy-H), 1.69–1.77 (2H, m, Cy-H), 1.93–2.02 (2H, m, Cy-H), 3.60–3.69 (1H, m, Cy-H), 3.86 (3H, s, CH_3_), 7,65 (1H, s, C_3_-H), 7.71 (2H, s, SO_2_NH_2_), 8.39 (1H, s, C_6_-H). ^13^C NMR δ ppm: 25.6, 25.7, 32.5, 42.9, 53.1, 126.3, 129.0, 131.6, 135.0, 136.9, 146.9, 165.0. HRMS calculated for C_14_H_18_ClNO_4_S_2_[(M+H)^+^]: 364.0439, found: 364.0444.

*Methyl 2-benzylsulfanyl-4-chloro-5-sulfamoylbenzoate (***5c***, EA1-4N).* The product was recrystallized from H_2_O. Yield: 145 mg, 78%, mp 211–212 °C. ^1^H NMR δ ppm: 3.86 (3H, s, CH_3_), 4.42 (2H, s, CH_2_), 7.30 (1H, t, *J =* 7.2 Hz, Ph-H), 7.37 (2H, t, *J =* 7.2 Hz, Ph-H), 7.47 (2H, d, *J =* 6.8 Hz, Ph-H) 7.70 (1H, s, C_3_-H), 7.73 (2H, s, SO_2_NH_2_), 8.44 (1H, s, C_6_-H). ^13^C NMR δ ppm: 36.0, 53.1, 124.7, 128.0, 128.5, 129.1, 129.7, 131.6, 135.2, 136.0, 136.8, 148.4, 164.9. HRMS calculated for C_15_H_14_ClNO_4_S_2_ [(M+H)^+^]: 372.0126, found: 372.0129.

*Methyl 4-chloro-2-(2-phenylethylsulfanyl)-5-sulfamoylbenzoate (***5d***, EA1-5N).* The product was recrystallized from H_2_O:acetone (3:1). Yield: 166 mg, 85%, mp 135–137 °C. ^1^H NMR δ ppm: 2.96 (2H, t, *J =* 7.6 Hz, CH_2_Ph), 3.39 (2H, t, *J =* 7.6 Hz, CH_2_S), 3.87 (3H, s, CH_3_), 7.24 (1H, m, Ph-H), 7.29–7.33 (4H, m, Ph-H), 7.63 (1H, s, C_3_-H), 7.73 (2H, s, SO_2_NH_2_), 8.44 (1H, s, C_6_-H). ^13^C NMR δ ppm: 33.7, 33.8, 53.1, 125.1, 126.9, 128.3, 128.9, 129.0, 131.6, 135.3, 136.7, 140.1, 148.5, 164.9. HRMS calculated for C_16_H_16_ClNO_4_S_2_ [(M+H)^+^]: 386.0282, found: 386.0286.

*Methyl 4-chloro-2-(1-naphthylsulfanyl)-5-sulfamoylbenzoate (***5f***, EA2-NF).* The product was recrystallized from H_2_O:MeOH (4:1). Yield: 143 mg, 70%, mp 235–237 °C.

### 4.9. Protein Preparation

Recombinant human carbonic anhydrases (CAs) were expressed in *E. coli* (CAI, CAII, mutant CAII^A65S, N67Q, I91L, F130V, V134L, L203A^, CAIII, CAIV, CAVA, CAVB, CAVII, CAXII, CAXIII, CAXIV), yeast (CAIX used to obtain only PDB ID: 7POM crystal structure) or mammalian cells (CAVI and CAIX used for all remaining experiments). Proteins were chromatographically purified following published protocols: CAI [22], CAII [23], CAII^A65S, N67Q, I91L, F130V, V134L, L203A^ (mimic-CAIX), CAIII, CAIV, CAVA, CAVB, CAVI, CAIX (except CAIX (PDB ID: 7POM) crystal was obtained using yeast-expressed protein [24], and [17], CAVII, CAXIII- [25], CAXII [26]. The protein stock solutions were stored at −80 °C. The protein concentration was determined by UV absorption at 280 nm using Beer-Lambert law and extinction coefficient determined by amino acid analysis. Proteins used for crystallization were additionally purified by affinity chromatography and concentrated.

### 4.10. Determination of Binding Parameters

#### 4.10.1. Fluorescent Thermal Shift Assay (FTSA)

*Observed* affinities (dissociation constant, *K_d,obs_*, proportional to the change in standard Gibbs energy upon binding, Δ*G* = *RT*ln*K_d_*) of the compounds binding to CAs were determined by the fluorescent thermal shift assay [27,28], using a QIAGEN’s real-time PCR cycler the “Rotor-Gene Q” and Rotor-Gene^®^ Style 4-strip tubes from STARLAB. Ligands were dissolved in DMSO stock solutions to concentrations of 10 mM or 20 mM and used for the serial dilution in DMSO of the dilution factor 2. These samples were diluted with buffer solution and mixed with a prepared protein solution, consisting of protein stock, buffer solution, and 8-anilino-1-naphthalene sulfonate (ANS; solvatochromic dye). All final samples typically contained up to 10 μM CA, compound solutions of serial dilution from 0 μM to 200 μM at 8 different concentrations differing by two times, 50 μM ANS, 50 mM sodium phosphate buffer at pH 7.0, 100 mM sodium chloride, and 2.0% (*v/v*) DMSO. Samples preparation is explained in detail in [28]. Protein denaturation was monitored by determining the fluorescence of ANS as a function of temperature[29]. The excitation and emission wavelengths of ANS are 365 ± 20 and 460 ± 15 nm. Samples were heated from 25 °C to 99 °C at the rate of 1 °C/min. The curve-fitting procedure has been explained previously [30] and was performed at 37 °C.

The dissociation constants of each compound with all 12 catalytically active human CAs are listed in Table 2, and some experimental data are provided in Supplementary material in Figures S1 and S2.

#### 4.10.2. Isothermal Titration Calorimetry (ITC)

Additionally, a selected set of compounds binding to CAs were tested by ITC [31,32] to determine changes in binding enthalpies. ITC experiments were carried out using a MicroCal PEAQ-ITC calorimeter (Northampton, MA, USA). The protein solution in the cell contained a constant concentration of CA (10–20 μM), while the concentration of compound loaded in the ITC syringe was ten times higher than the protein concentration (100–200 μM). Both cell and syringe solutions were diluted in the buffer: 50 mM sodium phosphate at pH 7.0 and containing 100 mM sodium chloride and 2.0 % (*v/v*) of DMSO. Samples were centrifuged prior to the experiment to improve quality. A typical experiment consisted of 19 injections with 150 s spacing between injections; the volume of the first injection was 0.4 μL (duration 0.8s) and 2.0 μL (duration 4.0 s) for the remaining injections. All experiments were performed at 37 °C with reference power 4.0 μcal/s.

The enthalpy changes of binding are listed in Table S8, and experimental data are provided in Supplementary material in Figure S6. A comparison of the affinity determined by FTSA and ITC is shown in Supplementary material in Figure S9. ITC shows weaker affinity than FTSA for strong interactions. Thus, the affinity determined by the FTSA was used due to the ITC detection limit as the more reliable.

#### 4.10.3. Calculation of Intrinsic Binding Parameters

All experiments allow the determination of the *observed* binding parameters of the sulfonamide binding to CA. However, the change in standard Gibbs energy (or dissociation constant), enthalpy, and entropy upon binding depend on buffer and pH because sulfonamide binding to CAs is linked to several reactions: (i) Zn^2+^-bound hydroxide ion protonate into water molecule in the active site of CA; (ii) Sulfonamide amino group (R-SO_2_NH_2_) deprotonate (R-SO_2_NH^-^); (iii) Buffer molecule protonate or deprotonate depending on the protons’ balance; (iv) After these reactions, the negatively charged sulfonamide (R-SO_2_NH^-^) replaces the Zn^2+^-bound water molecule in the active site of CA and binds to the protein in its position [15]. The sum of these reactions is the *observed* binding. However, only the fourth reaction represents the actual binding reaction, called *intrinsic* [16]. Observed binding parameters are important to obtain the compound’s affinity under certain conditions, for example, in the human body. Nevertheless, these parameters are less important in the drug design to estimate the energy for the binding affinity of each substituent. However, the contribution of protonation reactions can be subtracted. Equations of intrinsic dissociation constant (*K_d_intr_*) or standard Gibbs energy (Δ*G_intr_*) and enthalpy change (Δ*H_intr_*) are listed in Table 2 and Table S7 in Supplementary materials.

### 4.11. Determination of Protonation Parameters

#### 4.11.1. Determination of p*K_a_* Values

The p*K_a_* values of water molecules bound to Zn^2+^ in the active site of CAs, p*K_a_CAZn(II)H2O_*, were taken from [33] and of compounds, p*K_a_RSO2NH2_*, were determined as described in [34]. Figure 2D shows the intrinsic parameters of the binding of mimic-CAIX (mutant CAII^A65S, N67Q, I91L, F130V, V134L, L203A^) and compound **3b**, which were calculated using the p*K_a_CAZn(II)H2O_* of CAII.

We used a constant concentration of sulfonamide (25–400 μM) and 2.0% (*v/v*) of DMSO in universal buffer (50 mM sodium acetate, 25 mM sodium borate, and 50 mM sodium phosphate) at different pH values (in the range from pH 6 to 12 at every half pH unit). UV–VIS spectra of compound solution were recorded at 37 °C using the spectrophotometer “Agilent 89090A”. To determine p*K_a_RSO2NH2_*, a plot of the normalized ratio of two absorbancies (approximately 10 nm above and 10 nm below the isosbestic point) vs. buffer pH and fitted Henderson–Hasselbach curve using the least-square method. The midpoint of this fitted curve is equal to p*K_a_RSO2NH2_*. Experimental data are provided in Supplementary material in Figure S5 and Table 2.

#### 4.11.2. Determination of Protonation Enthalpy

The enthalpy changes of sulfonamide amino group (RSO_2_NH_2_) protonation, Δ*_p_RSO2NH2_H*, were determined by titration of 0.25 mM sulfonamide and 0.375 mM sodium hydroxide with 2.75 mM nitric acid using a MicroCal PEAQ-ITC calorimeter (Northampton, MA, USA). DMSO concentrations in the syringe and the sample cell were 2% (*v/v*). Experimental parameters: total number of injections was 40, the volume of each injection was 0.9 μL, spacing between injections was 120 s, the temperature was 37 °C. Δ*_p_CAZn(II)H2O_H* values of the water molecule in the active site CAs were taken from [33]. Figure 2D shows the change in binding enthalpy of mimic-CAIX (mutant CAII^A65S, N67Q, I91L, F130V, V134L, L203A^) and compound **3b**, which was calculated using the Δ*_p_CAZn(II)H2O_H* of CAII. Experimental data are provided in Supplementary material in Figure S7 and Table S5.

### 4.12. X-ray Crystallography: Crystallization, Data Collection, and Structure Determination

The proteins were ultrafiltered to the indicated concentration in Table S4. The same table lists the crystallization conditions and solutions. Crystals of CAIX (PDB ID: 7POM) and CAXII (PDB ID: 7PP9) were achieved by co-crystallization and others by soaking. The crystal soaking ligand solutions were produced by combining 50 μL of matching reservoir solution with 1 μL of 50 mM ligand solution in DMSO. The data were processed and scaled using XDS [35], MOSFLM [36], and SCALA [37]. Except CAXII dataset was processed and scaled using SAINT [38] and SADABS [39]. MOLREP [40] was used for molecular replacement with an initial model of 1CAB for CAI, 4HT0 for CAII, 3HLJ for mimic-CAIX, 6FE2 for CAIX, and 6QNL for CAXII. The model was refined with REFMAC [41] and fitted in the electron density map using COOT [42]. The 3D models of compounds were constructed by AVOGADRO [43] program, and ligand parameter files were created using LIBCHECK [44,45]. Coordinates and structure factors have been deposited to PDB. The PDB IDs, data collection, and refinement statistics are shown in Supplementary material in Table S3. The PyMOL program was used to create graphics.

## Figures and Tables

**Table 1:** The reaction of 2,4-dihalo-5-sulfamoyl-benzoates **1**, **2** with thiols and the ratios of the products as determined by NMR spectroscopy and HPLC/UV/MS system data (*italic*). * The separation of the 2- and 4-substituted isomers formed in the reaction of compounds **1** and **2** with phenylthiol using HPLC was unsuccessful.

Starting Compound	Thiol	Conversion (%)	2-Substituted (%)	4-Substituted (%)	2,4-Disubstituted (%)
**1**	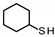	30.07 *28.77*	18.70 *17.38*	81.30 *82.62*	-
**2**	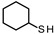	15.71 *13.92*	9.09 *14.11*	90.91 *85.89*	-
**1**	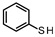	89.47 *96.67*	58.82	35.29	5.88 *11.22*
			88.78 *		
**2**	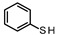	95.07 *96.92*	51.81	40.42	7.77 *14.45*
			85.55 *		
**1**	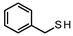	72.73 *71.55*	23.53 *22.91*	73.53 *72.79*	2.94 *4.30*
**1**	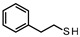	72.15 *73.60*	12.28 *12.05*	87.72 *87.95*	-
**1**	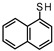	90.97 *95.25*	76.34 *78.84*	19.08 *15.72*	4.58 *5.44*
**1**	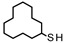	13.70 *18.19*	-	100.00 *100.00*	-

**Table 2:** *Intrinsic* (*K_d_intr_*), calculated according to the model, and *observed* (*K_d_obs_* at pH = 7.0), determined by the fluorescent thermal shift assay, dissociation constants, *K_d_* (nM), of investigated compounds binding to all catalytically active human carbonic anhydrases (CAs) at 37 °C. p*K_a_* values of the sulfonamide group were calculated from spectra at various pH as described in Methods and are listed to all compounds. Some compounds exhibited poorer solubility, which was observed visually and prompted in absorption spectra. Therefore, for such compounds, the p*K_a_* value was assigned based on the average value of the other compounds in that group and is marked with *.

Compound	Chemical Structure	*ortho* Substituent	*para* Substituent	p*K_a_*	*K_d_* (nM)	CAI	CAII	CAIII	CAIV	CAVA	CAVB	CAVI	CAVII	C A IX	CAXII	CAXIII	CAXIV
(Lab. Name)																	
**1**	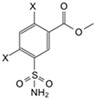	Cl	Cl	8.9	*Observed*	14,000	71	50,000	180	6700	310	170	67	59	560	83	72
(*EA2-1*)					*Intrinsic*	160	0.39	150	0.65	55	1.9	0.19	0.32	0.21	2.7	0.94	0.35
**2**		Br	Br	8.8	*Observed*	20,000	91	≥2 × 10^5^	140	3700	33	290	56	120	330	63	40
(*LJ14-4*)					*Intrinsic*	290	0.63	≥750	0.63	38	0.26	0.41	0.34	0.52	2	0.89	0.24
**3b**		*cyclohexyl*	Cl	9.6	*Observed*	17,000	250	40,000	400	100,000	11,000	330	150	0.33	33	50	13
(*EA2-3*)					*Intrinsic*	39	0.28	24	0.29	170	14	0.080	0.15	0.00020	0.030	0.11	0.010
**3c**	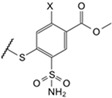	*benzyl*	Cl	9.5	*Observed*	6900	110	80,000	120	≥2 × 10^5^	5000	360	150	2.9	22	38	40
(*EA2-4*)					*Intrinsic*	20	0.16	61	0.11	≥420	7.9	0.10	0.19	0.0030	0.030	0.11	0.050
**3d**		*2-phenylethyl*	Cl	9.6	*Observed*	13,000	170	15,000	430	110,000	10,000	630	100	13	53	38	43
(*EA2-5*)					*Intrinsic*	30	0.18	9.3	0.30	190	13	0.14	0.10	0.0090	0.050	0.090	0.040
**3e**		*cyclododecyl*	Cl		*Observed*	12,000	400	≥2 × 10^5^	200	≥2 × 10^5^	67,000	2000	3300	0.44	41	27	37
(*EA2-12*)					*Intrinsic*	27	0.44	≥120	0.14	≥330	84	0.46	3.2	0.00030	0.040	0.060	0.040
**4b**		*cyclohexyl*	Br	9.6 *	*Observed*	6700	200	48,000	330	≥2 × 10^5^	3300	560	220	0.12	16	33	20
(*LJ15-12*)					*Intrinsic*	15	0.22	29	0.24	≥330	4.2	0.13	0.22	0.000080	0.020	0.080	0.020
**5a**	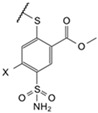	Cl	*phenyl*	9.4 * LS	*Observed*	3300	36	17,000	14	≥2 × 10^5^	250	200	50	2.5	5.7	20	19
(*EA2-2*)					*Intrinsic*	12	0.060	16	0.020	≥530	0.50	0.070	0.080	0.0030	0.0090	0.070	0.030
**5b**		Cl	*cyclohexyl*	9.4	*Observed*	330	20	≥2 × 10^5^	67	4000	5.7	210	21	7.5	13	10	5.1
(*EA1-3N*)					*Intrinsic*	1.2	0.040	≥190	0.080	11	0.010	0.070	0.030	0.0080	0.020	0.040	0.0080
**5c**		Cl	*benzyl*	9.4 * LS	*Observed*	2000	33	≥2 × 10^5^	120	1800	470	290	130	14	400	100	47
(*EA1-4N*)					*Intrinsic*	7.3	0.060	≥190	0.13	4.8	0.92	0.10	0.19	0.020	0.61	0.36	0.070
**5d**		Cl	*2-phenylethyl*	9.3	*Observed*	3400	27	89,000	1000	3800	4.4	850	24	12	180	7.4	17
(EA1-5N)					*Intrinsic*	16	0.060	110	1.5	13	0.010	0.38	0.050	0.020	0.34	0.030	0.030
**5f**		Cl	1-naphthyl	9.4 * LS	*Observed*	27,000	110	≥2 × 10^5^	66,000	96,000	510	14,000	1100	22	66	57	63,000
(EA2-NF)					*Intrinsic*	98	0.20	≥190	75	250	1.0	5.0	1.7	0.020	0.10	0.21	97
**6a**		Br	phenyl	9.4 * LS	*Observed*	2500	80	≥2 × 10^5^	40	86,000	31	420	210	9.8	20	16	13
(LJ15-11)					*Intrinsic*	9.2	0.14	≥190	0.050	230	0.060	0.15	0.32	0.010	0.030	0.060	0.020
**7b**	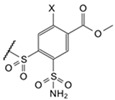	cyclohexyl	Cl	9.8	*Observed*	100,000	50,000	100,000	41,000	100,000	100,000	190,000	≥2 × 10^5^	9200	67,000	69,000	69,000
(**EA2-3o**)					*Intrinsic*	150	35	38	19	110	79	28	≥120	4.2	41	99	42
**7c**		benzyl	Cl	9.8* LS	*Observed*	≥2 × 10^5^	190,000	≥2 × 10^5^	5900	≥2 × 10^5^	≥2 × 10^5^	130,000	≥2 × 10^5^	790	6700	140,000	6700
(**EA2-4o**)					*Intrinsic*	≥290	130	≥76	2.7	≥210	≥160	19	≥120	0.36	4.1	200	4.1
**7d**		2-phenylethyl	Cl	9.8 * LS	*Observed*	≥2 × 10^5^	≥2 × 10^5^	≥2 × 10^5^	≥2 × 10^5^	≥2 × 10^5^	≥2 × 10^5^	130,000	≥2 × 10^5^	6700	34,000	22,000	≥2 × 10^5^
(**EA2-5o**)					*Intrinsic*	≥300	≥140	≥76	≥90	≥210	≥160	19	≥120	3.0	21	32	≥120
**8b**		cyclohexyl	Br	9.9	*Observed*	≥2 × 10^5^	≥2 × 10^5^	≥2 × 10^5^	≥2 × 10^5^	≥2 × 10^5^	≥2 × 10^5^	≥2 × 10^5^	≥2 × 10^5^	6700	≥2 × 10^5^	71,000	≥2 × 10^5^
(**LJ15-12o**)					*Intrinsic*	≥230	≥110	≥60	≥16	≥170	≥130	≥23	≥97	2.4	≥97	82	≥97
**9a**	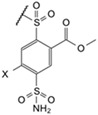	Cl	phenyl	8.2	*Observed*	140	0.91	3300	25	770	13	56	0.45	3.3	22	5.6	0.83
(EA2-2o)					*Intrinsic*	7.9	0.020	48	0.42	30	0.40	0.30	0.010	0.060	0.51	0.30	0.020
**9b**		Cl	cyclohexyl	8.2	*Observed*	67	0.67	2800	200	500	0.75	42	0.18	1	14	2.7	0.64
(EA1-3No)					*Intrinsic*	3.7	0.020	40	3.4	20	0.020	0.22	0.0040	0.020	0.33	0.15	0.010
**9c**		Cl	benzyl	8.2	*Observed*	560	5	450	100	830	1.1	14	2.5	2.5	20	5	2.6
(EA1-4No)					*Intrinsic*	31	0.13	6.5	1.7	33	0.030	0.080	0.060	0.040	0.46	0.27	0.060
**9d**		Cl	2-phenylethyl	8.4	*Observed*	330	1.7	920	140	200	1.1	26	0.85	2.5	48	3	1.3
(EA1-5No)					*Intrinsic*	12	0.030	8.5	1.6	5.1	0.020	0.090	0.010	0.030	0.72	0.10	0.020
**9f**		Cl	1-naphthyl	8.4	*Observed*	140	1.3	14,000	50	1400	3.4	100	0.67	10	50	1.3	4
(EA2-Nfo)					*Intrinsic*	5.1	0.020	130	0.55	36	0.060	0.35	0.010	0.11	0.74	0.040	0.060
**10a**		Br	phenyl	8.4	*Observed*	330	2.3	4800	370	1600	5.3	110	0.67	3.3	16	4.3	2
(LJ15-11o)					*Intrinsic*	12	0.040	44	4.0	41	0.10	0.37	0.010	0.040	0.24	0.15	0.030
**Acetazolamide**	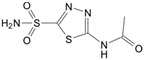			7.0	*Observed*	2400	46	40,000	87	840	140	220	13	21	130	120	63
					*Intrinsic*	1100	9.8	4600	12	270	34	9.8	2.4	2.9	24	53	12

Not determined, ND; low solubility, LS. * p*K_a_* value was assigned based on the average value of the other compounds in that group. The measurement limit is ≥2 × 10^5^. The highest used ligand concentration was 200 μM. *Intrinsic* values are calculated according to the model described in the Methods section.

## Data Availability

The data that support the findings of this study are available from the corresponding author upon reasonable request.

## Supplementary Materials

The following are available online at https://www.mdpi.com/article/10.3390/ijms23010130/s1.

## Abbreviations

CA	carbonic anhydrase
FTSA	fluorescent thermal shift assay (or differential scanning fluorimetry, DSF)
intr	intrinsic
ITC	isothermal titration calorimetry
obs	observed
VARIABLES	
Δ*G_intr_*	change of the intrinsic standard Gibbs energy upon binding
Δ*G_obs_*	change of the observed standard Gibbs energy upon binding
Δ*H_intr_*	change of the intrinsic standard enthalpy upon binding
Δ*H_obs_*	change of the observed standard enthalpy upon binding
*K_d_intr_*	the intrinsic equilibrium dissociation constant
*K_d_obs_*	the observed equilibrium dissociation constant
*T_m_*	melting temperature (midpoint of the unfolding transition)
*T*Δ*S_intr_*	change of the intrinsic standard entropy upon binding multiplied by absolute temperature
