# Supplementary material for: Methyl 2-Halo-4-Substituted-5-Sulfamoyl-Benzoates as High Affinity and Selective Inhibitors of Carbonic Anhydrase IX
Source: Int J Mol Sci. 2021 Dec 23;23(1):130. doi: 10.3390/ijms23010130 (PMC8745178; doi:10.3390/ijms23010130)
Supplement: Supplementary file 1 [file ijms-23-00130-s001.zip › ijms-1501810-supplementary.pdf]

Supplementary material for the manuscript:

Methyl 2-halo-4-substituted-5-sulfamoyl-benzoates as high affinity and selective inhibitors of carbonic anhydrase IX

Audrius Zakšauskas[a], Edita Čapkauskaitė[a], Vaida Paketurytė-Latvė[a], Alexey Smirnov[a], Janis Leitans[c], Andris Kazaks[c], Elviss Dvinskis[c], Laimonas Stančaitis[a], Aurelija Mickevičiūtė[a], Jelena Jachno[a], Linas Jezepčikas[a], Vaida Linkuvienė[a], Andrius Sakalauskas[a], Elena Manakova[b], Saulius Gražulis[b], Jurgita Matulienė[a], Kaspars Tars[c], and Daumantas Matulis[a,]*

[a] Department of Biothermodynamics and Drug Design, Institute of Biotechnology, Life Sciences Center, Vilnius University, Saulėtekio al. 7, Vilnius LT-10257, Lithuania.

[b] Department of Protein – DNA Interactions, Institute of Biotechnology, Life Sciences Center, Vilnius University, Saulėtekio al. 7, Vilnius LT-10257, Lithuania.

[c] Latvian Biomedical Research and Study Centre, Ratsupites 1 k-1, Riga LV-1067, Latvia.

* Corresponding author: Daumantas Matulis

Address:

Saulėtekio al. 7, Vilnius LT-10257, Lithuania.

Tel. +370-5-223-4364

Fax.+370-5-223-4367

Email: daumantas.matulis@bti.vu.lt

Table of Contents

Table of Contents 2

FTSA data of compound binding to CA isoenzymes 3

X-ray crystallography 9

Determination of pK_a values of inhibitor –RSO_2NH_2 15

Standard enthalpy change upon protonation of inhibitor –RSO_2NH_2 19

ITC data of selected compound binding to CA isoenzymes 21

Equations for calculation of K_{d_intr}, ΔG_{intr} and ΔH_{intr} 34

Determination of the regio selectivity of the nucleophilic aromatic substitution of a halogen in methyl 2,4-dihalo-5-sulfamoyl-benzoates 1, 2 with thiols 35

NMR spectra of synthesized compounds 37

References 58

FTSA data of compound binding to CA isoenzymes

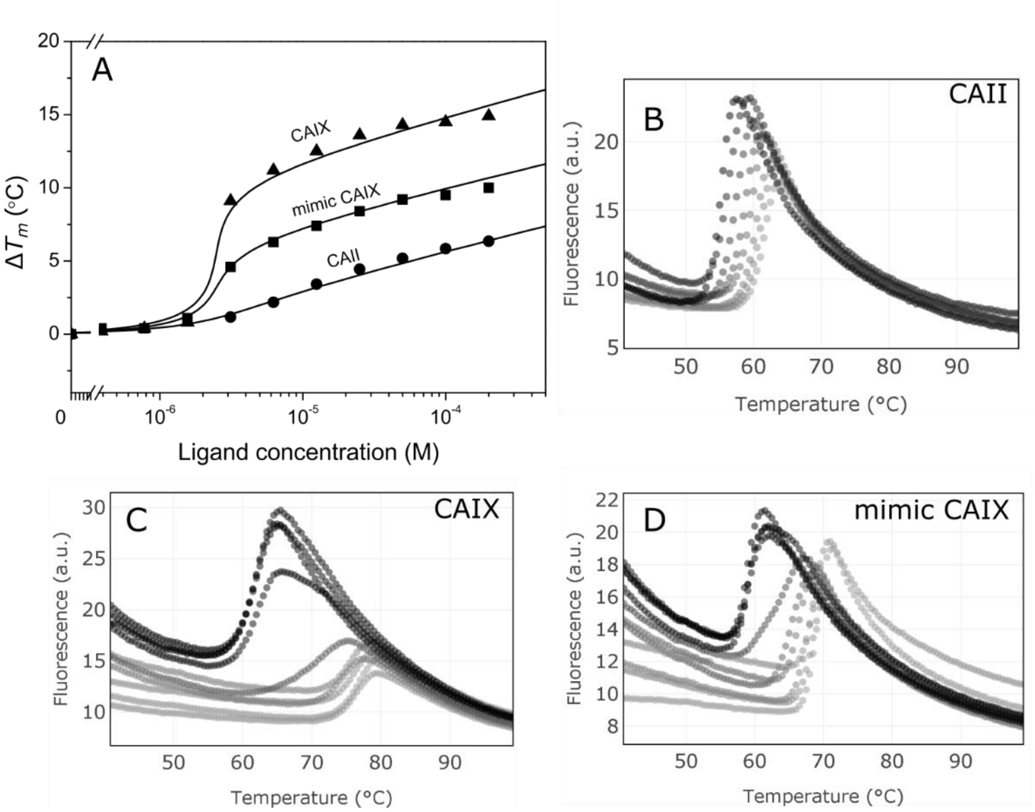

Figure S1. Determination of 3b (*EA2-3*) affinity for recombinant human CAII, CAIX and mimic CAIX (mutant of CAII) by the fluorescent thermal shift assay (FTSA). A. The dosing curves of compound **3b** (*EA2-3*) - the melting temperatures plotted as a function of added compound concentration (data points) and fitted to a model for 37 °C (solid lines). **B**, **C**, and **D**. The protein unfolding curves recorded by following the fluorescence of 8-anilino-1-naphthalene sulfonate at increasing compound concentrations from 0 to 200 µM, from black to gray.

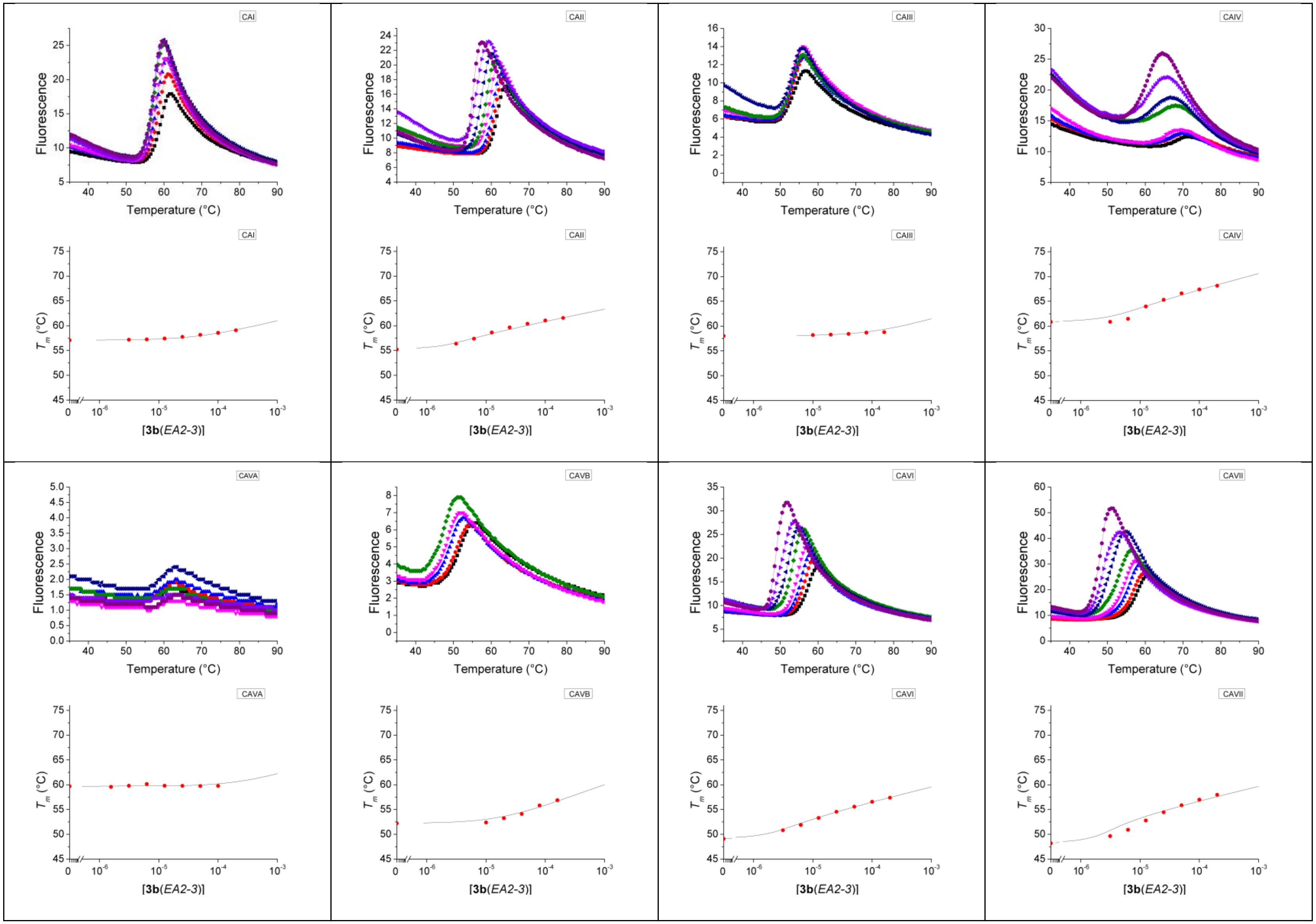
CAI
CAII
CAIII
CAIV
CAVA
CAVB
CAVI
CAVII
Fluorescence
Temperature (°C)
T_m (°C)
[3b(EA2-3)]

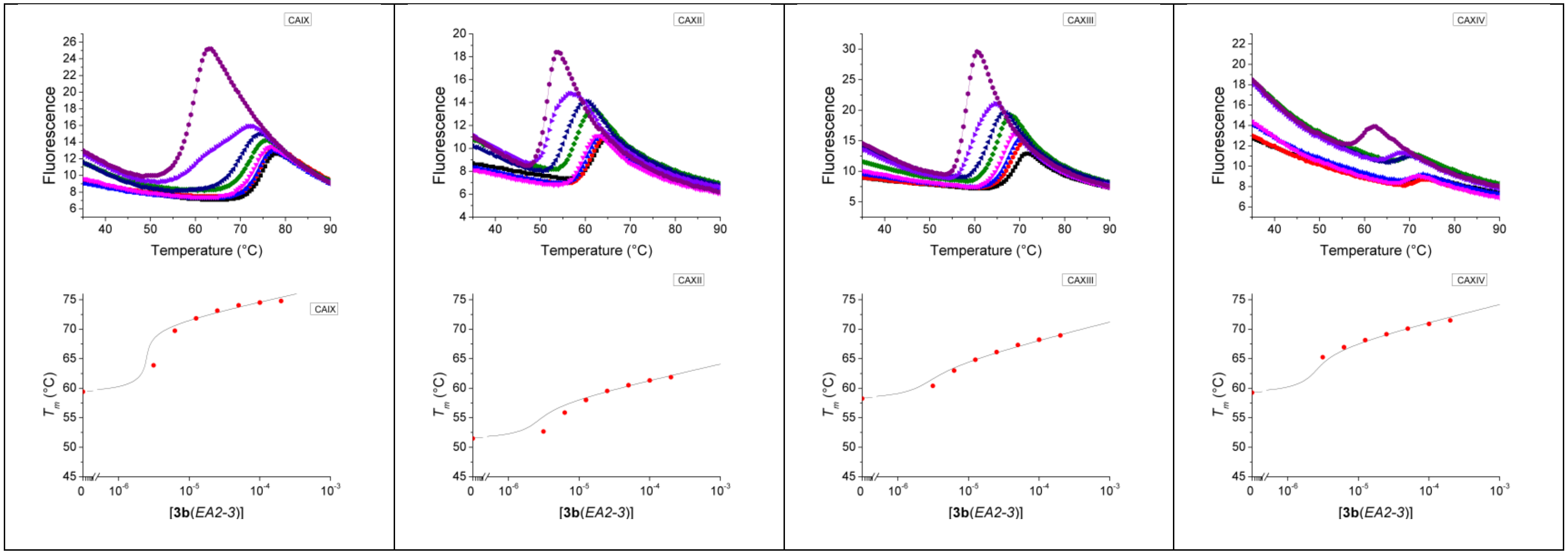

Figure S2. Determination of compound binding to human CA by the fluorescent thermal shift assay (FTSA). (Upper panels) Thermal denaturation curves of CA in the presence of increasing concentrations of compound **3b** (*EA2-3*). (Lower panels) The melting temperatures plotted as a function of added compound concentration (data points), and fitted to a model for 37 °C (solid lines).

Table S1. *Observed* standard Gibbs energy changes, ΔG_{obs} (kJ/mol), upon compound binding to CA isoenzymes. Values were converted from K_{d_obs} in Table 1 (at 37 °C, pH 7.0 by FTSA). Green are the weakest, red - the strongest interactions. Limit of the weakest compound determination is -22.0 kJ/mol, corresponding to K_{d_obs} $2 \cdot 10^5$ nM. There is no distinct limit for high affinity determination in FTSA.

Compound	*Lab. Name*	CAI	CAII	CAIII	CAIV	CAVA	CAVB	CAVI	CAVII	CAIX	CAXII	CAXIII	CAXIV
**1**	*EA2-1*	-28.8	-42.4	-25.5	-40.0	-30.7	-38.6	-40.2	-42.6	-42.9	-37.1	-42.0	-42.4
**2**	*LJ14-4*	-27.9	-41.8	≥-22.0	-40.6	-32.2	-44.4	-38.9	-43.1	-41.2	-38.5	-42.8	-43.9
**3b**	*EA2-3*	-28.4	-39.2	-26.1	-38.0	-23.7	-29.4	-38.5	-40.5	-56.3	-44.4	-43.3	-46.9
**3c**	*EA2-4*	-30.6	-41.3	-24.3	-41.1	≥-22.0	-31.5	-38.3	-40.5	-50.7	-45.4	-44.0	-43.9
**3d**	*EA2-5*	-29.0	-40.2	-28.6	-37.8	-23.4	-29.7	-36.8	-41.6	-46.9	-43.2	-44.1	-43.7
**3e**	*EA2-12*	-29.3	-38.0	≥-22.0	-39.8	≥-22.0	-24.8	-33.8	-32.5	-55.5	-43.9	-45.0	-44.1
**4b**	*LJ15-12*	-30.7	-39.8	-25.7	-38.5	≥-22.0	-32.5	-37.1	-39.5	-59.0	-46.3	-44.4	-45.7
**5a**	*EA2-2*	-32.5	-44.2	-28.4	-46.6	≥-22.0	-39.2	-39.8	-43.3	-51.1	-48.9	-45.7	-45.8
**5b**	*EA1-3N*	-38.5	-45.7	≥-22.0	-42.6	-32.0	-48.9	-39.7	-45.6	-48.2	-46.8	-47.5	-49.2
**5c**	*EA1-4N*	-33.8	-44.4	≥-22.0	-41.1	-34.1	-37.6	-38.9	-41.0	-46.6	-38.0	-41.6	-43.5
**5d**	*EA1-5N*	-32.4	-44.9	-24.0	-35.5	-32.2	-49.6	-36.1	-45.3	-47.0	-40.1	-48.3	-46.1
**5f**	*EA2-NF*	-27.2	-41.3	≥-22.0	-24.8	-23.9	-37.3	-28.8	-35.4	-45.5	-42.6	-43.0	-24.9
**6a**	*LJ15-11*	-33.3	-42.1	≥-22.0	-43.9	-24.1	-44.6	-37.9	-39.6	-47.5	-45.8	-46.3	-46.8
**7b**	*EA2-3o*	-23.7	-25.5	-23.7	-26.0	-23.7	-23.7	-22.1	≥-22.0	-29.9	-24.8	-24.7	-24.7
**7c**	*EA2-4o*	≥-22.0	≥-22.1	≥-22.0	-31.1	≥-22.0	≥-22.0	-23.0	≥-22.0	-36.2	-30.7	-22.9	-30.7
**7d**	*EA2-5o*	-21.9	≥-22.0	≥-22.0	≥-22.0	≥-22.0	≥-22.0	-23.0	≥-22.0	-30.7	-26.5	-27.6	≥-22.0
**8b**	*LJ15-12o*	≥-22.0	≥-22.0	≥-22.0	-25.8	≥-22.0	≥-22.0	≥-22.0	≥-22.0	-30.7	≥-22.0	-24.6	≥-22.0
**9a**	*EA2-2o*	-40.6	-53.7	-32.5	-45.1	-36.3	-46.8	-43.1	-55.5	-50.3	-45.4	-49.0	-53.9
**9b**	*EA1-3No*	-42.6	-54.5	-32.9	-39.8	-37.4	-54.2	-43.8	-57.9	-53.4	-46.5	-50.8	-54.6
**9c**	*EA1-4No*	-37.1	-49.3	-37.7	-41.6	-36.1	-53.2	-46.6	-51.1	-51.1	-45.7	-49.3	-51.0
**9d**	*EA1-5No*	-38.5	-52.1	-35.8	-40.6	-39.8	-53.2	-45.0	-53.8	-51.1	-43.4	-50.6	-52.8
**9f**	*EA2-Nfo*	-40.6	-52.9	-28.8	-43.3	-34.7	-50.3	-41.6	-54.5	-47.5	-43.4	-52.9	-49.9
**10a**	*LJ15-11o*	-38.5	-51.3	-31.6	-38.2	-34.4	-49.2	-41.4	-54.5	-50.3	-46.3	-49.6	-51.6

Table S2. ***Intrinsic*** **standard Gibbs energy changes, ΔG_{intr} (kJ/mol), upon compound binding to CA isoenzymes, converted from K_{d_intr} in Table 1** (at 37 °C and it is independent of pH). Green are the weakest, while red - the strongest interactions. The detection limit of ΔG_{obs} = -22.0 kJ/mol has been recalculated for the corresponding CA - compound pair and differs due to pK_a. The table also shows the pK_a values (p$K_{a\ RSO_2NH_2}$ - determined in this study, p$K_{a\ CA\text{-}Zn(II)H_2O}$ – taken from (Linkuvienė et al. 2018)) and fractions at pH 7.0 of deprotonated sulfonamide, $f_{RSO_2NH^-}$, and CA-Zn(II)-bound water molecule, $f_{CA\text{-}Zn(II)H_2O}$, used to calculate the intrinsic affinity (see the equations S1-S4 in the Supplementary Material).

				**CAI**	**CAII**	**CAIII**	**CAIV**	**CAVA**	**CAVB**	**CAVI**	**CAVII**	**CAIX**	**CAXII**	**CAXIII**	**CAXIV**
			p$K_{a\ CA\text{-}Zn(II)H_2O}$	8.1	6.9	6.5	6.6	7.3	7.0	6	6.8	6.6	6.8	8.0	6.8
**Compound**	*Lab. name*	p$K_{a\ RSO_2NH_2}$	$f_{CA\text{-}Zn(II)H_2O}$ / $f_{RSO_2NH^-}$	92.6%	44.3%	24.0%	28.5%	66.6%	50.0%	9.1%	38.7%	28.5%	38.7%	90.9%	38.7%
**1**	*EA2-1*	8.9	1.2%	-40.3	-55.8	-40.5	-54.6	-43.1	-51.7	-57.7	-56.4	-57.5	-50.9	-53.6	-56.2
**2**	*LJ14-4*	8.8	1.6%	-38.8	-54.6	≥-36.4	-54.6	-44.0	-56.9	-55.8	-56.3	-55.1	-51.6	-53.7	-57.1
**3b**	*EA2-3*	9.6	0.3%	-44.0	-56.7	-45.2	-56.7	-40.2	-46.6	-60.1	-58.3	-75.0	-62.3	-59.0	-64.8
**3c**	*EA2-4*	9.5	0.3%	-45.7	-58.2	-42.9	-59.2	≥-37.9	-48.1	-59.3	-57.8	-68.8	-62.7	-59.1	-61.2
**3d**	*EA2-5*	9.6	0.3%	-44.7	-57.8	-47.7	-56.5	-39.9	-46.9	-58.5	-59.5	-65.6	-61.1	-59.8	-61.6
**3e**	*EA2-12*	9.6	0.3%	-44.9	-55.5	≥-41.1	-58.5	≥-38.5	-42.0	-55.5	-50.4	-74.2	-61.8	-60.7	-62.0
**4b**	*LJ15-12*	9.6	0.3%	-46.4	-57.3	-44.8	-57.1	≥-38.5	-49.8	-58.8	-57.4	-77.6	-64.2	-60.1	-63.6
**5a**	*EA2-2*	9.4	0.4%	-47.0	-60.6	-46.3	-64.1	≥-37.3	-55.2	-60.2	-60.1	-68.6	-65.7	-60.2	-62.5
**5b**	*EA1-3N*	9.4	0.4%	-52.9	-62.1	≥-39.9	-60.1	-47.4	-65.0	-60.2	-62.3	-65.7	-63.5	-62.0	-65.9
**5c**	*EA1-4N*	9.4	0.4%	-48.3	-60.8	≥-39.9	-58.6	-49.4	-53.6	-59.3	-57.7	-64.1	-54.7	-56.1	-60.2
**5d**	*EA1-5N*	9.3	0.5%	-46.3	-60.7	-41.4	-52.4	-46.9	-65.1	-55.9	-61.4	-63.9	-56.2	-62.2	-62.2
**5f**	*EA2-NF*	9.4	0.4%	-41.6	-57.7	≥-39.9	-42.3	-39.2	-53.4	-49.3	-52.1	-63.0	-59.4	-57.5	-41.6
**6a**	*LJ15-11*	9.4	0.4%	-47.7	-58.5	≥-39.9	-61.4	-39.5	-60.7	-58.3	-56.4	-65.0	-62.5	-60.8	-63.5
**7b**	*EA2-3o*	9.8	0.2%	-40.6	-44.3	-44.1	-45.9	-41.4	-42.2	-44.9	≥-41.0	-49.8	-43.9	-41.6	-43.8
**7c**	*EA2-4o*	9.8	0.2%	≥-38.8	≥-40.9	≥-42.3	-50.9	≥-39.6	≥-40.4	-45.8	≥-41.0	-56.1	-49.8	-39.8	-49.8
**7d**	*EA2-5o*	9.8	0.2%	-38.8	≥-40.7	≥-42.3	≥-41.8	≥-39.6	≥-40.4	-45.8	≥-41.0	-50.6	-45.6	-44.5	≥-41.0

**8b**	*LJ15-12o*	9.9	0.1%	≥-39.4	≥-41.3	≥-42.9	-46.2	≥-40.2	≥-41.0	≥-45.4	≥-41.6	-51.2	≥-41.6	-42.1	≥-41.6
**9a**	*EA2-2o*	8.2	5.9%	-48.1	-63.1	-43.5	-55.7	-44.6	-55.8	-56.5	-65.2	-60.9	-55.2	-56.5	-63.6
**9b**	*EA1-3No*	8.2	5.9%	-50.1	-63.9	-43.9	-50.3	-45.7	-63.2	-57.3	-67.6	-64.0	-56.3	-58.4	-64.3
**9c**	*EA1-4No*	8.2	5.9%	-44.6	-58.7	-48.6	-52.1	-44.4	-62.2	-60.0	-60.8	-61.6	-55.4	-56.8	-60.7
**9d**	*EA1-5No*	8.4	3.8%	-47.1	-62.6	-47.9	-52.3	-49.2	-63.4	-59.6	-64.7	-62.7	-54.3	-59.3	-63.7
**9f**	*EA2-Nfo*	8.4	3.8%	-49.3	-63.4	-40.8	-55.0	-44.2	-60.5	-56.2	-65.3	-59.2	-54.2	-61.5	-60.7
**10a**	*LJ15-11o*	8.4	3.8%	-47.1	-61.8	-43.7	-49.9	-43.9	-59.4	-56.0	-65.3	-62.0	-57.2	-58.3	-62.5
**EA3-2o***		8.3	5.3%	-41.6	-58.9	-41.3	-55.3	-44.3	-55.5	-49.6	-62.5	-61.2	-58.9	-54.0	-60.7
**EA3-3***		9.0	1.0%	-47.7	-61.2	-44.7	-62.5	-49.9	-55.9	-53.0	-65.6	-67.7	-65.9	-56.9	-67.7

*-Data taken from – (Zakšauskas et al. 2020).

X-ray crystallography

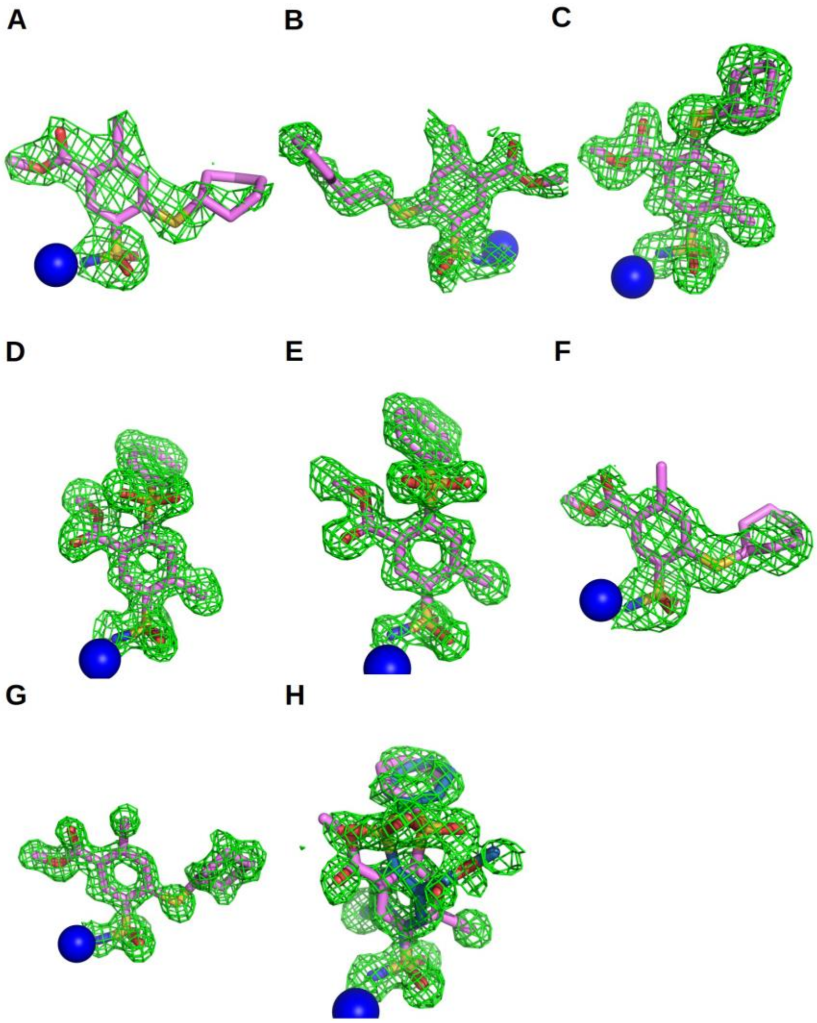

Figure S3. The electron densities |F(o)-F(c)| of the ligands calculated in the absence of ligand and contoured at 3σ. Zinc ion is shown as a blue sphere. **A**, Compound **3b** (*EA2-3*) in the active site of CAXII (PDB ID: 7PP9, A subunit). **B**, Compound **3d** (*EA2-5*) in the active site of CAXII (PDB ID: 7PUW). **C**, Compound **5b** (*EA1-3N*) in the active site of CAXII (PDB ID: 7PUU, A subunit). **D**, Compound **9a** (*EA2-2o*) in the active site of CAXII (PDB ID: 7PUV). **E**, Compound **9a** (*EA2-2o*) in the active site of CAII (PDB ID: 7Q0E, A subunit). **F**, Compound **3b** (*EA2-3*) in the active site of CAIX (PDB ID: 7POM, A subunit). **G**, Compound **3b** (*EA2-3*) in the active site of mimic-CAIX ($\text{CAII}^{\text{A65S, N67Q, I91L, F130V, V134L, L203A}}$) (PDB ID: 7Q0C, A subunit). **H**, Compound **9a** (*EA2-2o*) in the active site of CAI (PDB ID: 7Q0D, A subunit).

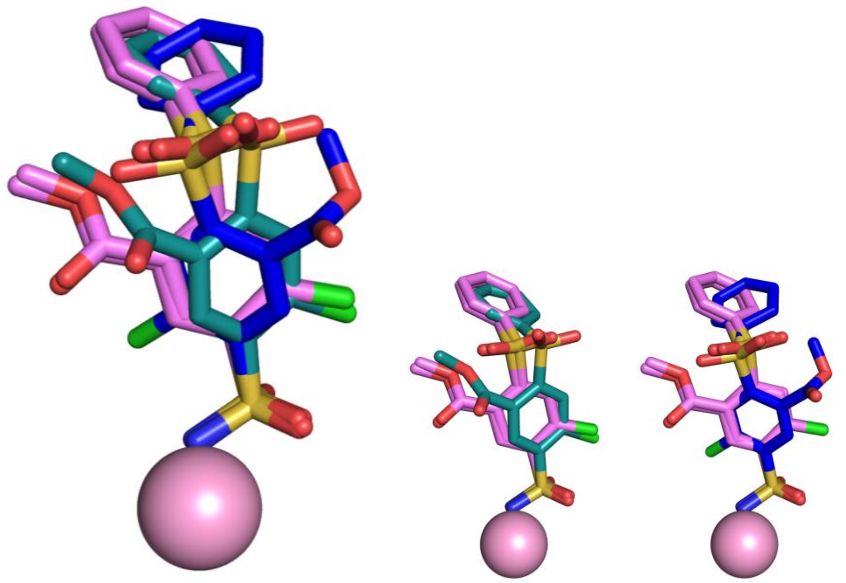

Figure S4. Compound **9a** (*EA2-2o*) in the active sites of CAI (PDB ID: 7Q0D, A subunit) – two alternatives in blue and teal color, CAII (PDB ID: 7Q0E, A subunit) and CAXII (PDB ID: 7PUV) – both in pink. Zinc ion is shown as a violet sphere. The figure on the left shows all 3 structures with both alternatives and other figures show separate alternatives of compound in the active site of CAI.

Table S3. X-ray crystallography data collection and refinement statistics of human CA complexes with inhibitors. All datasets were collected at 100 K, test set size was 10%.

Isoform-ligand	CAI – **9a**(*EA2-2o*)	CAII – **9a**(*EA2-2o*)	CAIX – **3b**(*EA2-3*)	mimic CAIX – **3b**(*EA2-3*)
PDB ID	7Q0D	7Q0E	7POM	7Q0C
Data-collection statistics				
Space group	$P2_12_12_1$	$P12_11$	H3	$P12_11$
Unit-cell parameters (Å)	a=62.0, b=73.2, c=120.5	a=42.2, b=41.4, c=72.0, β=104.3°	a=152.66, b=152.66, c=171.59	a=42.1, b=41.4, c=72.0, β=104.2°
Resolution range (Å)	1.2-55.2	1.3-39.8	1.98-35.98	1.1-35.6
Wavelength (Å)	1.03320	0.97970	0.91840	0.97552
Radiation source	EMBL, P13<br>2015-06-26	EMBL, P13<br>2016-07-19	BESSY II, 14.1	EMBL, P14<br>2013-05-21
Unique reflections number	151876	57251	101441	87185
R_{merge}, overall (outer shell)	0.075 (0.194)	0.086 (0.354)	0.081 (0.543)	0.057 (0.119)
I/σ overall (outer shell)	22.0 (9.6)	13.3 (5.5)	6.7 (2.0)	20.2 (9.3)
Multiplicity overall (outer shell)	12.9 (11.0)	6.9 (7.0)	2.7 (2.8)	6.4 (4.7)
Completeness (%) overall (outer shell)	98.3 (89.6)	96.8 (94.6)	97.0 (98.7)	95.2 (76.8)
Wilson B-factor	10.7	7.0	27.5	9.9
Refinement statistics:				
R_{work}	0.131	0.126	0.176	0.169
R_{free}	0.158	0.169	0.214	0.204
RMSD bond lengths, (Å)	0.030	0.025	0.010	0.026
RMSD	2.421	2.304	1.61	2.574

bond angles (°)				
Average B factors (Å^2):				
all	17.9	12.9	33.7	18.1
main-chain	12.9	9.3	33.8	14.3
side-chain	15.0	12.4	37.5	18.3
inhibitors	23.2	7.6	39.4	14.4
waters	32.0	25.1	43.1	30.8
zinc	7.9	3.6	25.1	6.5
other molecules	32.8	27.2		22.7
Number of atoms:				
all	5497	2602	8467	5068
protein	4394	2196	7634	4401
inhibitor	96	24	88	44
water	869	351	741	609
zinc	2	1	4	2
other molecules	136	30		12
Ramachandran statistics (%):				
most favored regions	96	95	96	95
additionally allowed regions	4	13	4	5
Outliers	0	0	0	0

Isoform-ligand	CAXII – **9a**(*EA2-2o*)	CAXII – **5b** (*EA1-3N*)	CAXII – **3d** (*EA2-5*)	CAXII – **3b** (*EA2-3*)
PDB ID	7PUV	7PUU	7PUW	7PP9
Data-collection statistics				
Space group	$P12_11$	$P12_11$	$P12_11$	P1
Unit-cell parameters (Å)	a=77.4, b=74.5, c=91.9, β=109.0°	a=77.3, b=74.1, c=91.6, β=108.8°	a=77.3, b=74.2, c=91.7, β=108.8°	a=45.8, b=74.9, c=76.4, β=105.5°
Resolution range (Å)	1.4-73.2	1.51-73.2	1.42-86.8	2.34-74.86
Wavelength (Å)	0.97970	0.97620	0.97620	1.5418

Radiation source	EMBL, P13<br>2016-07-19	EMBL, P13<br>2016-12-05	EMBL, P13<br>2016-12-05	Bruker AXS MICROSTAR
Unique reflections number	190273	145217	181236	54029
R_{merge}, overall (outer shell)	0.096 (0.237)	0.038 (0.168)	0.058 (0.261)	0.16 (0.35)
I/σ overall (outer shell)	13.6 (6.5)	25.2 (8.2)	16.5 (5.0)	9.6 (2.7)
Multiplicity overall (outer shell)	6.7 (5.5)	6.9 (6.3)	6.6 (5.5)	5.23 (3.58)
Completeness (%) overall (outer shell)	98.0 (87.0)	94.0 (73.2)	97.7 (85.9)	94.7 (98.3)
Wilson B-factor	12.8	15.6	15.1	14.3
Refinement statistics:				
R_{work}	0.159	0.166	0.174	0.161
R_{free}	0.217	0.199	0.202	0.248
RMSD bond lengths, (Å)	0.014	0.012	0.012	0.010
RMSD bond angles (°)	2.047	1.888	1.899	1.744
Average B factors ($Å^2$):				
all	18.5	20.2	20.3	17.05
main-chain	15.5	16.5	17.2	17.1
side-chain	19.1	19.6	20.7	19.3
inhibitors	14.5	18.0	34.6	23.3
waters	28.7	29.2	30.3	21.4
zinc	8.9	9.6	10.5	12.1
other molecules	27.6	28.3	26.8	
Number of atoms:				
all	9817	9752	9674	9142

protein	8539	8526	8487	8316
inhibitor	96	88	96	88
water	1143	1114	1071	734
zinc	4	4	4	4
other molecules	35	20	16	
Ramachandran statistics (%):				
most favored regions	98	97	98	96
additionally allowed regions	2	7	2	4
Outliers	2	0	0	0

Table S4. The crystallization conditions used to grow the crystals in this study. 7POM and 7PP9 were obtained by co-crystallization, while others by inhibitor soaking.

Isoform-ligand	Crystallization buffer	PDB ID	Sitting drop
CAI – **9a** (*EA2-2o*)	0.1 M Tris–HCl (pH 8.5), 0.2 M ammonium acetate and 24% PEG4000	7Q0D	0.9 µL of 16.9 mg mL^{-1} CAI protein solution and 0.5 µL of crystallization buffer
CAII – **9a** (*EA2-2o*)	0.1M sodium bicine (pH 9.0), 0.2 M ammonium sulfate and 2M sodium malonate (pH 7.0)	7Q0E	1.5 µL of 38 mg mL^{-1} CAII protein solution and 2.5 µL of crystallization buffer
CAIX – **3b** (*EA2-3*)	1.0M di-ammonium hydrogen phosphate, 0.1M sodium acetate pH 4.5	7POM	1.5 µL of 10 mg mL^{-1} CAIX protein solution, 1.5 µL of crystallization buffer and 0.1 µL of 100 mM inhibitor solution (100% DMSO)
mimic CAIX – **3b** (*EA2-3*)	0.1M sodium bicine (pH 9.0), 0.2 M ammonium sulfate and 2M sodium malonate (pH 7.0)	7Q0C	1 µL of 18.6 mg mL^{-1} mimic-CAIX protein solution and 1.5 µL of crystallization buffer
CAXII – **9a** (*EA2-2o*)	0.1M ammonium citrate (pH 7.0), 0.2 M ammonium sulfate and 30% PEG4000	7PUV	2.7 µL of 20 mg mL^{-1} CAXII protein solution and 2.7 µL of crystallization buffer
CAXII – **5b** (*EA1-3N*)	0.1M ammonium citrate (pH 7.0), 0.2 M ammonium sulfate and 30% PEG4000	7PUU	2.5 µL of 33.2 mg mL^{-1} CAXII protein solution and 2.5 µL of crystallization buffer
CAXII – **3d** (*EA2-5*)	0.1M ammonium citrate (pH 7.0), 0.2 M ammonium sulfate and 30% PEG4000	7PUW	2.5 µL of 33.2 mg mL^{-1} CAXII protein solution and 2.5 µL of crystallization buffer
CAXII – **3b** (*EA2-3*)	0.2M ammonium acetate, 0.1M sodium citrate pH 5.6, 31% PEG 4000	7PP9	1.5 µL of 10 mg mL^{-1} CAXII protein solution, 1.5 µL of crystallization buffer and 0.1 µL of 100 mM inhibitor solution (100% DMSO)

Determination of pK_a values of inhibitor –RSO_2NH_2

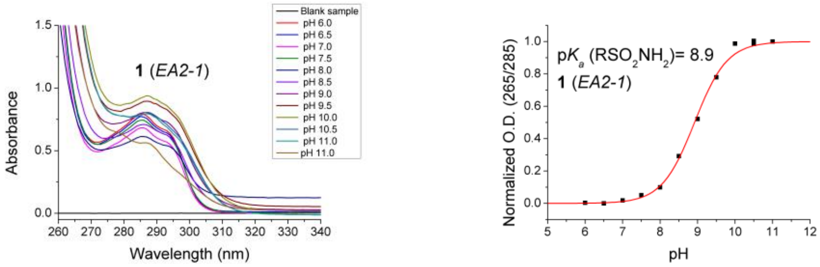

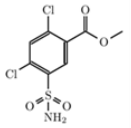

1
EA2-1

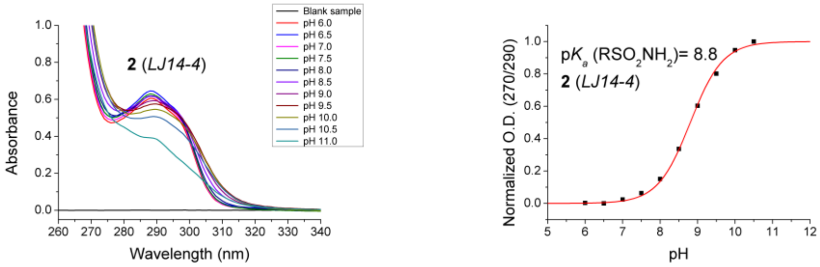

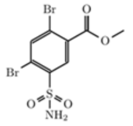

2
LJ14-4

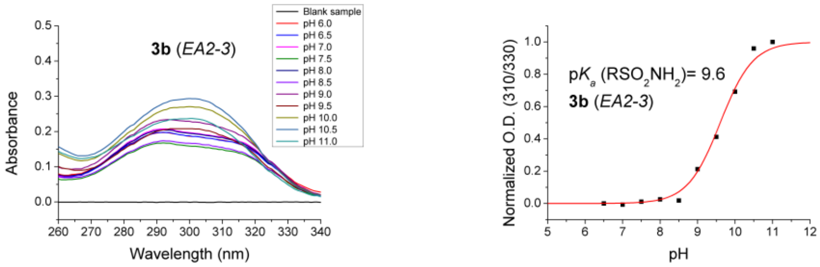

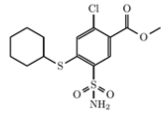

3b
EA2-3

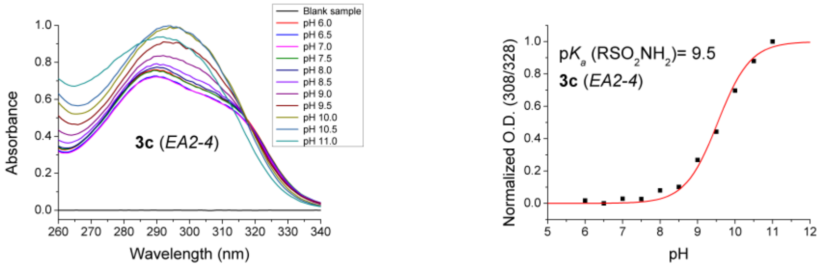

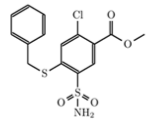

3c
EA2-4

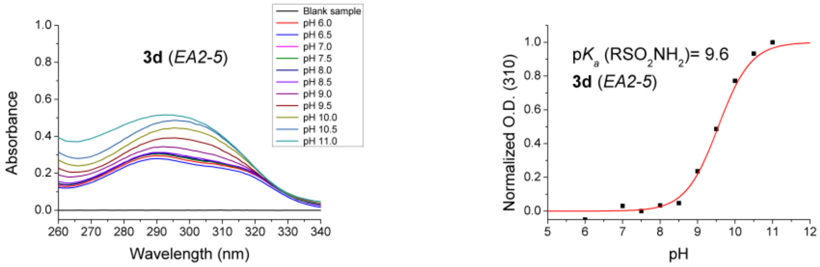

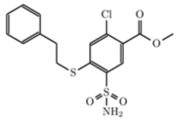

3d
EA2-5

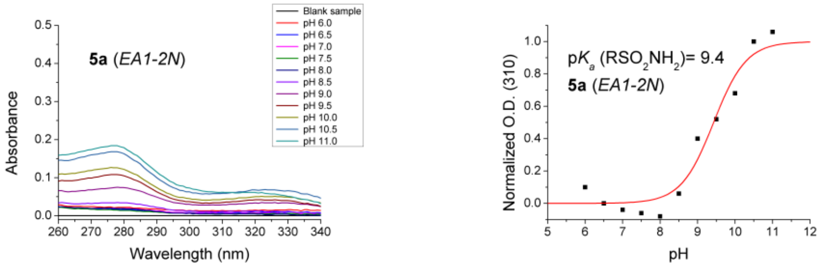

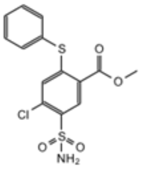

5a
EA 1-2N or EA2-2

Continued on next page...

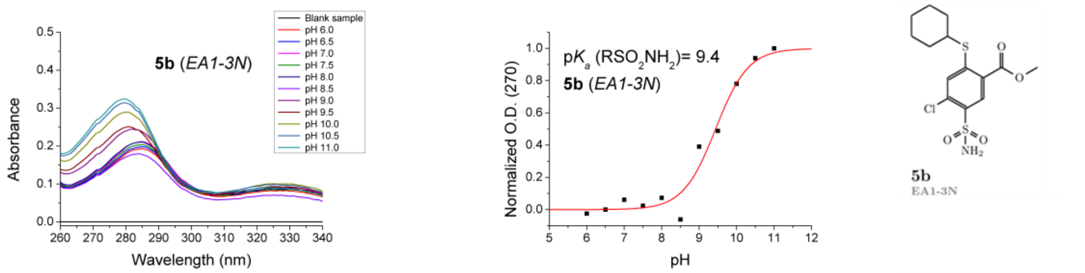

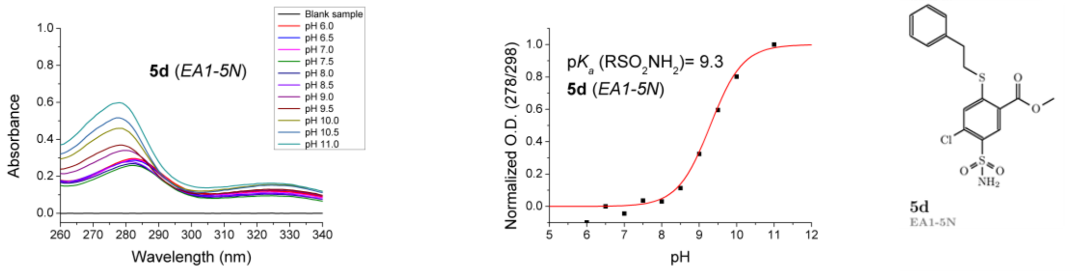

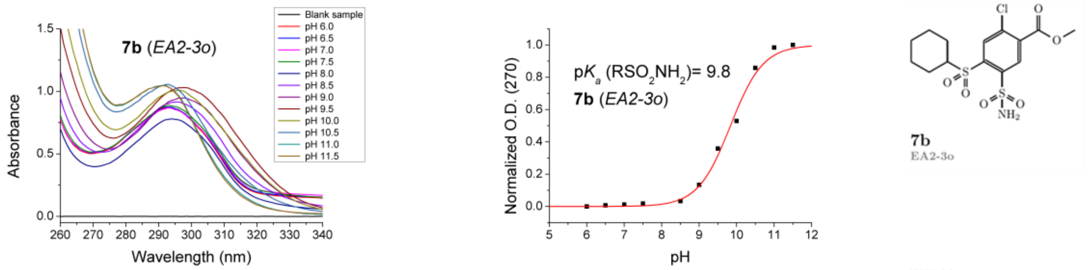

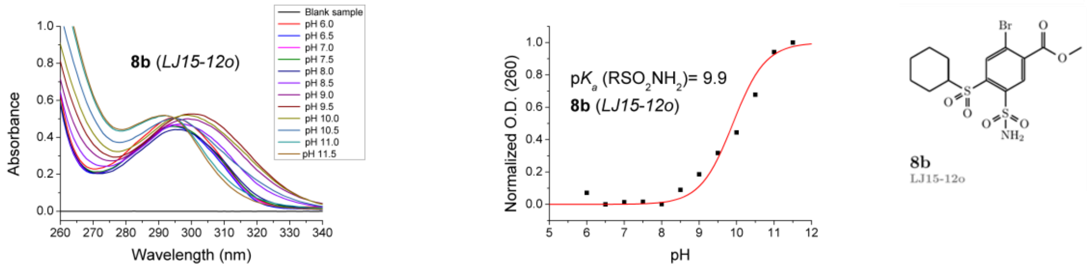

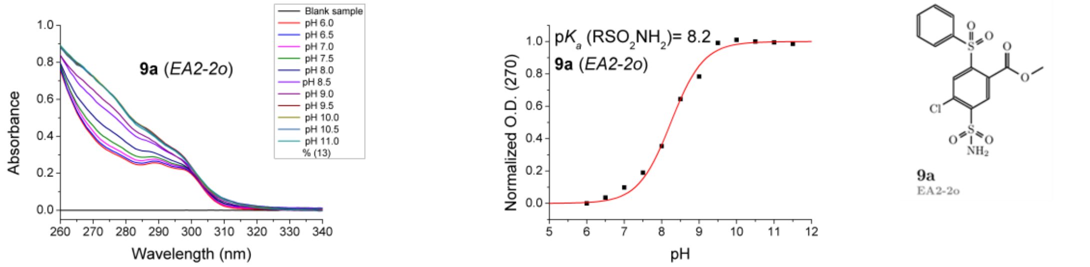

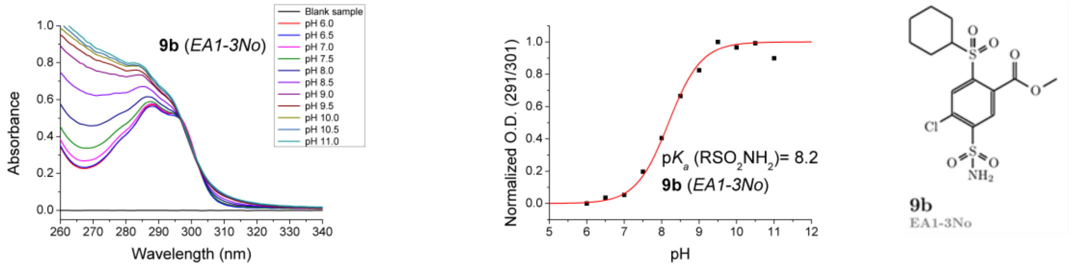

Continued on next page...

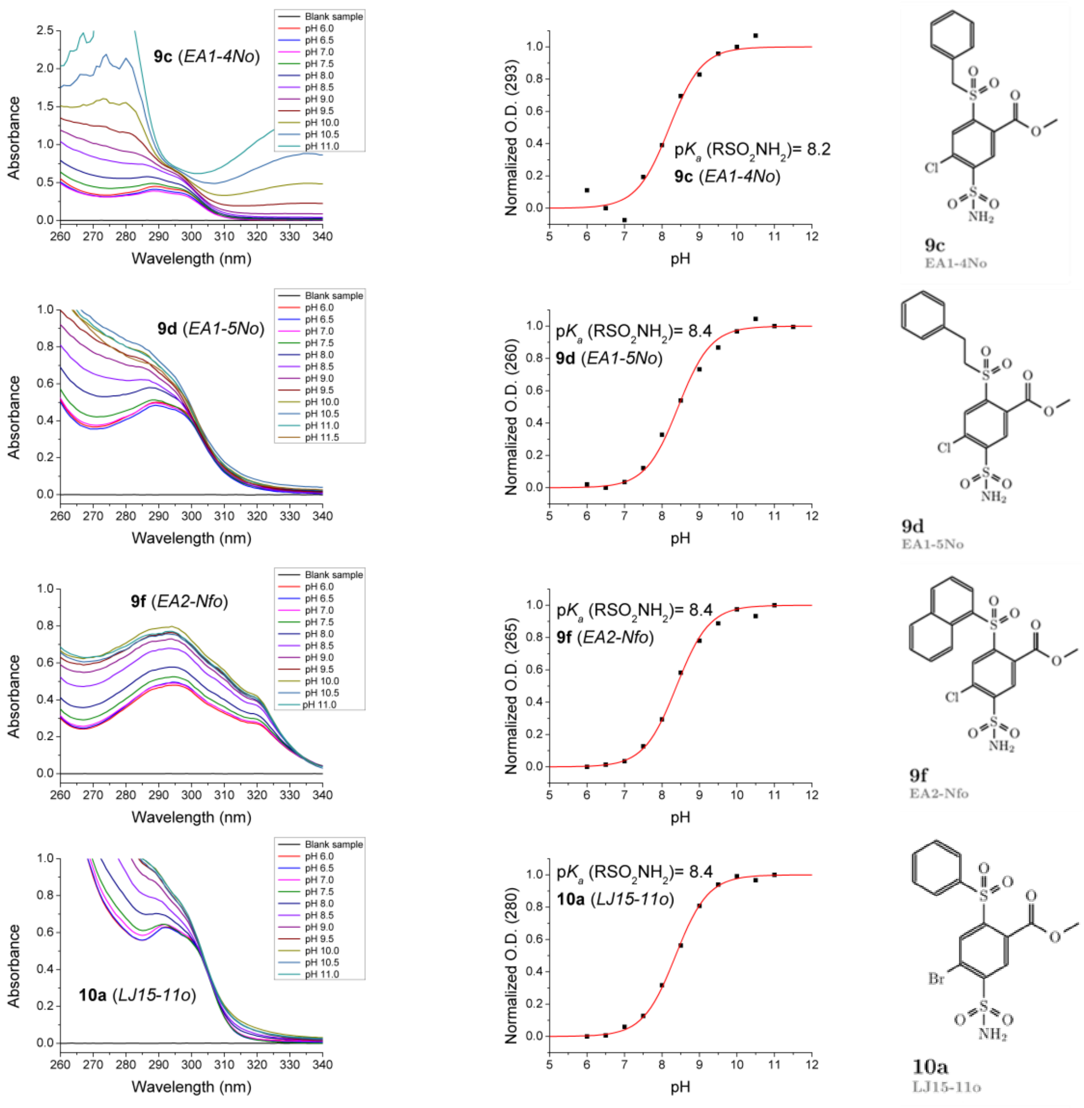

Figure S5. Determination of p$K_{a_RSO_2NH_2}$ values of sulfonamide group by UV-VIS spectrophotometry. (Left panels) UV absorbance spectra of the compound solution in buffers of various pH values. (Middle panels) Dependence of normalized absorbance or ratio of absorbances (approximately 10 nm above and 10 nm below the isosbestic point, if it is identifiable in the shown spectra) on pH, which is used to calculate the pK_a. Chemical structures of the investigated compounds are shown on the right. The pK_a values are also listed in Table 1.

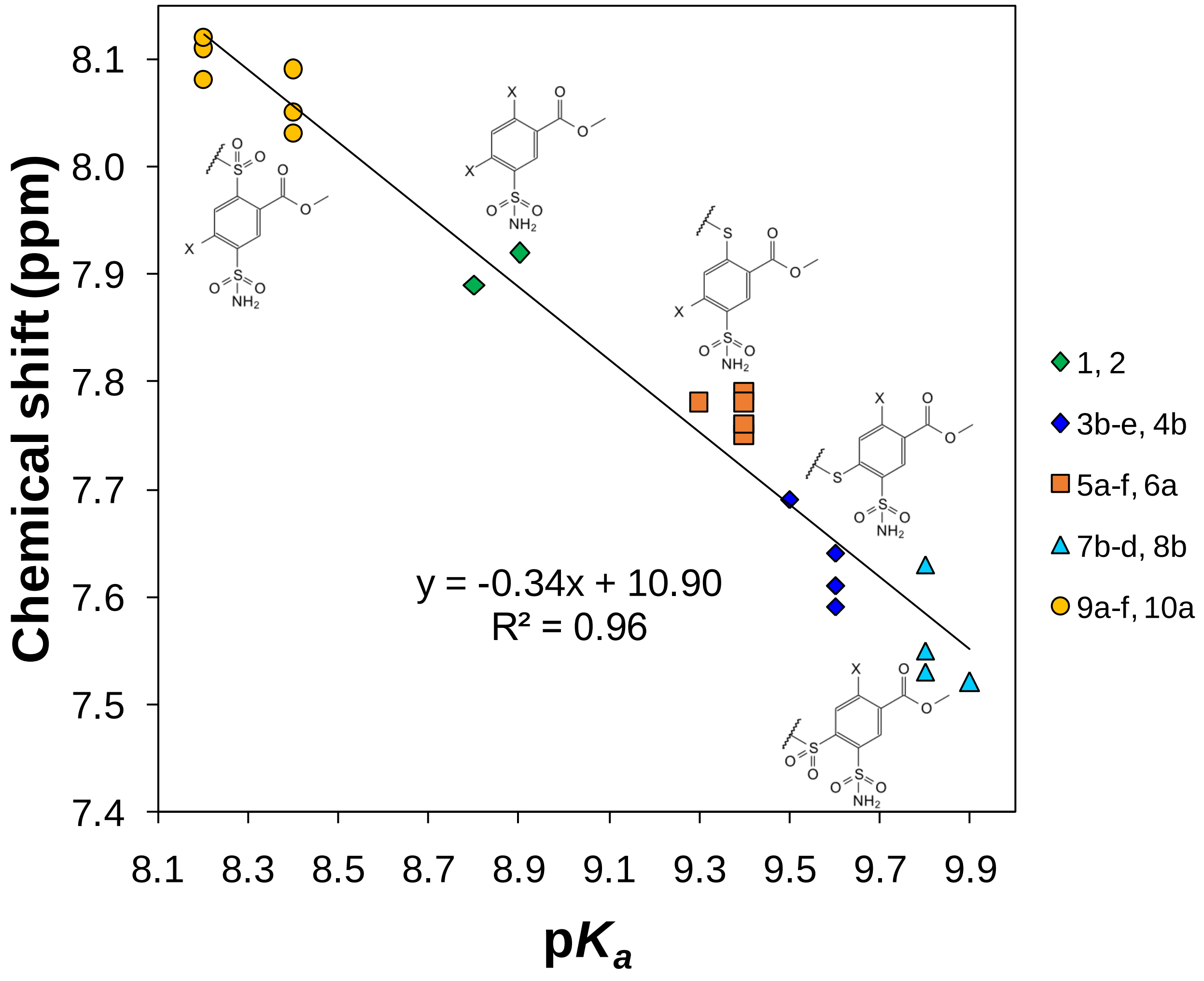

Figure S6. Correlation between the p$K_{a_RSO2NH2}$ of sulfonamide group and the chemical shift of sulfonamide amino group protons determined by NMR. Colors and symbols represent groups of compounds with similar chemical structures.

Standard enthalpy change upon protonation of inhibitor –RSO_2NH_2

<table>
<tr>
<td>

9a (*EA2-2o*)

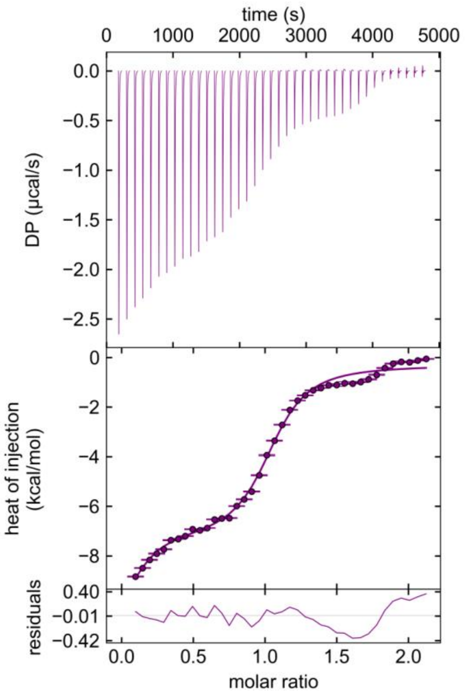

$\Delta_{p\_RSO_2NH_2}H$ = -6.91 kcal/mol, $\Delta H'$ = -11.108 kcal/mol

</td>
<td>

5b (*EA1-3N*)

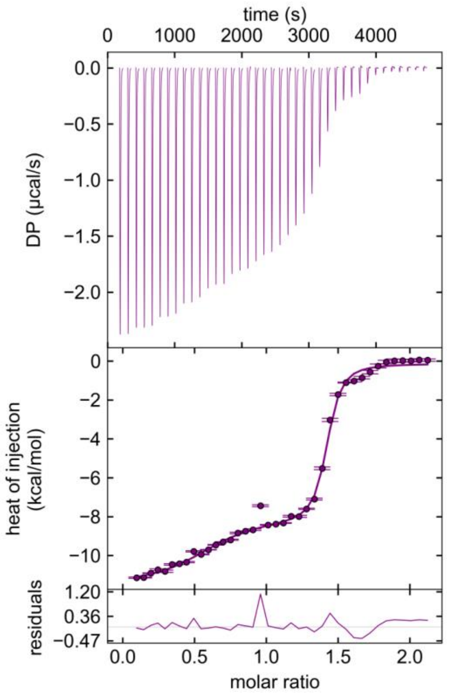

$\Delta_{p\_RSO_2NH_2}H$ = -7.96 kcal/mol, $\Delta H'$ = -11.413 kcal/mol

</td>
</tr>
<tr>
<td>

3b (*EA2-3*)

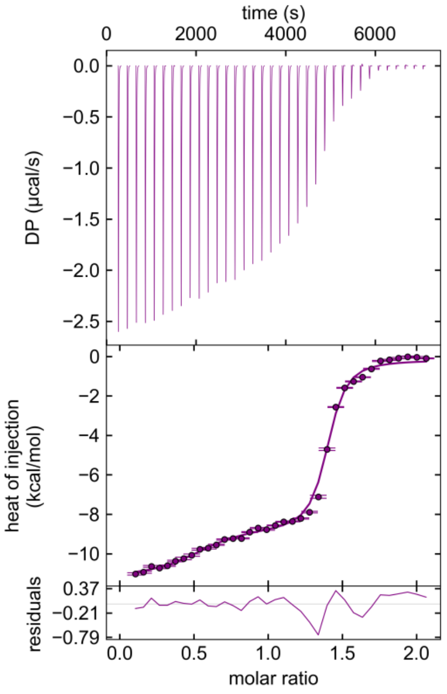

$\Delta_{p\_RSO_2NH_2}H$ = -8.53 kcal/mol, $\Delta H'$ = -11.05 kcal/mol

</td>
<td>

3d (*EA2-5*)

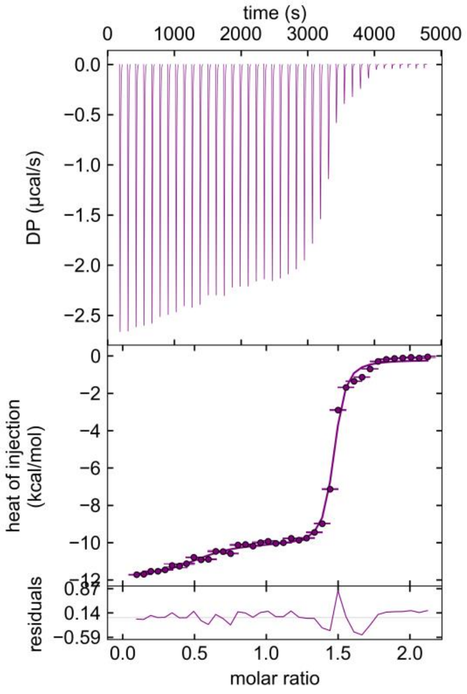

$\Delta_{p\_RSO_2NH_2}H$ = -9.81 kcal/mol, $\Delta H'$ = -11.63 kcal/mol

</td>
</tr>
</table>

...continued from previous page...

Figure S7. The raw and fitted ITC curves for the determination of the enthalpy change (Table S5) of sulfonamide group protonation, $\Delta_{p\_RSO2NH2}H$, at 37 °C. (Upper plots in each panel) ITC raw titration curves of 0.25 mM compound **3b**, **3d**, **5b** or **9a** in water with added 1.5 equivalent of NaOH titration with 2.75 mM HNO_3 at 37°C. (Lower plot in each panel) The integrated ITC curves: the first transition represents the neutralization of NaOH (ΔH') while the second transition represents the titration of the compound ($\Delta_{p\_RSO2NH2}H$). See the methods section for a more detailed description.

Table S5. The changes in protonation enthalpy of compound sulfonamide group at 37 °C.

Compound	$\Delta_{p\_RSO2NH2}H$ (kJ/mol)
**9a** (*EA2-2o*)	-28.9
**5b** (*EA1-3N*)	-33.3
**3b** (*EA2-3*)	-35.7
**3d** (*EA2-5*)	-41.0
**EA3-3**	-32.6
**EA3-2o**	-26.8

ITC data of selected compound binding to CA isoenzymes

CAXII – **5b** (***EA1-3N***)
(2 repeats)

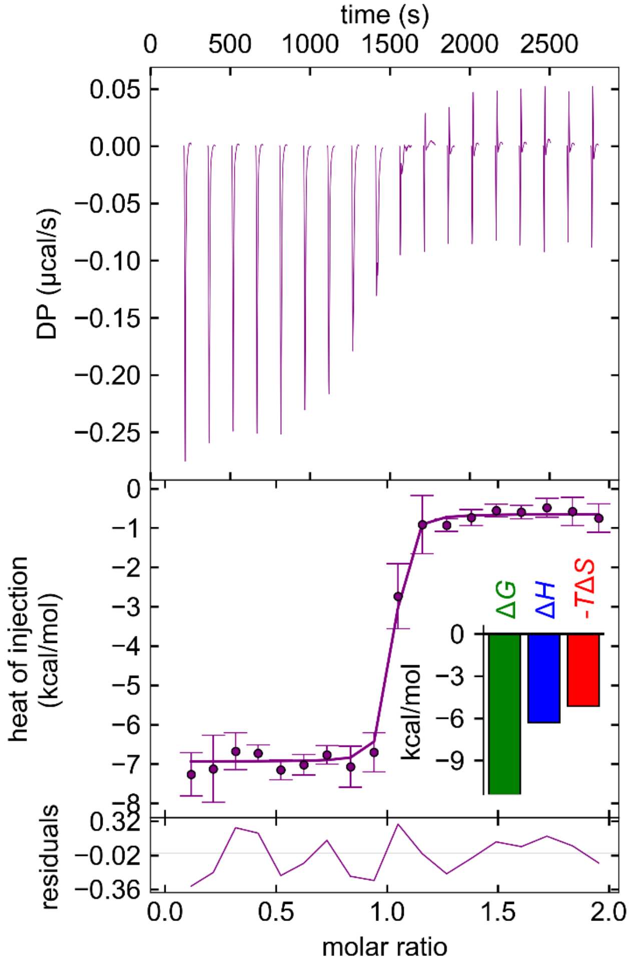

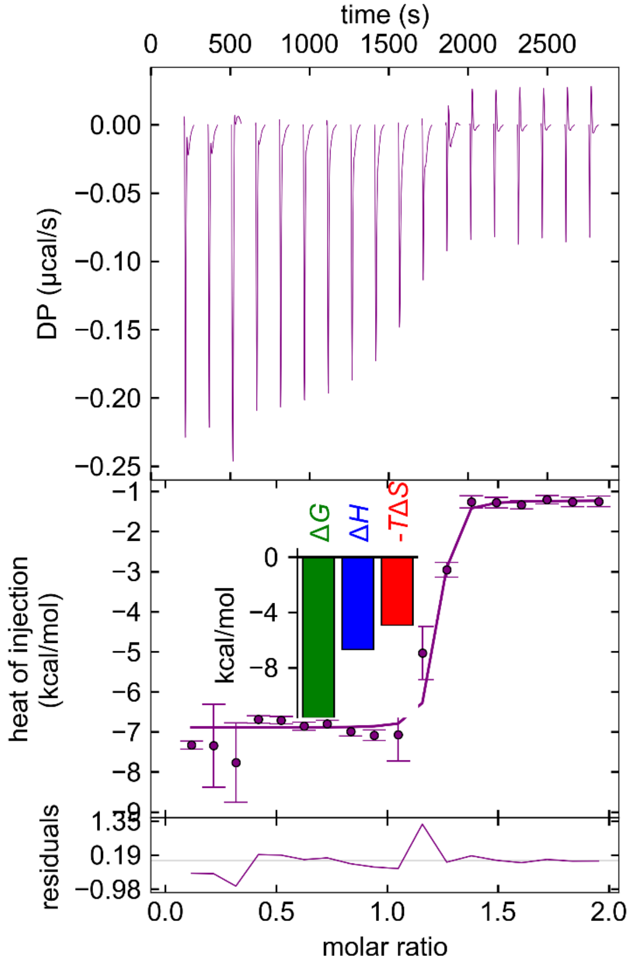

Δ*G*	-11.4 kcal/mol	-11.6 kcal/mol
Δ*H*	-6.3 kcal/mol	-6.7 kcal/mol
-*T*Δ*S*	-5.1 kcal/mol	-4.9 kcal/mol
N	1.0	1.1

Continued on next page...

CAXII – **9a** (***EA2-2o***)
(2 repeats)

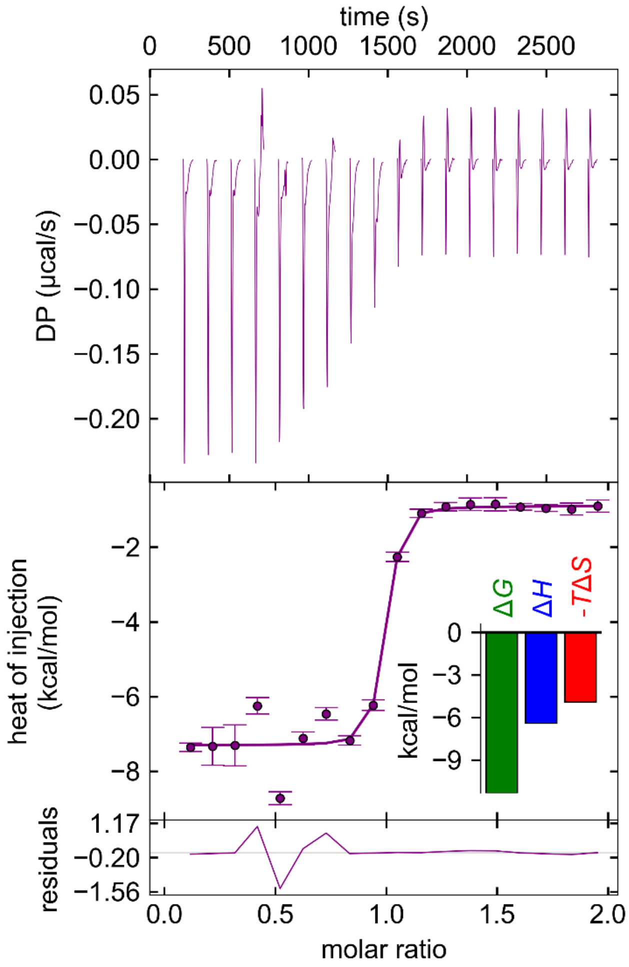

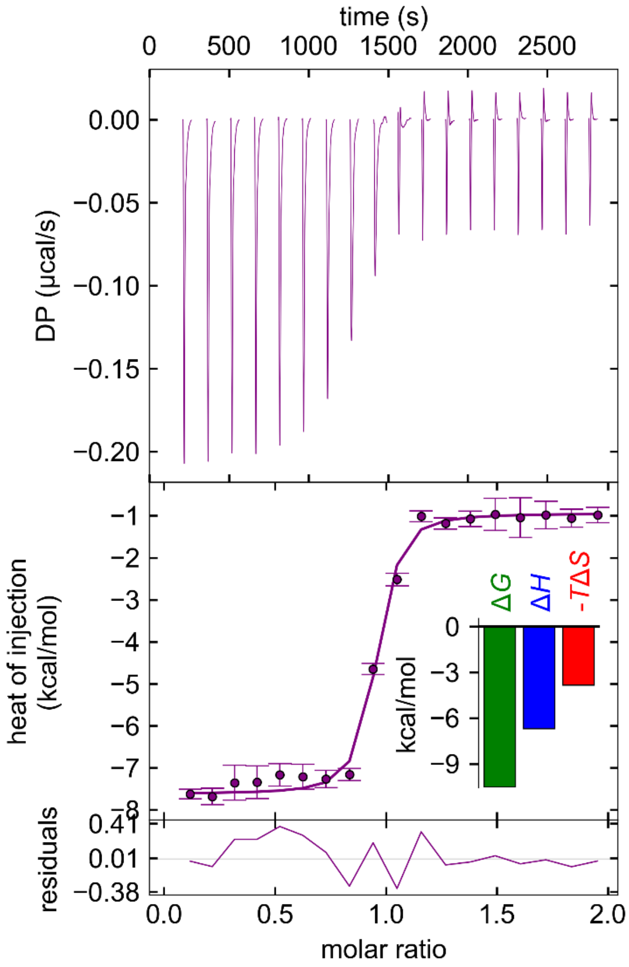

Δ*G*	-11.3 kcal/mol	-10.5 kcal/mol
Δ*H*	-6.4 kcal/mol	-6.7 kcal/mol
-*T*Δ*S*	-4.9 kcal/mol	-3.6 kcal/mol
N	1.0	0.9

Continued on next page...

CAXII – **3b** (***EA2-3***)
(2 repeats)

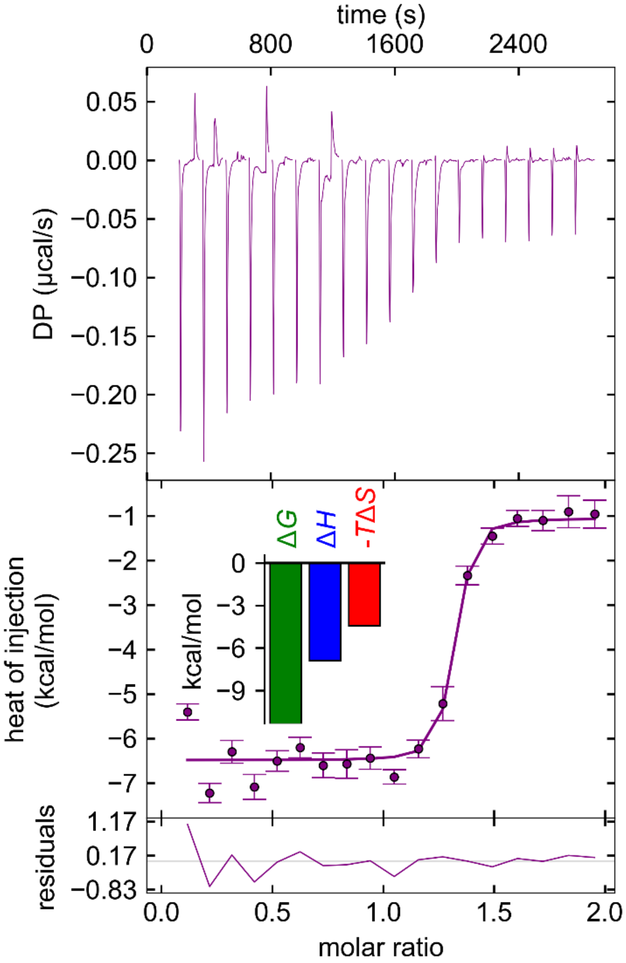

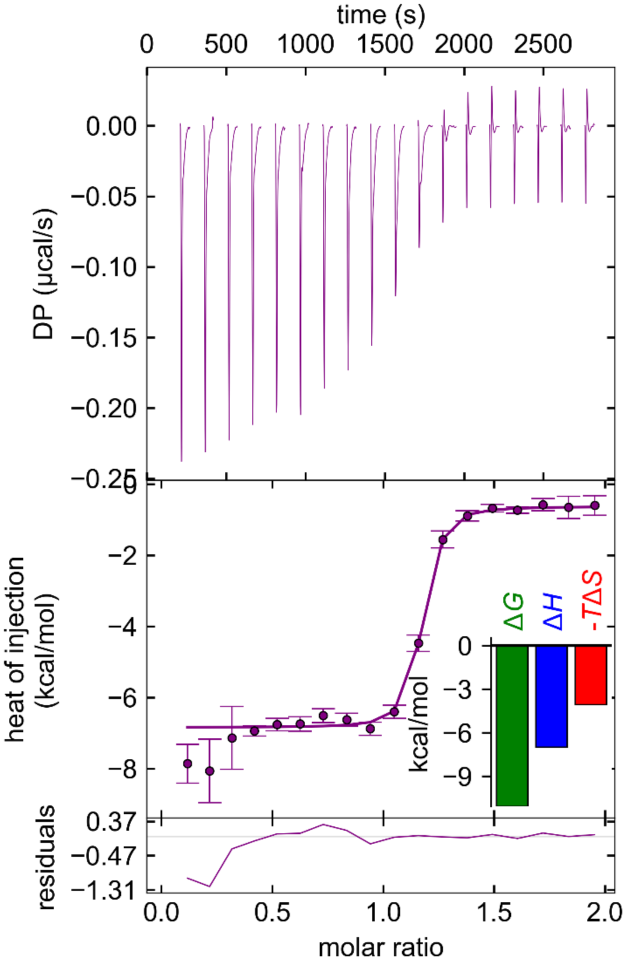

Δ*G*	-11.4 kcal/mol	-11.1 kcal/mol
Δ*H*	-6.9 kcal/mol	-7.0 kcal/mol
-*T*Δ*S*	-4.4 kcal/mol	-4.1 kcal/mol
N	1.2	1.1

Continued on next page...

CAXII – **3d (*EA2-5*)**
(2 repeats)

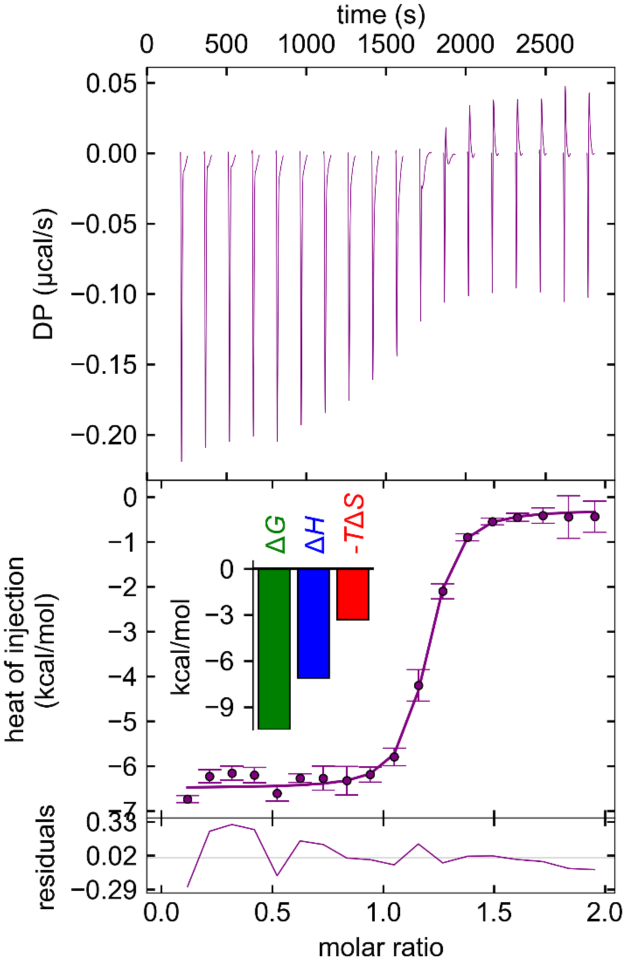

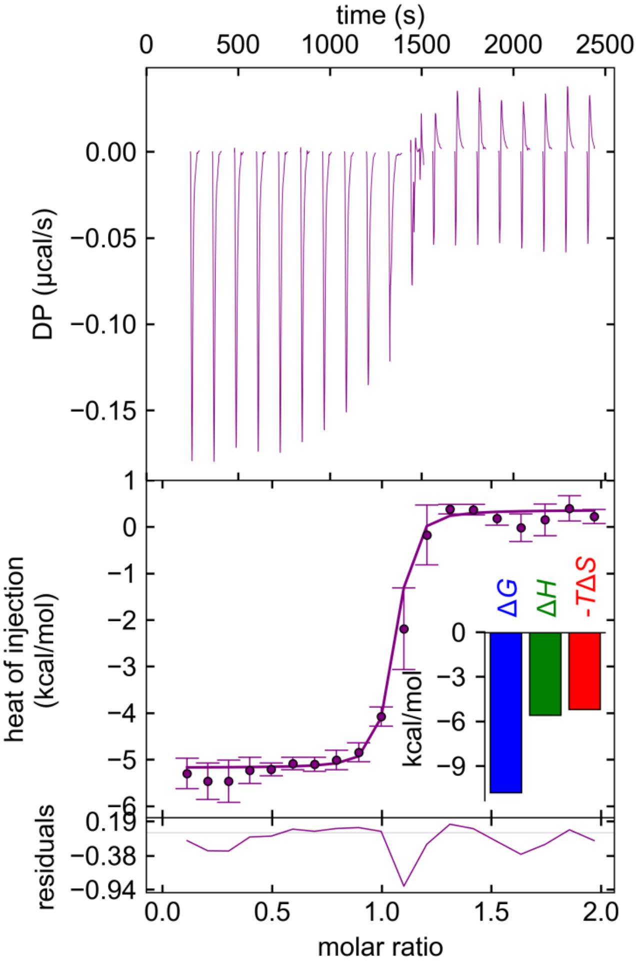

Δ*G*	-10.6 kcal/mol	-10.8 kcal/mol
Δ*H*	-6.8 kcal/mol	-5.6 kcal/mol
-*T*Δ*S*	-3.8 kcal/mol	-5.2 kcal/mol
N	1.1	1.0

Continued on next page...

CAI – **9a (*EA2-2o*)**
(2 repeats)

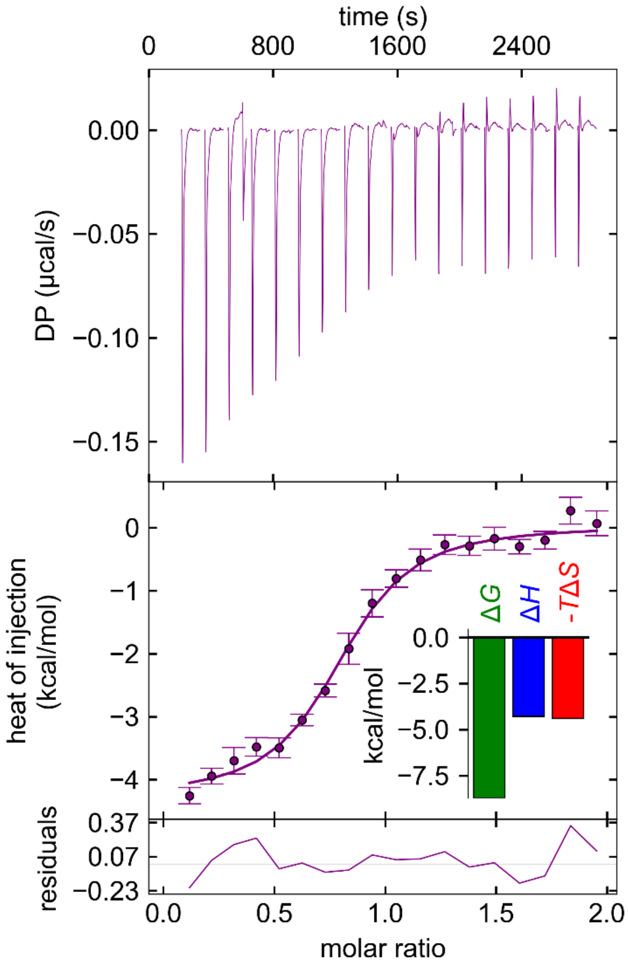

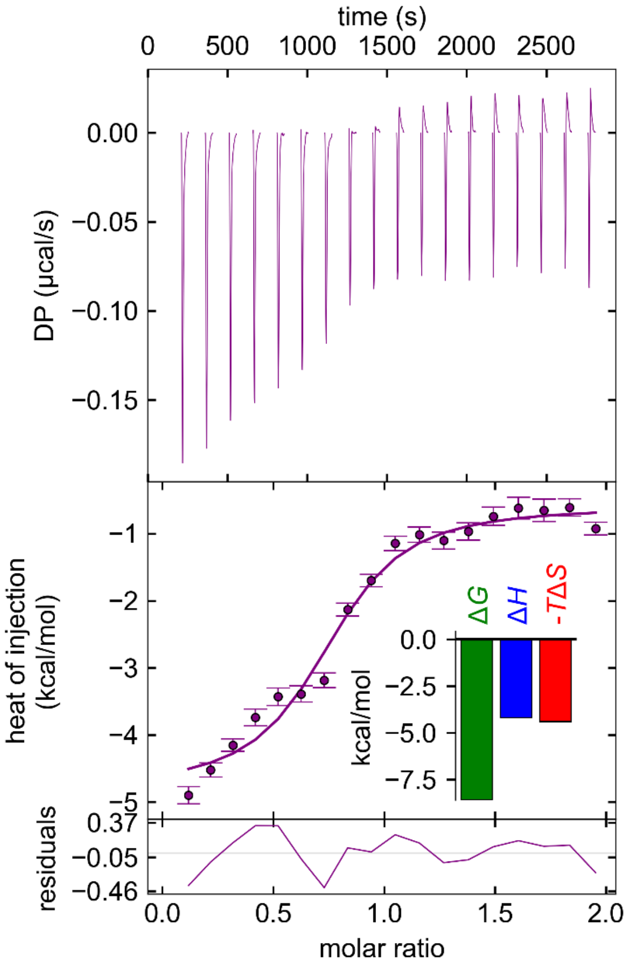

Δ*G*	-8.7 kcal/mol	-8.6 kcal/mol
Δ*H*	-4.3 kcal/mol	-4.2 kcal/mol
-*T*Δ*S*	-4.4 kcal/mol	-4.4 kcal/mol
N	0.8	0.7

Continued on next page...

CAII – **9a (*EA2-2o*)**
(2 repeats)

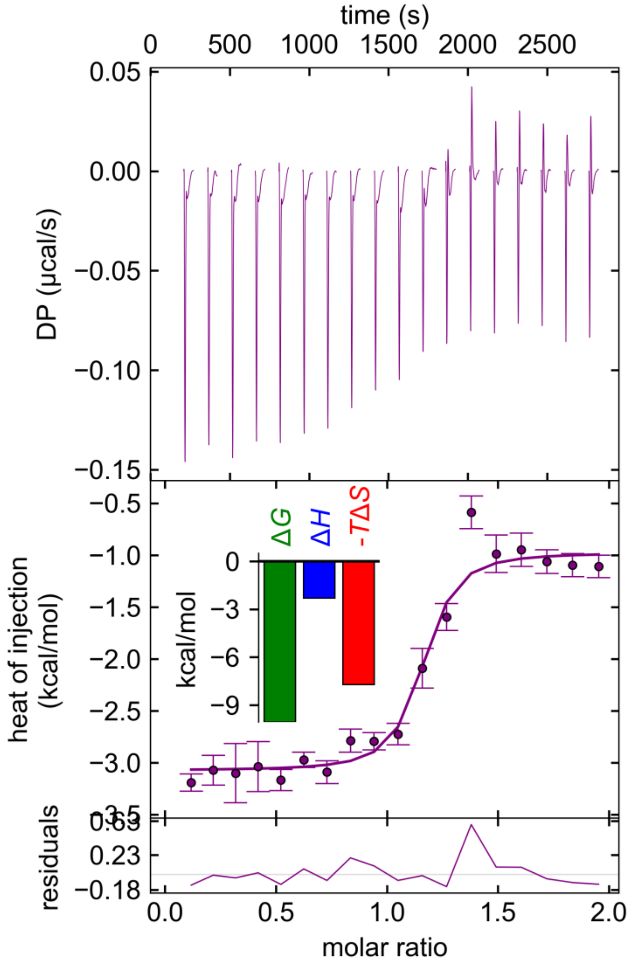

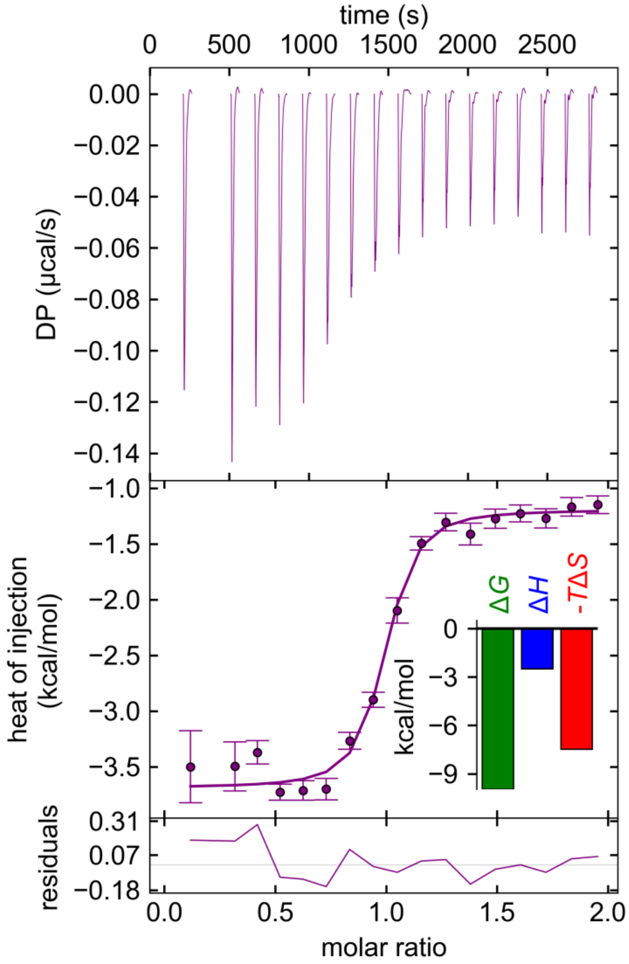

Δ*G*	-10.1 kcal/mol	-10.0 kcal/mol
Δ*H*	-2.4 kcal/mol	-2.5 kcal/mol
-*T*Δ*S*	-7.7 kcal/mol	-7.5 kcal/mol
N	1.1	1.0

Continued on next page...

CAIX – **3b (*EA2-3*)**
(2 repeats)

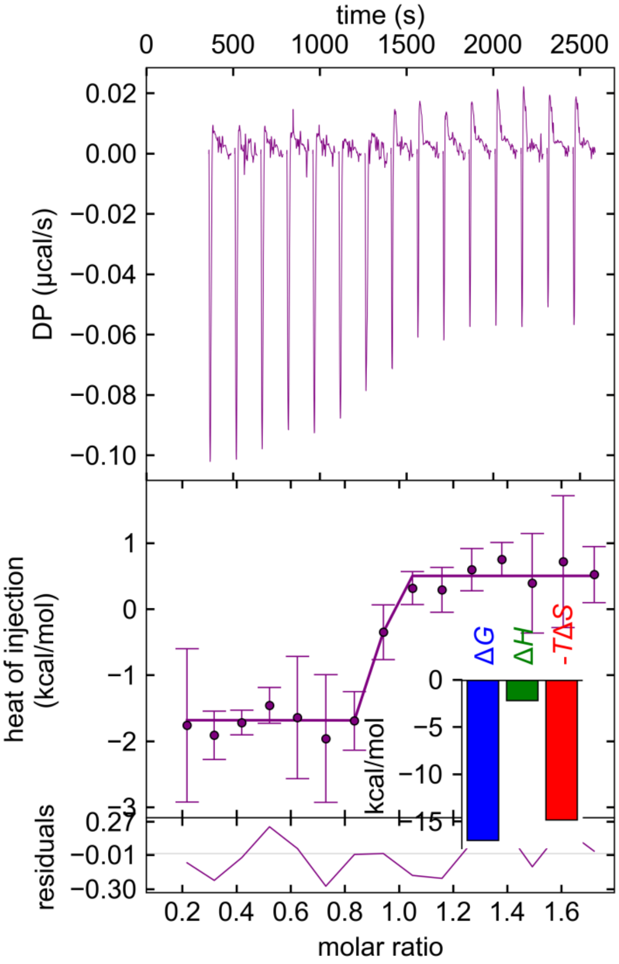

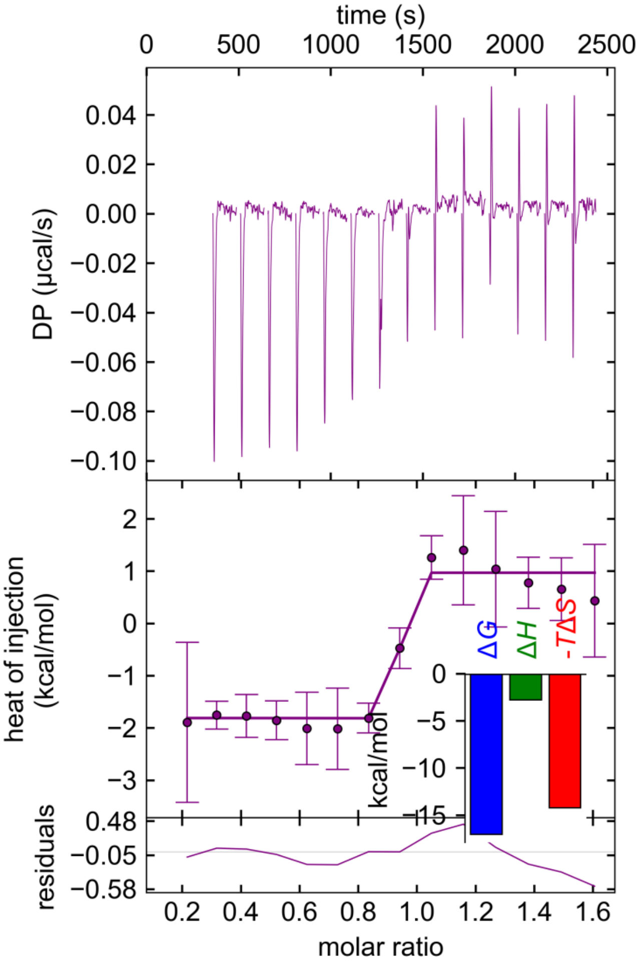

Δ*G*	-17.0 kcal/mol – too steep	-17.0 kcal/mol – too steep
Δ*H*	-2.2 kcal/mol	-2.8 kcal/mol
-*T*Δ*S*	-14.8 kcal/mol	-14.2 kcal/mol
N	0.9	0.9

Continued on next page...

Mimic CAIX (CAII mutant) – **3b (*EA2-3*)**
(2 repeats)

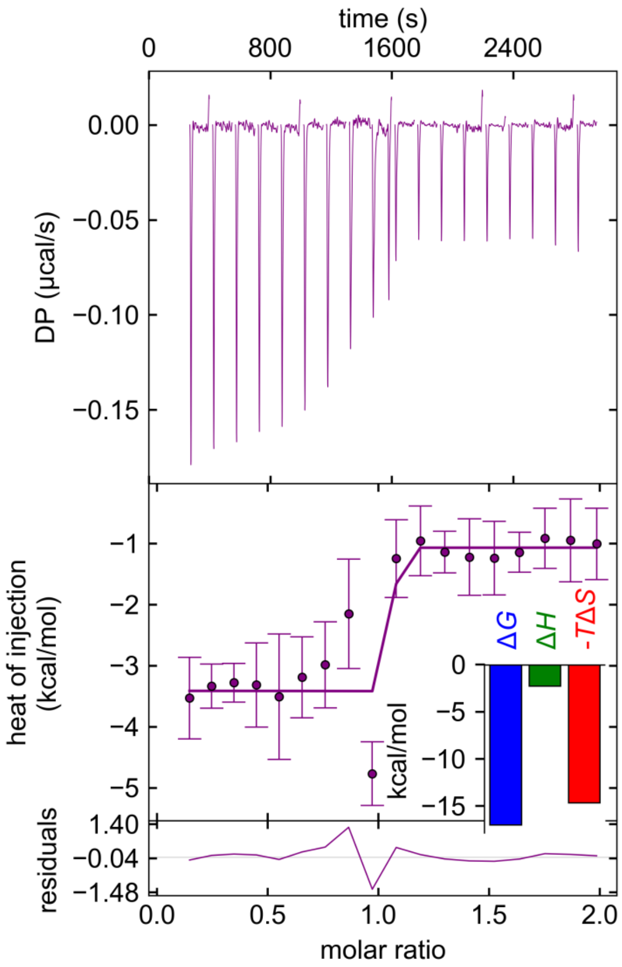

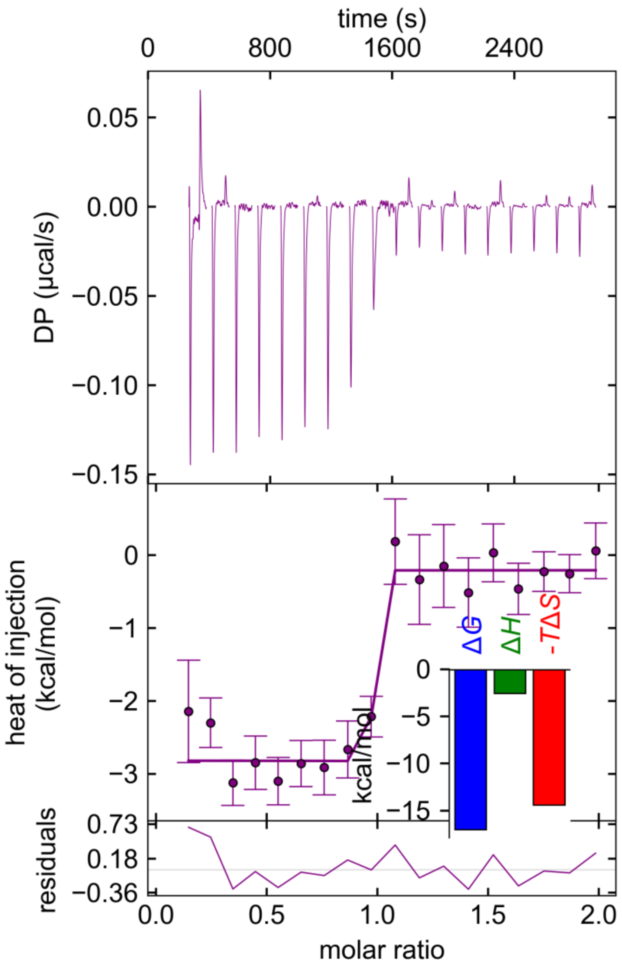

Δ*G*	-17.0 kcal/mol – too steep	-17.0 kcal/mol – too steep
Δ*H*	-2.2 kcal/mol	-2.6 kcal/mol
-*T*Δ*S*	-14.8 kcal/mol	- 14.4 kcal/mol
N	1.0	1.0

Continued on next page...

CAXII – **EA3-2o**
(2 repeats)

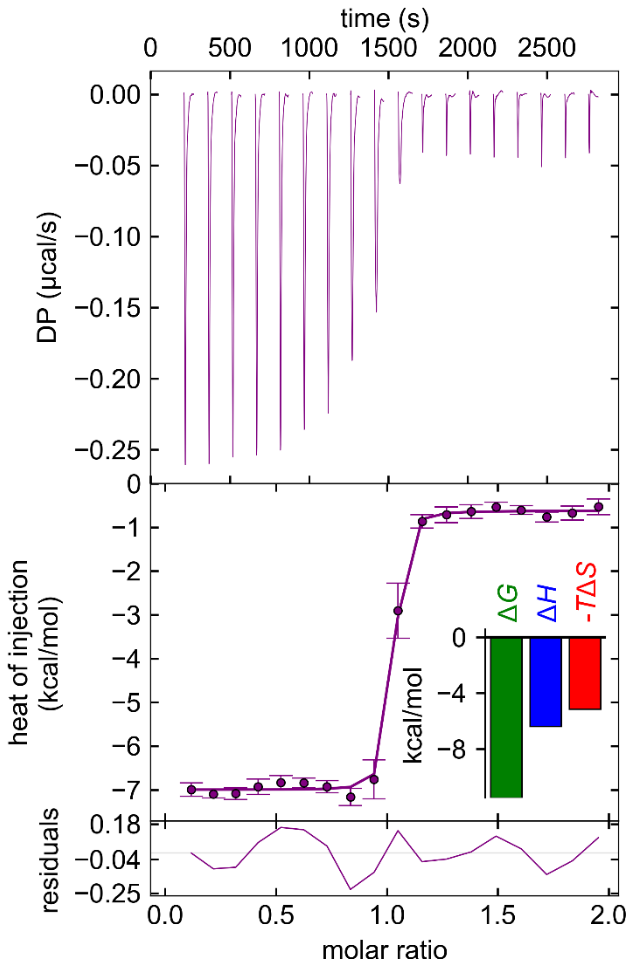

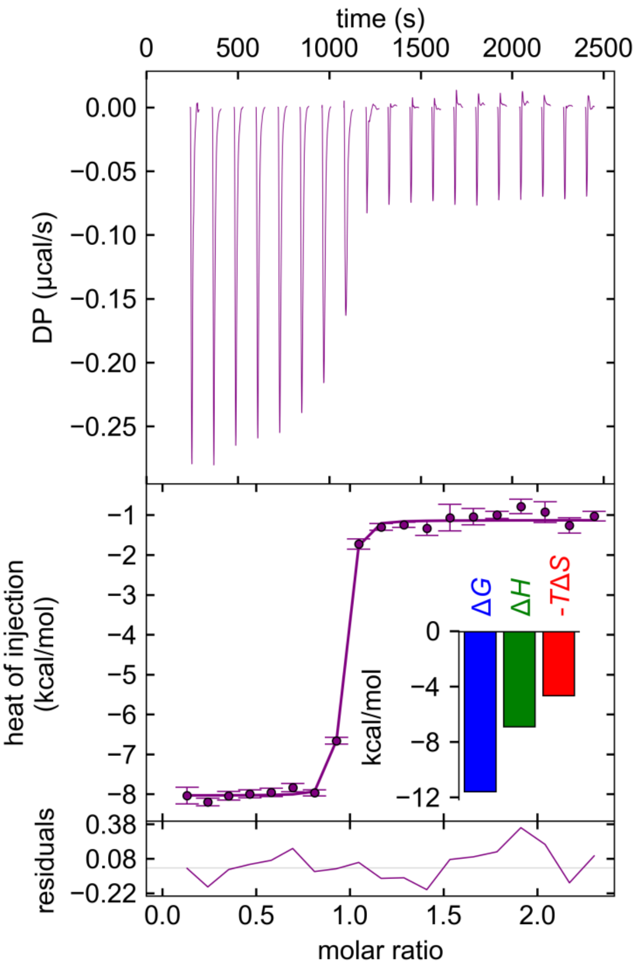

ΔG	-11.5 kcal/mol	-11.6 kcal/mol
ΔH	-6.4 kcal/mol	-6.9 kcal/mol
$-T\Delta S$	-5.1 kcal/mol	-4.7 kcal/mol
N	1.0	0.9 kcal/mol

Continued on next page...

CAXII – **EA3-3**
(2 repeats)

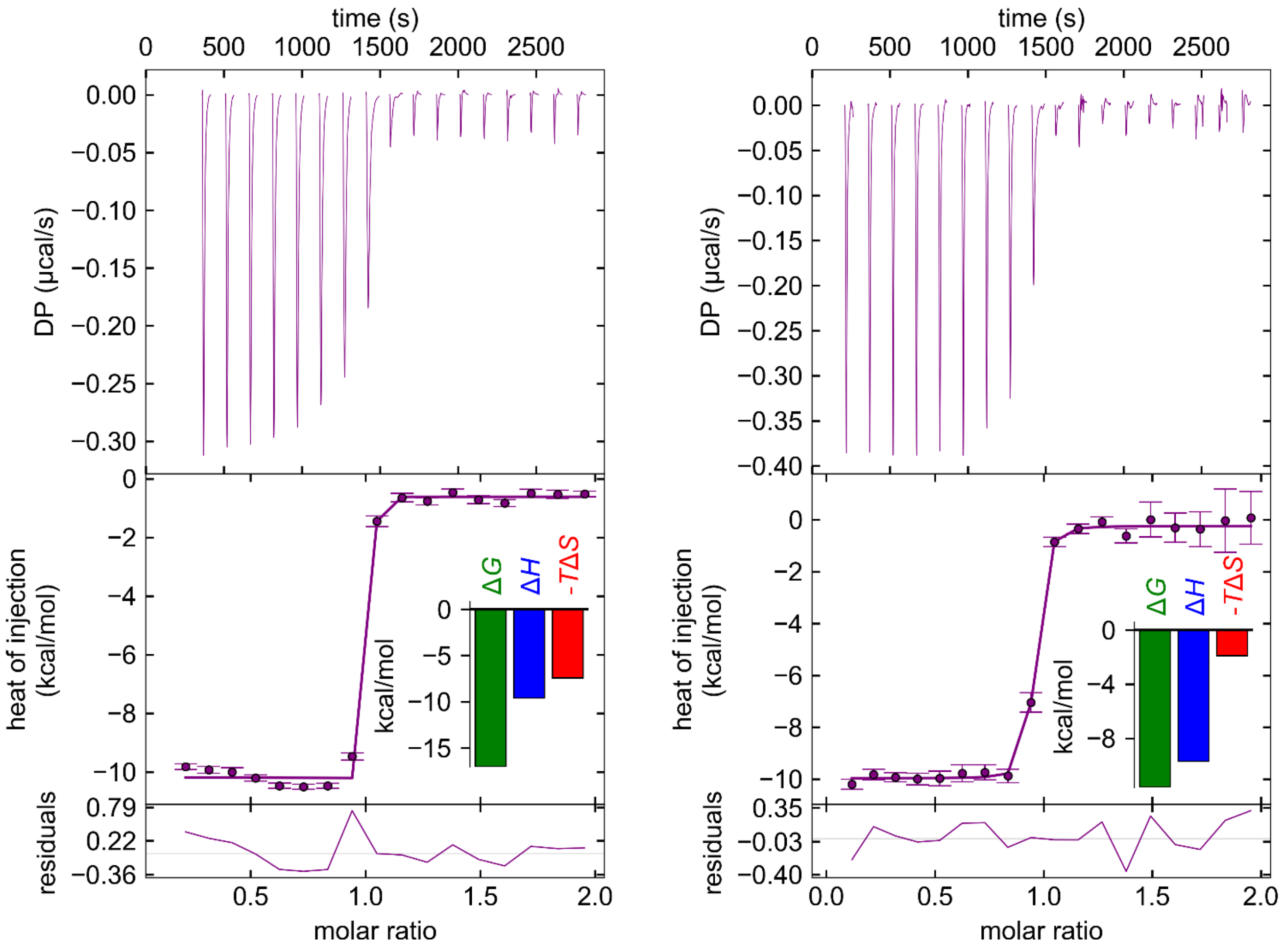

Δ*G*	~~-17.0~~ kcal/mol – too steep	-11.6 kcal/mol
Δ*H*	-9.6 kcal/mol	-9.7 kcal/mol
-*T*Δ*S*	-7.5 kcal/mol	-1.9 kcal/mol
N	1.0	0.9

Figure S8. Isothermal titration calorimetry data obtained by iTC200 microcalorimeter. (Top plots in each panel) ITC data obtained by injecting syringe solution containing 200 µM compound into the cell containing 20 µM CA, both solutions contained the same sodium phosphate buffer at pH 7.0 and the experiment was performed isothermally at 37 °C. (Bottom plots in each panel) Integrated data plotted as a function of the molar ratio of compound and CA. Lines show the fits to a single-site model. The *observed* fitted parameters are listed below each graph.

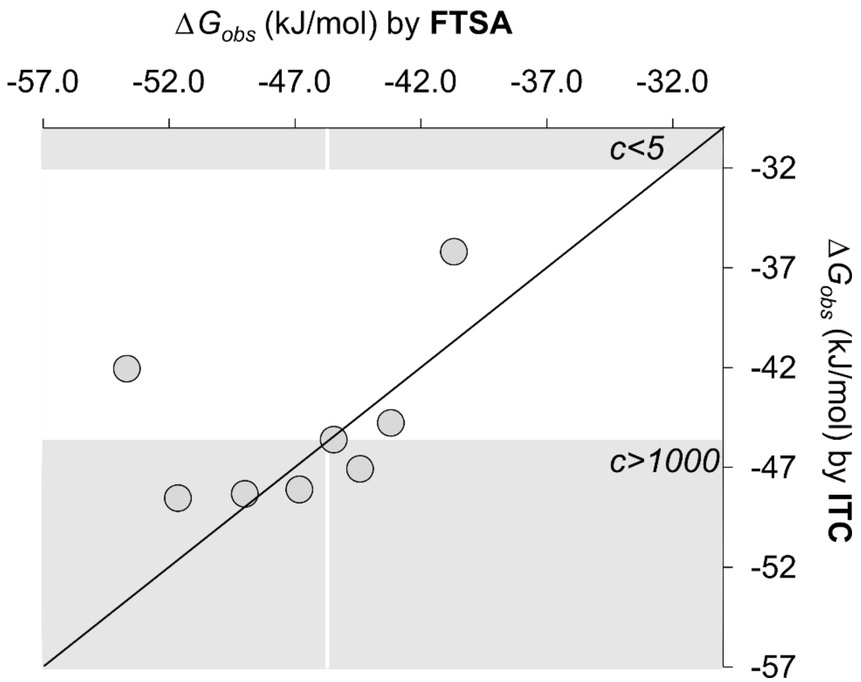

Figure S9. Correlation of the *observed* affinities determined by FTSA and ITC techniques. The corresponding values of FTSA and ITC data were taken from Table S1 and Table S6, respectively. Affinity values are more accurately measured by FTSA because the dynamic range of FTSA is much greater of ITC (Linkuvienė et al. 2018) and were therefore used for further analysis. The gray area shows the situations where the affinities cannot be determined by direct ITC measurements.

Table S6. The *observed* standard enthalpy changes upon compound binding to CA. Values are in kJ/mol listed for several selected compound binding to several CA isoenzymes in sodium phosphate buffer at pH 7.0 and 37 °C determined by ITC. The experimental data are shown in Figure S8.

Compound	CAI	CAII	CAIX	mimic CAIX	CAXII
**3d** *(EA2-5)*	ND	ND	ND	ND	-25.9
**3b** *(EA2-3)*	ND	ND	-10.5	-10.0	-29.1
**5b** *(EA1-3N)*	ND	ND	ND	ND	-27.2
**9a** *(EA2-2o)*	-17.8	-10.3	ND	ND	-27.4
**EA3-3**	ND	ND	ND	ND	-40.4
**EA3-2o**	ND	ND	ND	ND	-27.8

Table S7. *Intrinsic* standard enthalpy changes (kJ/mol) of compound binding to CA isoenzymes at 37 °C. Values are independent of pH and buffer and were calculated according to equations S5-S8 in the next section. The table also contains the data used for the calculations: protonation enthalpies $\Delta_{p\_RSO2NH2}H$ – this study (Figure S7 and Table S5), $\Delta_{p\_\ CAZn(II)H2O}H$ – (Linkuvienė et al. 2018); $pK_{a\_RSO2NH2}$ – this study (Figure S5 and Table 1), $pK_{a\_CAZn(II)H2O}$ – [1]; fractions at pH7.0 of deprotonated sulfonamide, f_{RSO2NH^-}, and CA-Zn(II)-bound water molecule, $f_{CA\text{-}Zn(II)H2O}$ – calculated according to equations S2-S3; n_{RSONH2} and $n_{CAZn(II)H2O}$ – calculated according to equations S6-S7.

					CAI	CAII	CAIX	mimic CAIX	CAXII
				$\Delta_{p\_\ CAZn(II)H2O}H$	-38.5	-23.5	-21.5	-23.5	-25.5
				$pK_{a\_CAZn(II)H2O}$	8.1	6.9	6.6	6.9	6.8
				$f_{CAZn(II)H2O}$	92.64%	44.27%	28.47%	44.27%	38.69%
Compound	$\Delta_{p\_RSO2NH2}H$	$pK_{a\_RSO2NH2}$	f_{RSONH^-}	$n_{CAZn(II)H2O}$ n_{RSONH2}	0.07	0.56	0.72	0.56	0.61
**3d** *(EA2-5)*	-41.0	9.6	0.25%	-1.00	ND	ND	ND	ND	-50.1
**3b** *(EA2-3)*	-35.7	9.6	0.25%	-1.00	ND	ND	-29.9	-31.2	-48.0
**5b** *(EA1-3N)*	-33.3	9.4	0.40%	-1.00	ND	ND	ND	ND	-43.6
**9a** *(EA2-2o)*	-28.9	8.2	5.94%	-0.94	-39.7	-23.3	ND	ND	-38.0
**EA3-3**	-32.6	9.0	0.99%	-0.99	ND	ND	ND	ND	-39.5
**EA3-2o**	-26.8	8.3	4.77%	-0.95	ND	ND	ND	ND	-21.9

Equations for calculation of K_{d_intr}, ΔG_{intr} and ΔH_{intr}

The intrinsic dissociation constant is equal to:

$$K_{d\_intr} = K_{d\_obs} \times f_{\mathrm{RSO_2NH^-}} \times f_{\mathrm{CAZn(II)H_2O}} \quad \text{(S1)}$$

$$f_{\mathrm{RSO_2NH^-}} = \frac{10^{\mathrm{pH}-pK_{a\_\mathrm{RSO2NH2}}}}{1+10^{\mathrm{pH}-pK_{a\_\mathrm{RSO2NH2}}}} \quad \text{(S2)}$$

$$f_{\mathrm{CAZn(II)H_2O}} = 1 - \frac{10^{\mathrm{pH}-pK_{a\_\mathrm{CAZn(II)H_2O}}}}{1+10^{\mathrm{pH}-pK_{a\_\mathrm{CAZn(II)H_2O}}}} \quad \text{(S3)}$$

- K_{d_obs} - observed dissociation constant;
- $f_{\mathrm{RSO_2NH^-}}$ and$f_{\mathrm{CAZn(II)H_2O}}$ - fractions of deprotonated sulfonamide and Zn(II)-bound water molecule;
- $pK_{a\_\mathrm{RSO_2NH_2}}$- p$K_a$ of the sulfonamide group;
- $pK_{a\_\mathrm{CAZn(II)H_2O}}$ - pK_a value of water molecule bound to Zn(II) in the active site of CA;

In this study the pH value was always equal to 7.0.

The intrinsic standard Gibbs energy change can be calculated according to:

$$\Delta G_{intr} = \mathrm{RTln}K_{d\_intr} \quad \text{(S4)}$$

,

- where R is the universal ideal gas constant, T– temperature (here 310 K).

The intrinsic enthalpy change is equal to:

$$\Delta H_{intr} = \Delta H_{obs} - n_{\mathrm{RSO_2NH_2}}\Delta_{p\_\mathrm{RSO_2NH_2}}H - n_{\mathrm{CAZn(II)H_2O}}\Delta_{p\_\mathrm{CAZn(II)H_2O}}H + n_{\mathrm{buffer}}\Delta_{p\_\mathrm{buffer}}H \quad \text{(S5)}$$

$$n_{\mathrm{RSO_2NH_2}} = f_{\mathrm{RSO_2NH^-}} - 1 \quad \text{(S6)}$$

$$n_{\mathrm{CAZn(II)H_2}O} = 1 - f_{\mathrm{CAZn(II)H_2}O} \quad \text{(S7)}$$

$$n_{\mathrm{buffer}} = n_{\mathrm{RSO_2NH_2}} + n_{\mathrm{CAZn(II)H_2}O} \quad \text{(S8)}$$

- where ΔH_{obs} - the observed binding enthalpy;
- $n_{\mathrm{RSO_2NH_2}}$- the number of protons released from the sulfonamide group;
- $\Delta_{p\_\mathrm{RSO_2NH_2}}H$ - the enthalpy of sulfonamide group protonation;
- $n_{\mathrm{CAZn(II)H_2O}}$ - the number of protons bound to the CAZn(II)OH^-;
- $\Delta_{p\_\mathrm{CAZn(II)H_2O}}H$ - the enthalpy of OH^- protonation in the active site of CAZn(II);

- n_{buffer} - the net sum of up taken or released protons by buffer;
- $\Delta_{p\_\text{buffer}}H$- the enthalpy of buffer protonation: for Tris, $\Delta_{p\_\text{TRIS}}H$ is equal to -46.56 kJ/mol, and for phosphate buffer $\Delta_{p_\text{phosphate}}H$is equal to -2.88 kJ/mol at 37°C (Goldberg et al. 2002).

See more detailed description - (Zubrienė and Matulis 2019).

Determination of the regio selectivity of the nucleophilic aromatic substitution of a halogen in methyl 2,4-dihalo-5-sulfamoyl-benzoates 1, 2 with thiols

Synthesis

The mixture of methyl 2,4- dihalo-5-sulfamoylbenzoate **1** or **2** (0.07 mmol), DMSO (0.20 mL), appropriate aryl/alkylthiol (0.08 mmol) and TEA (0.02 mL, 0.14 mmol) was heated at 60 °C temperature for 3 days. 1 mL EtOAc was added to the reaction mixture and the product was purified by flash chromatography on a column of silica gel with EtOAc.

Determination of product ratio by HPLC

Chemicals and reagents

The HPLC-grade MeCN (Fisher Scientific) and ultrapure water (18.2 MΩ cm−1, Milli-Q Plus system, Millipore Bedford, MA, USA) were used for RP-HPLC separation.

Sample preparation

The reaction mixture containing primary compound and mono-substituted isomers was dissolved in the MeCN to a final 5 mg/mL concentration.

Instrumentation

To develop the analytical separation of sulfonamides, the Shimadzu UFLC system was employed, consisting of a CMB-20A communication module, two LC20AD quaternary and isocratic pumps, and a SIL-20AC autosampler, a CTO-20A column compartment and a SPD-M20A DAD detector (Shimazdu Corp., Japan). The diode-array detector wavelength range was set from 190 to 400 nm. The C18-PFP HPLC separation column (10 cm x 4.6 cm, 3 µm, ACE) was used.

Separation

The reverse-phase separation was developed in a C18 PFP column, and the trinary mobile phase consisted of water (eluent A), MeCN (eluent B), and water with 1% TFA (eluent C). The constant 10% C isocratic concentration was used to keep 0.1% TFA concentration in the column while using gradient conditions between eluents A and B. The initial conditions were established, equilibrating the column by 5 column

volumes (8.3 min). Then each chromatographic run was finalized with 8.3 minutes 90% B wash-out. The gradient elution when separating reaction mixtures of **1** and **2** with cyclohexanethiol, **1** and **2** with phenylthiol, **1** with ethylphenylthiol, **1** with cyclododecanethiol was 45% B (0-1 min) and 72% B (15 min), for **1** with benzylthiol was 40.5% B (0-1 min), 49.5% B (15 min) and 90% B (20 min) and for **1** with naphthylthiol was 40.5% B (0-1 min) and 63% B (15 min). The column thermostat was set to 40° C, and the flow rate was set to 1 mL/min. The detection was at 260 nm with a bandwidth of 4 nm, and the data rate was set to 6.25 Hz.

Quantification of the reaction products and conversion constant was carried out using calibration curves.

NMR spectra of synthesized compounds

Solvent signals: ^{13}C NMR chemical shift of DMSO-d_6 is 39.52 ppm (septet), ^{1}H NMR solvent residual peaks 2.50 ppm (DMSO) and 3.33 ppm (H_2O).

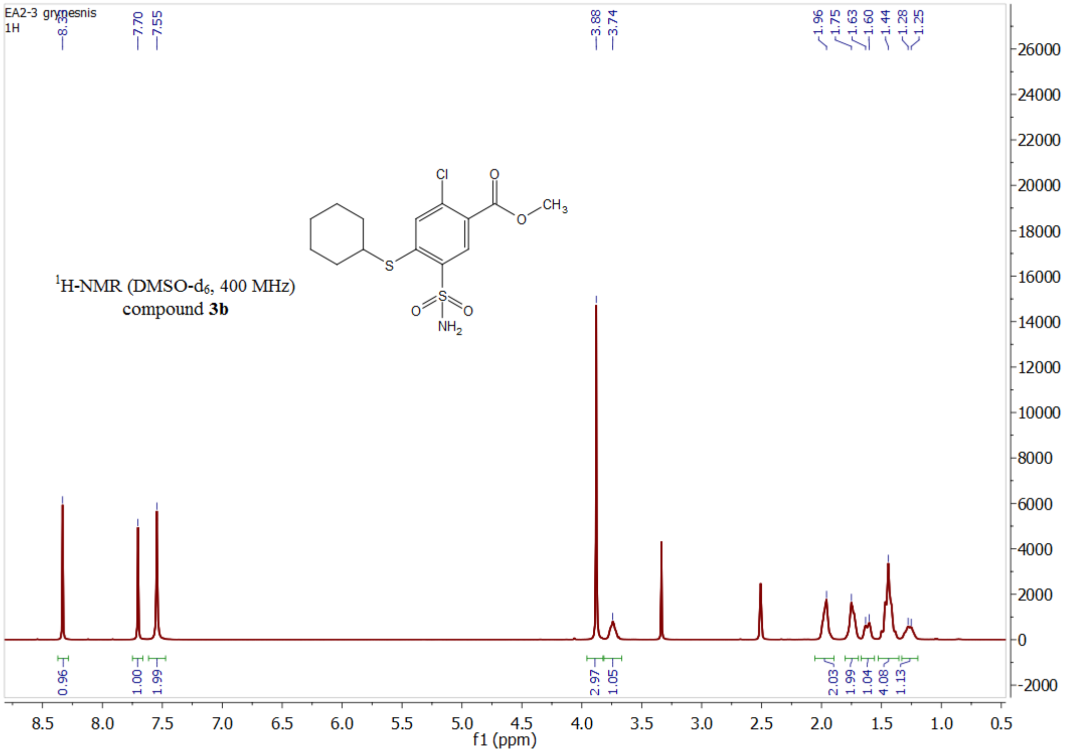

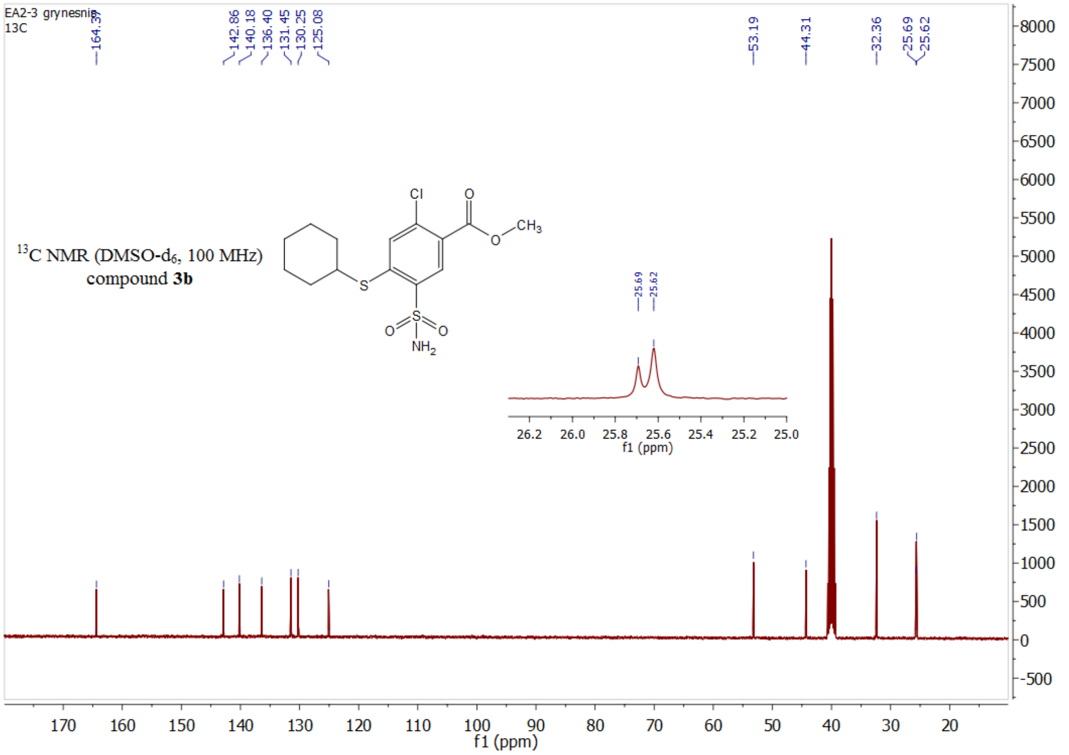

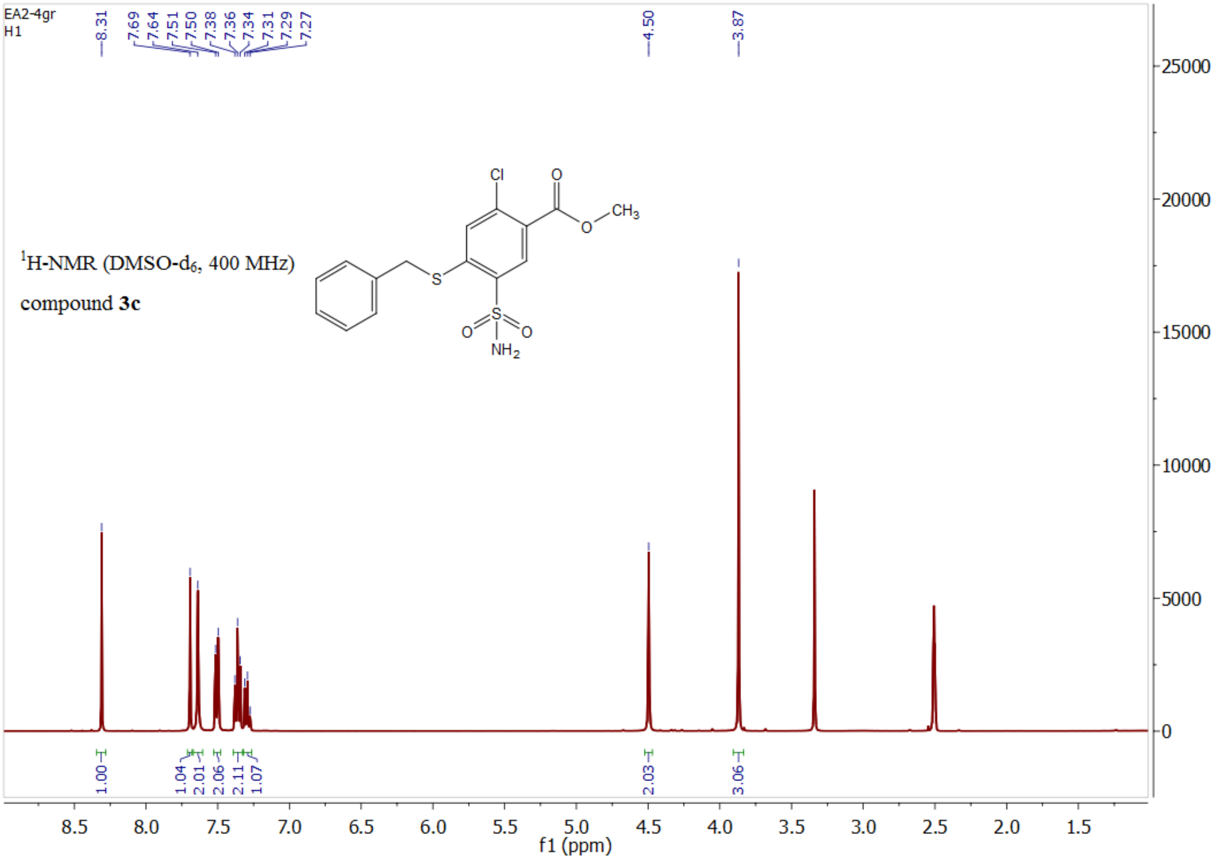
EA2-4gr
H1
8.31
7.69
7.64
7.51
7.50
7.38
7.36
7.34
7.31
7.29
7.27
4.50
3.87
Cl
O
O
CH3
S
O
S
O
NH2
1H-NMR (DMSO-d6, 400 MHz)
compound 3c
1.00
1.04
2.01
2.06
2.11
1.07
2.03
3.06
f1 (ppm)

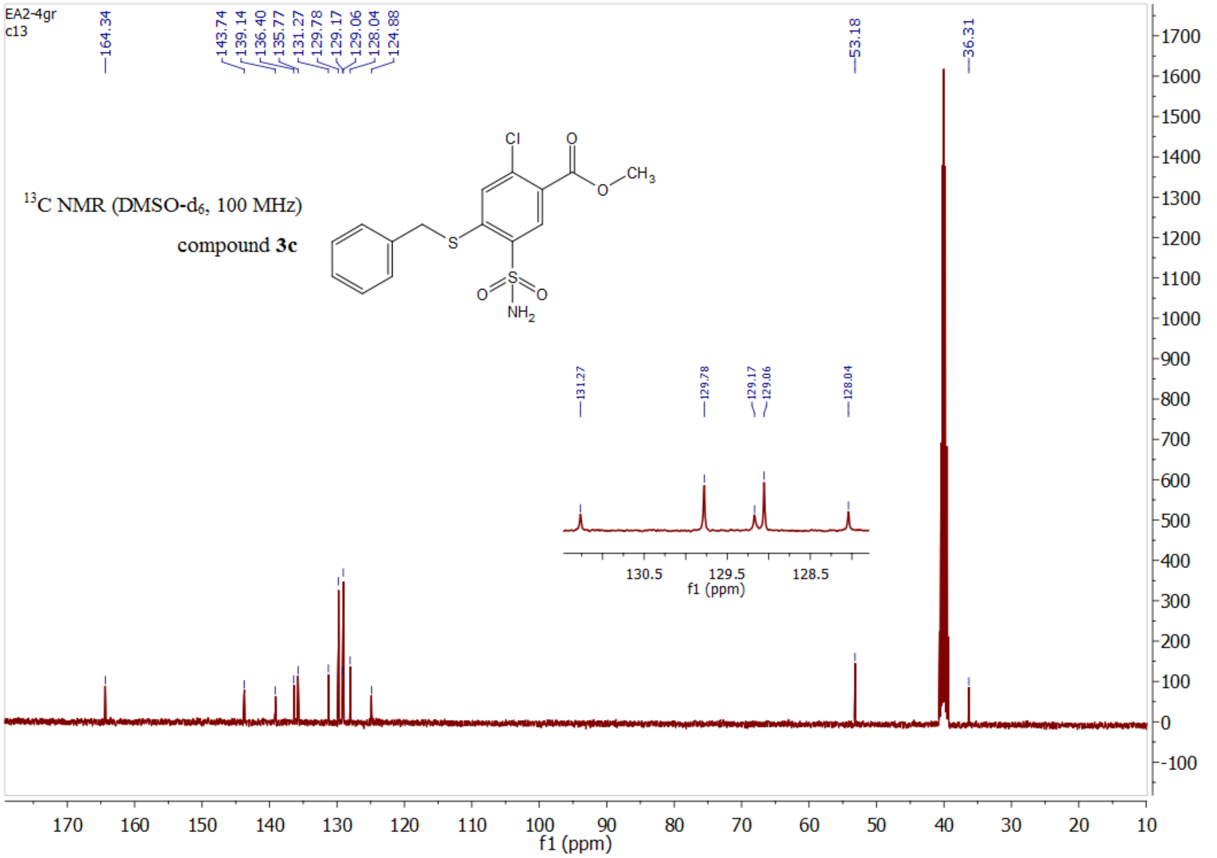
EA2-4gr
c13
164.34
143.74
139.14
136.40
135.77
131.27
129.78
129.17
129.06
128.04
124.88
53.18
36.31
13C NMR (DMSO-d6, 100 MHz)
compound 3c
Cl
O
O
CH3
S
O
S
O
NH2
131.27
129.78
129.17
129.06
128.04
130.5
129.5
128.5
f1 (ppm)
f1 (ppm)

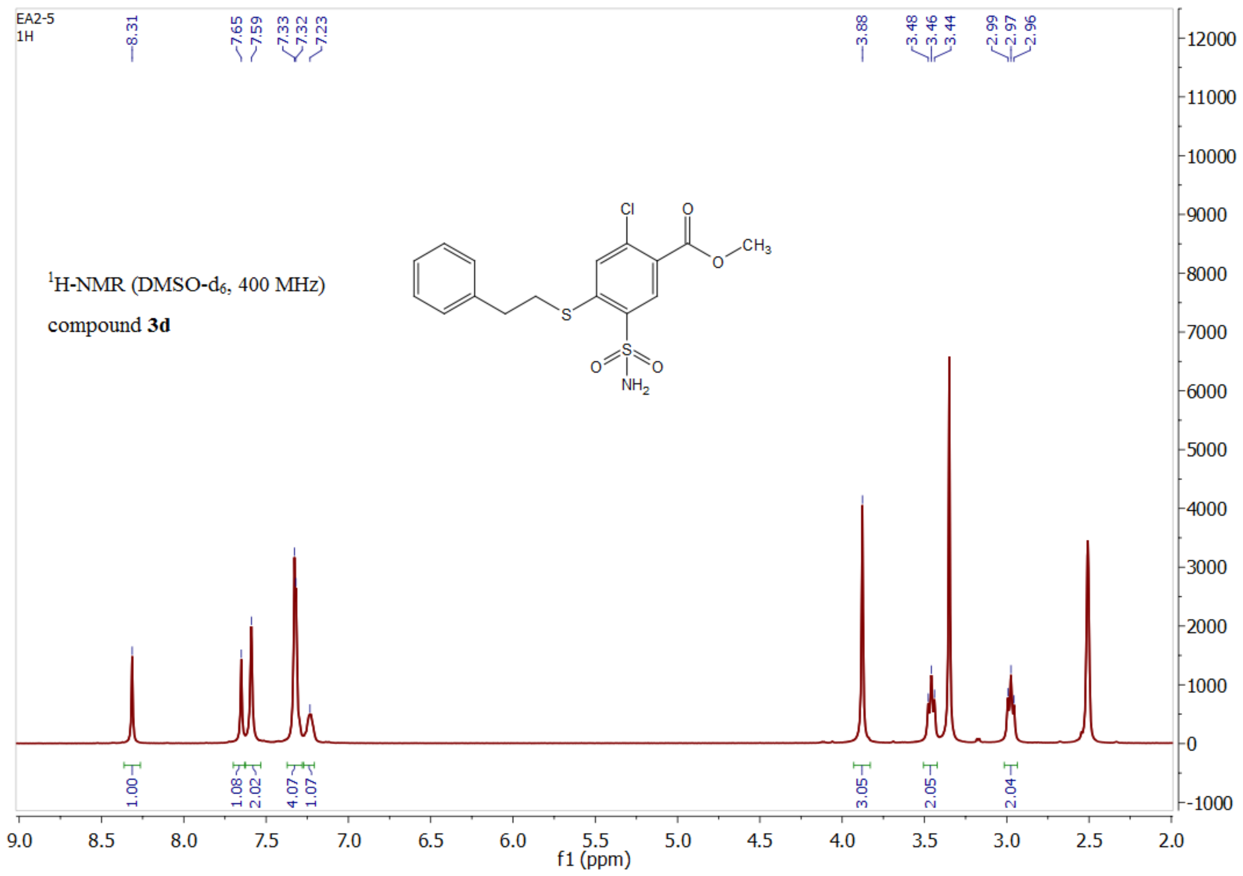

EA2-5
1H
^{1}H-NMR (DMSO-d6, 400 MHz)
compound **3d**

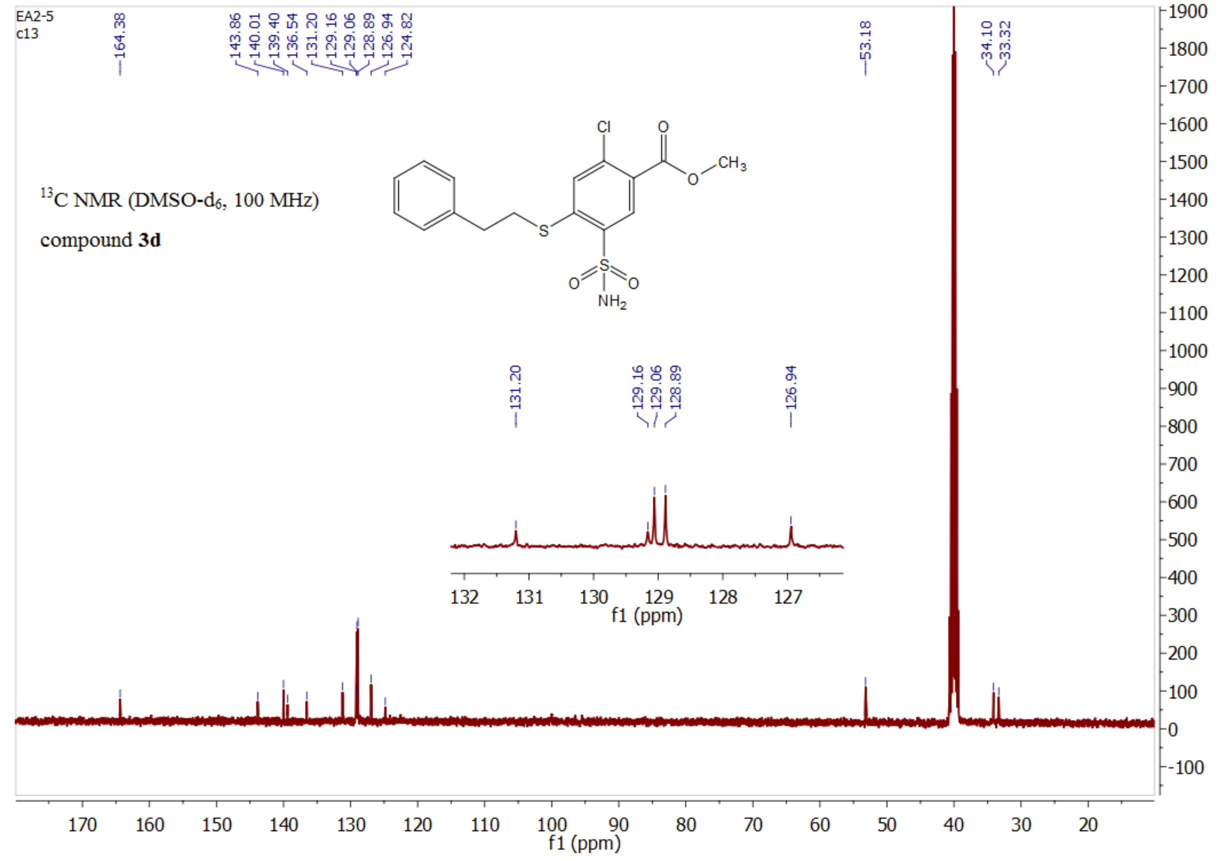

EA2-5
c13
^{13}C NMR (DMSO-d6, 100 MHz)
compound **3d**

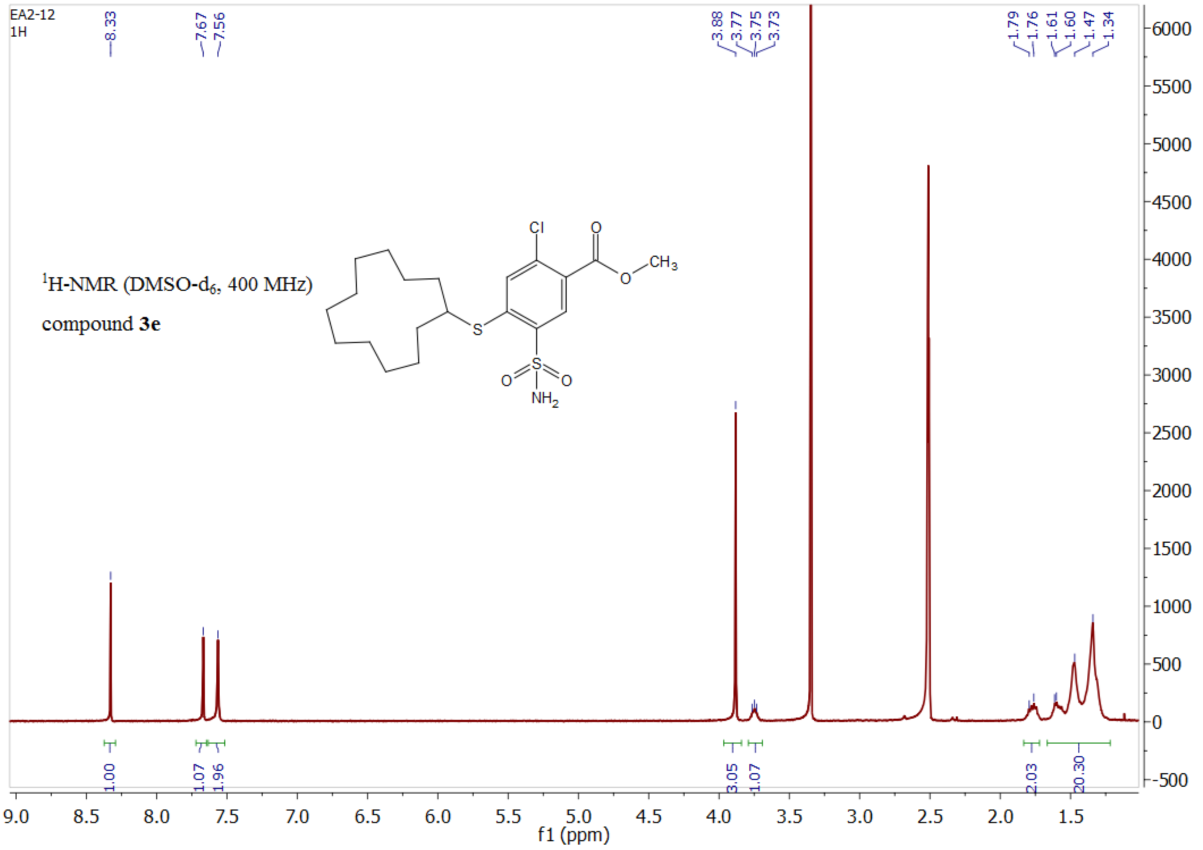

EA2-12
1H
8.33
7.67
7.56
3.88
3.77
3.75
3.73
1.79
1.76
1.61
1.60
1.47
1.34
^{1}H-NMR (DMSO-d6, 400 MHz)
compound 3e
Cl
O
CH3
O
S
O
S
O
NH2
1.00
1.07
1.96
3.05
1.07
2.03
20.30
f1 (ppm)

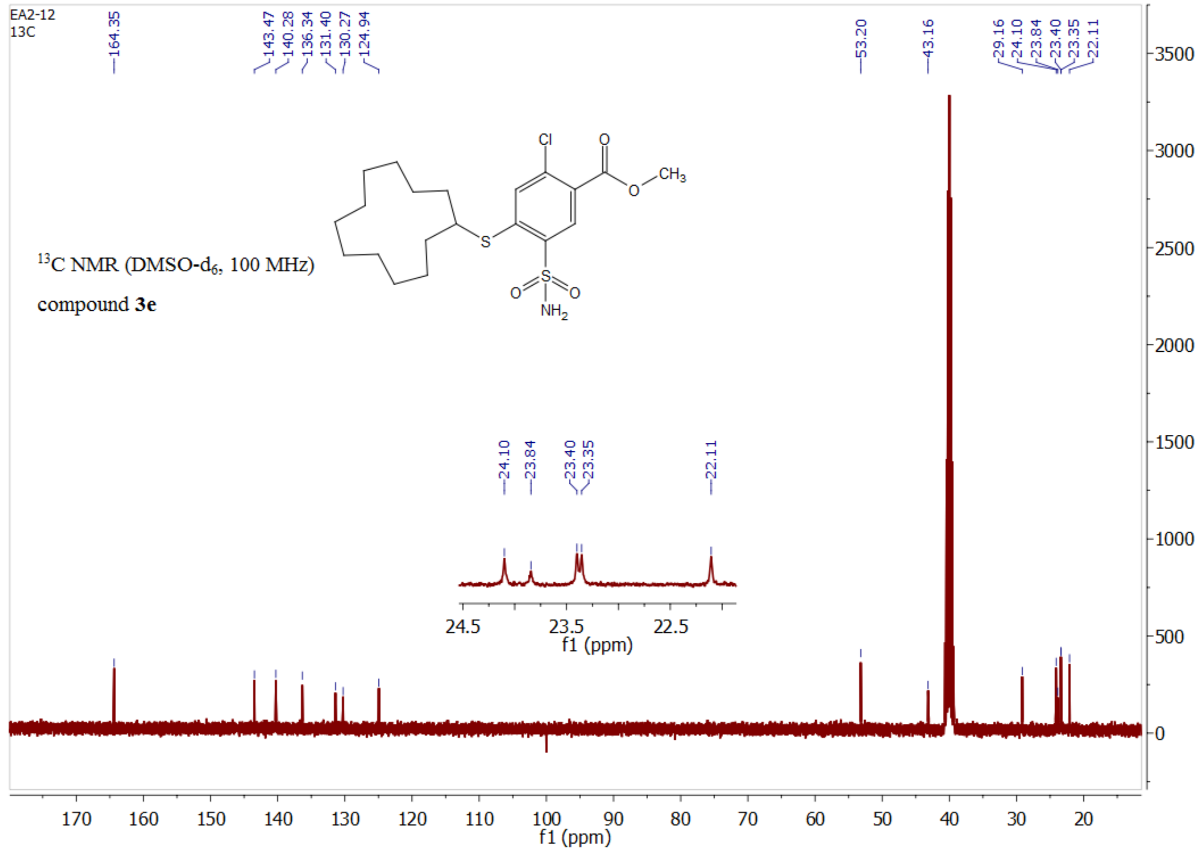

EA2-12
13C
164.35
143.47
140.28
136.34
131.40
130.27
124.94
53.20
43.16
29.16
24.10
23.84
23.40
23.35
22.11
^{13}C NMR (DMSO-d6, 100 MHz)
compound 3e
Cl
O
CH3
O
S
O
S
O
NH2
f1 (ppm)

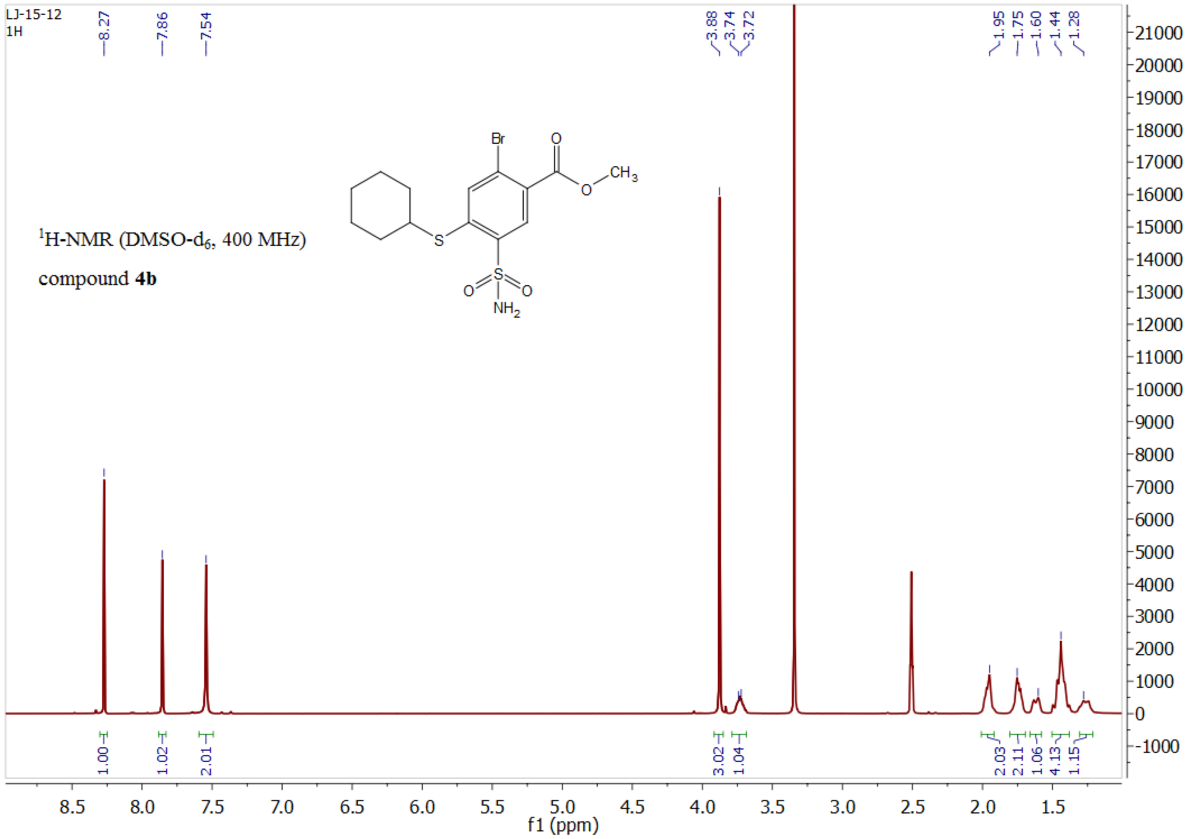
LJ-15-12
1H
8.27
7.86
7.54
3.88
3.74
3.72
1.95
1.75
1.60
1.44
1.28
Br
O
CH3
O
S
O
S
O
NH2
1H-NMR (DMSO-d6, 400 MHz)
compound 4b
1.00
1.02
2.01
3.02
1.04
2.03
2.11
1.06
4.13
1.15
f1 (ppm)

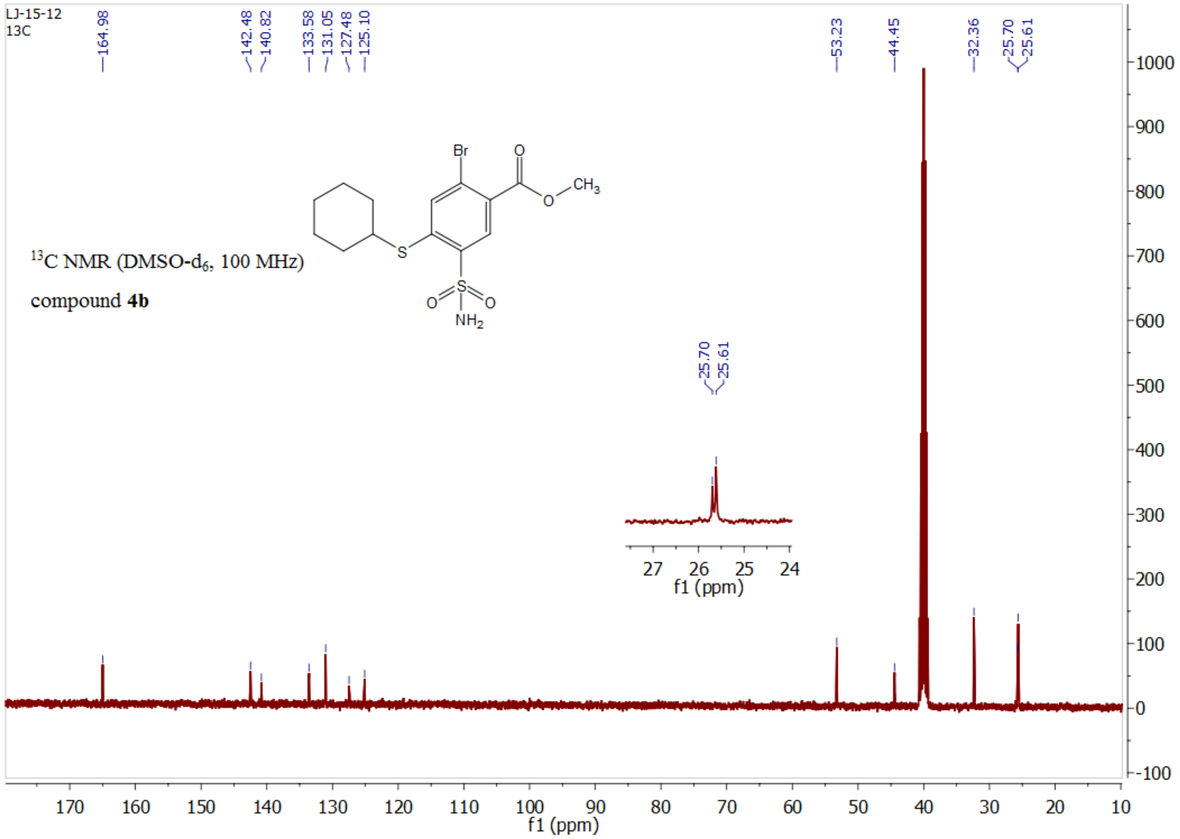
LJ-15-12
13C
164.98
142.48
140.82
133.58
131.05
127.48
125.10
53.23
44.45
32.36
25.70
25.61
Br
O
CH3
O
S
O
S
O
NH2
13C NMR (DMSO-d6, 100 MHz)
compound 4b
f1 (ppm)

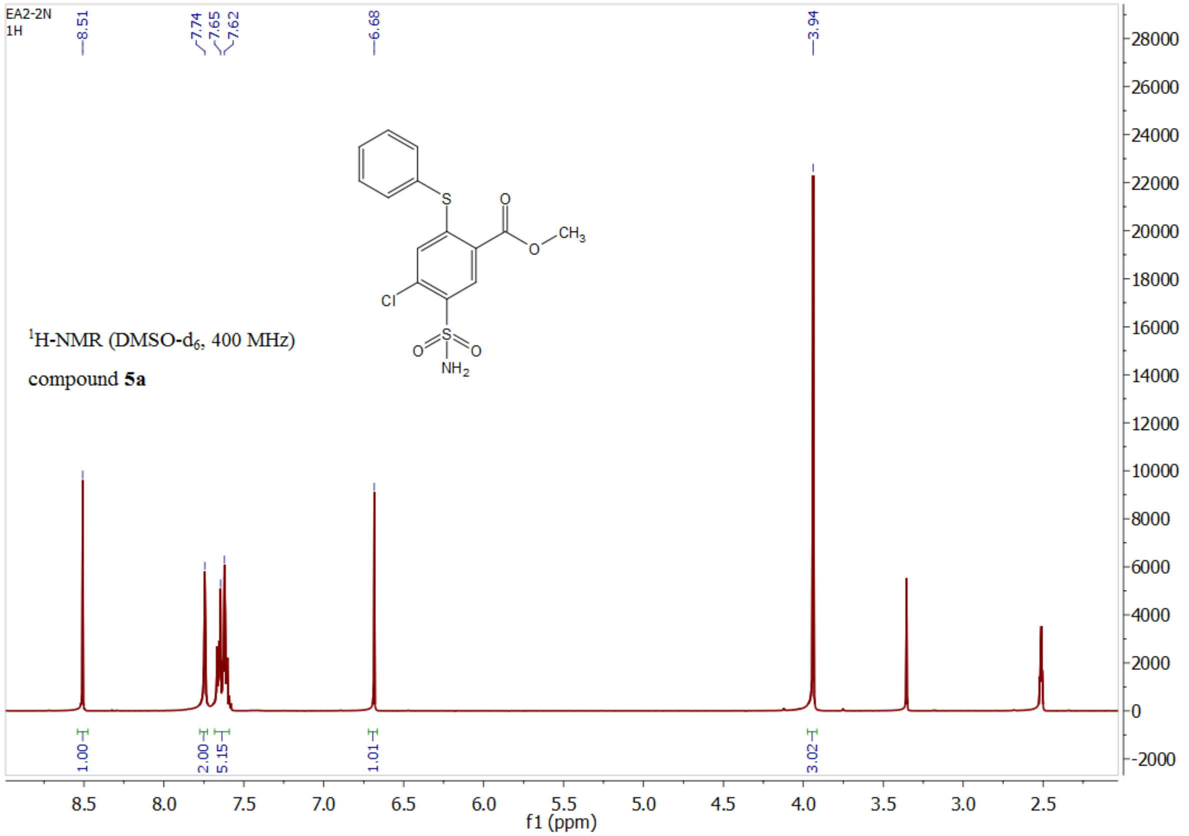

EA2-2N
1H
8.51
7.74
7.65
7.62
6.68
3.94
S
O
O
CH3
Cl
S
O
O
NH2
1H-NMR (DMSO-d6, 400 MHz)
compound 5a
1.00
2.00
5.15
1.01
3.02
f1 (ppm)

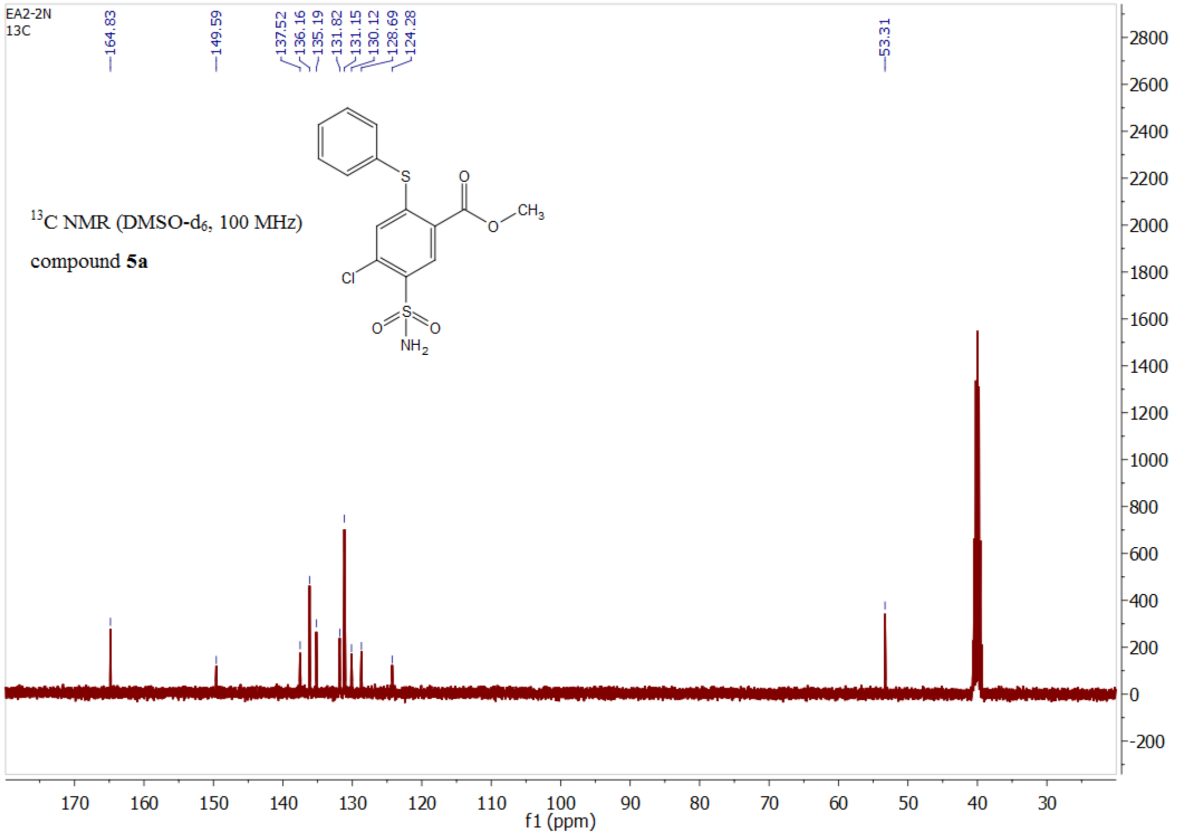

EA2-2N
13C
164.83
149.59
137.52
136.16
135.19
131.82
131.15
130.12
128.69
124.28
53.31
S
O
O
CH3
Cl
S
O
O
NH2
13C NMR (DMSO-d6, 100 MHz)
compound 5a
f1 (ppm)

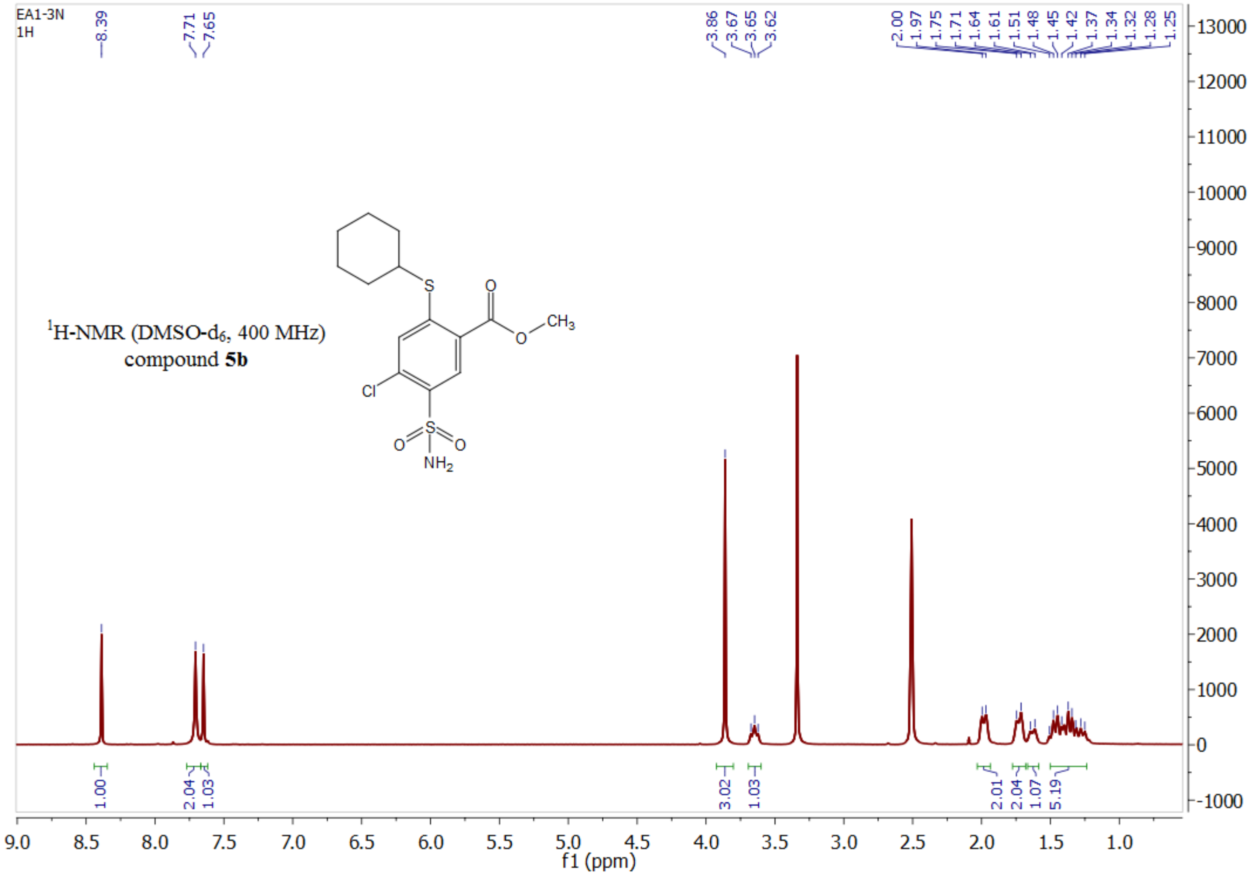
EA1-3N
1H
8.39
7.71
7.65
3.86
3.67
3.65
3.62
2.00
1.97
1.75
1.71
1.64
1.61
1.51
1.48
1.45
1.42
1.37
1.34
1.32
1.28
1.25
^{1}H-NMR (DMSO-d6, 400 MHz)
compound **5b**
1.00
2.04
1.03
3.02
1.03
2.01
2.04
1.07
5.19
f1 (ppm)

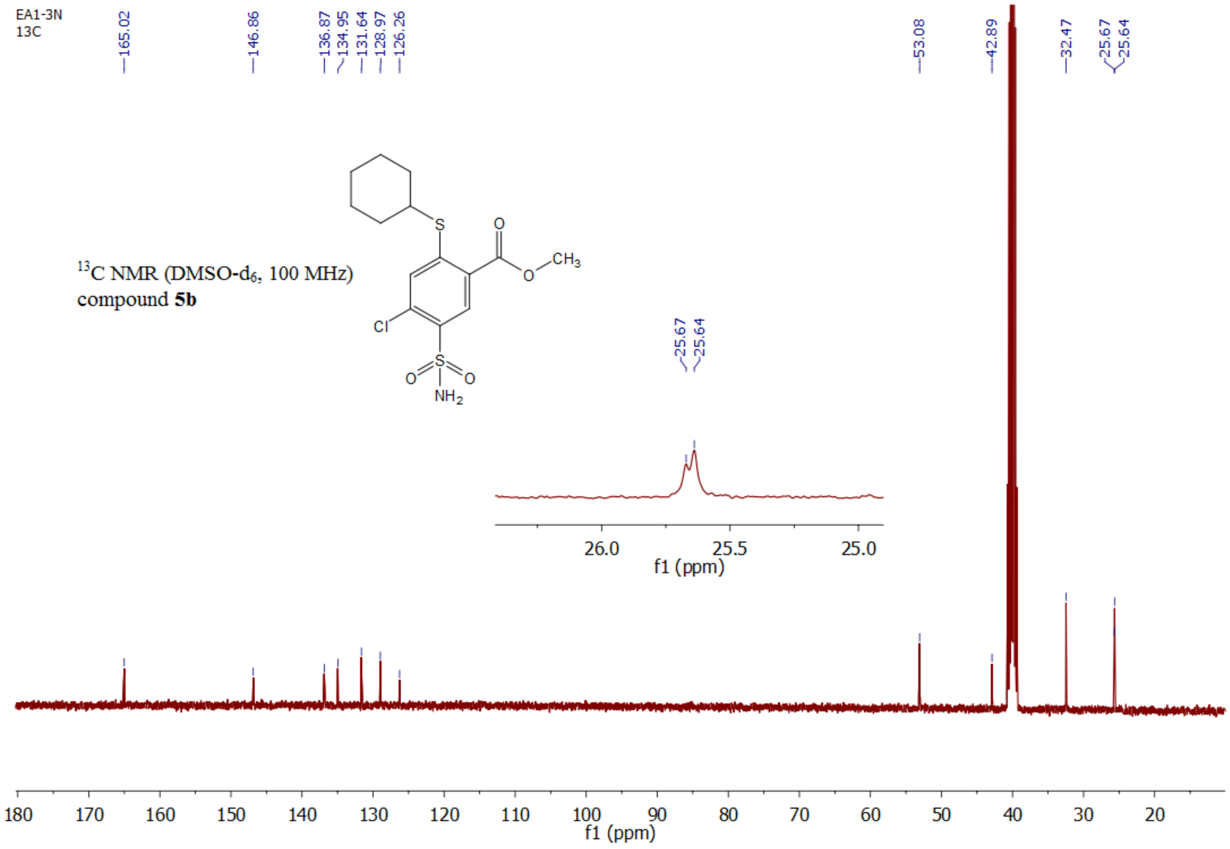
EA1-3N
13C
165.02
146.86
136.87
134.95
131.64
128.97
126.26
53.08
42.89
32.47
25.67
25.64
^{13}C NMR (DMSO-d6, 100 MHz)
compound **5b**
f1 (ppm)

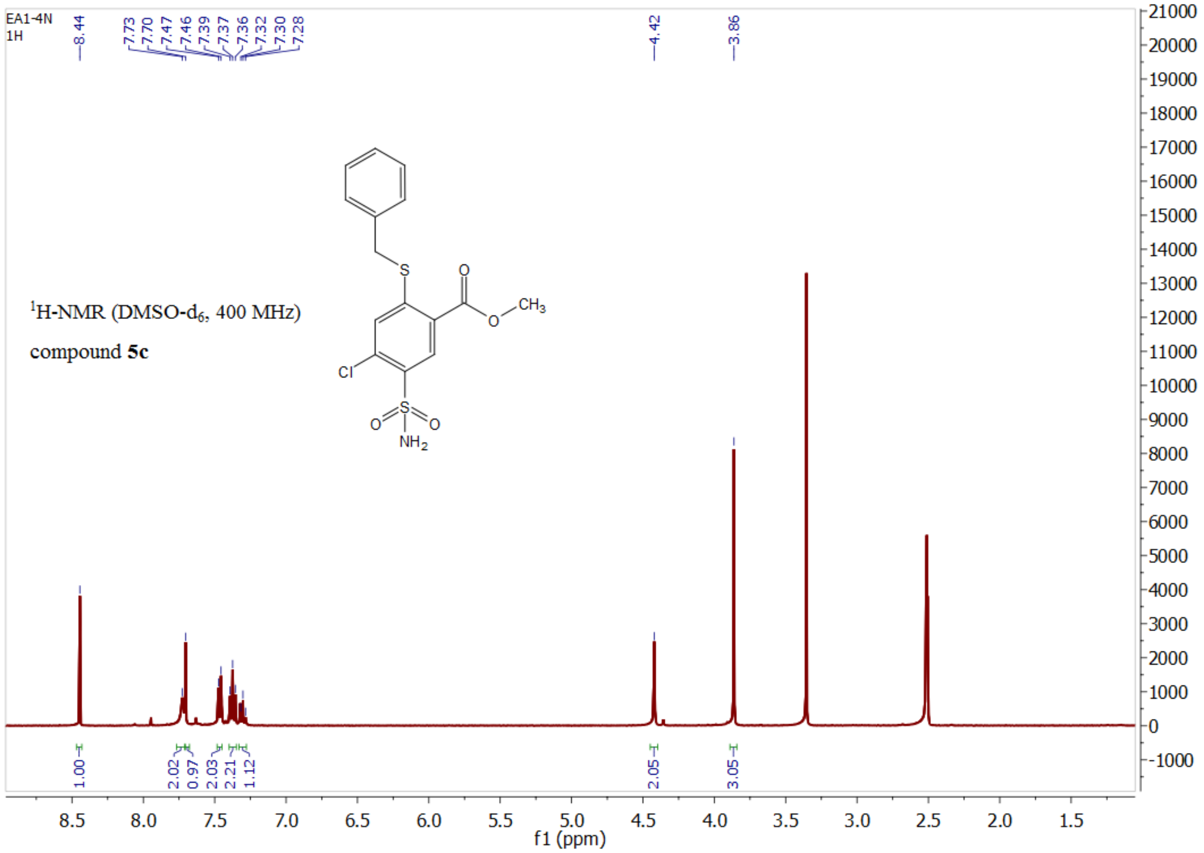

EA1-4N
1H
8.44
7.73
7.70
7.47
7.46
7.39
7.37
7.36
7.32
7.30
7.28
4.42
3.86
¹H-NMR (DMSO-d6, 400 MHz)
compound 5c
S
O
O
CH3
Cl
S
O
O
NH2
1.00
2.02
0.97
2.03
2.21
1.12
2.05
3.05
f1 (ppm)

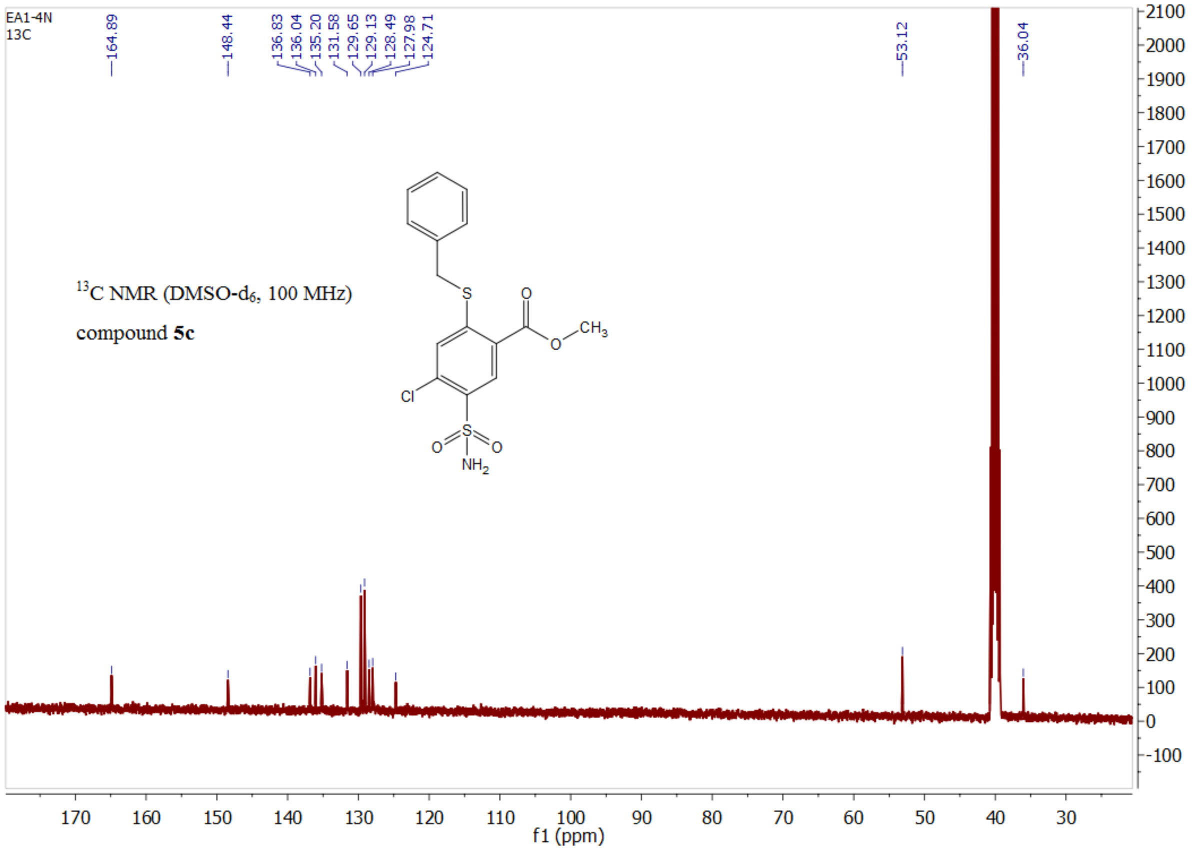

EA1-4N
13C
164.89
148.44
136.83
136.04
135.20
131.58
129.65
129.13
128.49
127.98
124.71
53.12
36.04
¹³C NMR (DMSO-d6, 100 MHz)
compound 5c
f1 (ppm)

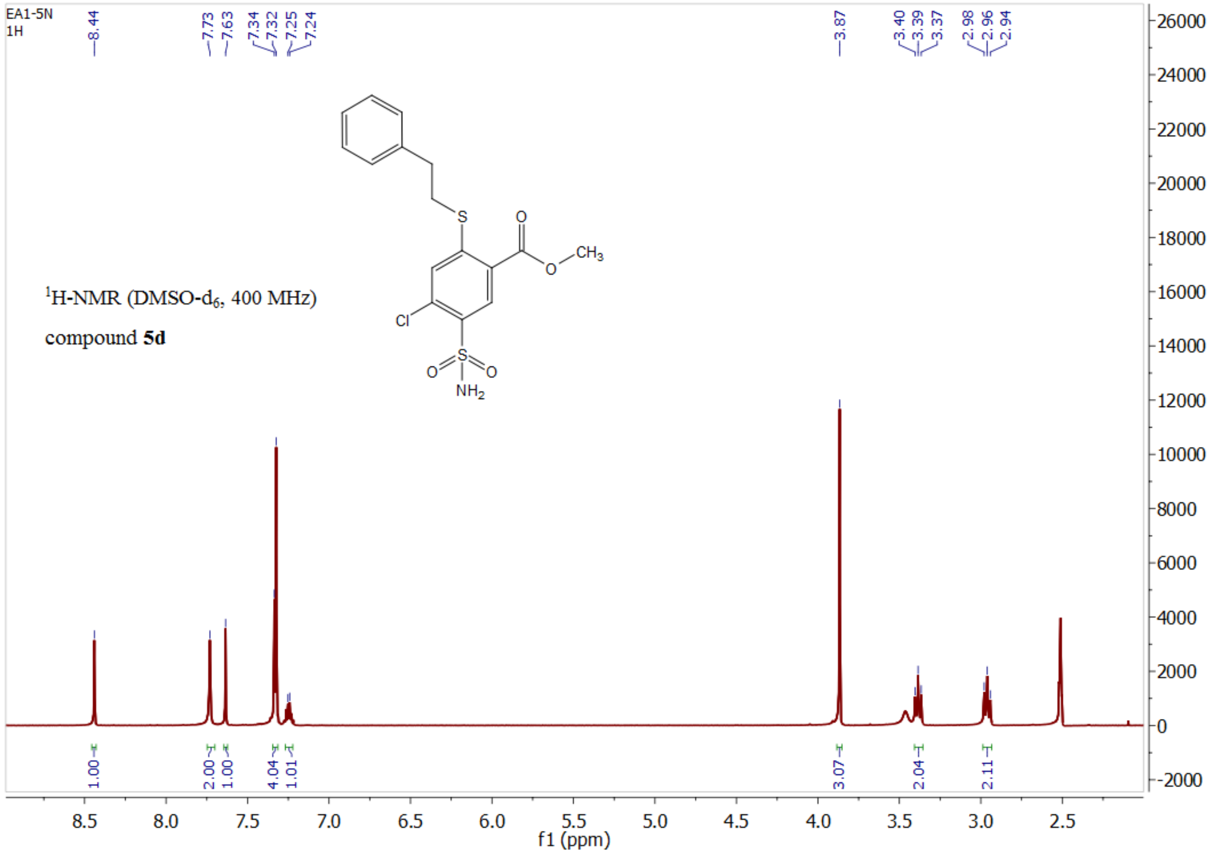
EA1-5N
1H
8.44
7.73
7.63
7.34
7.32
7.25
7.24
3.87
3.40
3.39
3.37
2.98
2.96
2.94
S
O
O
CH3
Cl
O
S
O
NH2
1H-NMR (DMSO-d6, 400 MHz)
compound 5d
1.00
2.00
1.00
4.04
1.01
3.07
2.04
2.11
f1 (ppm)

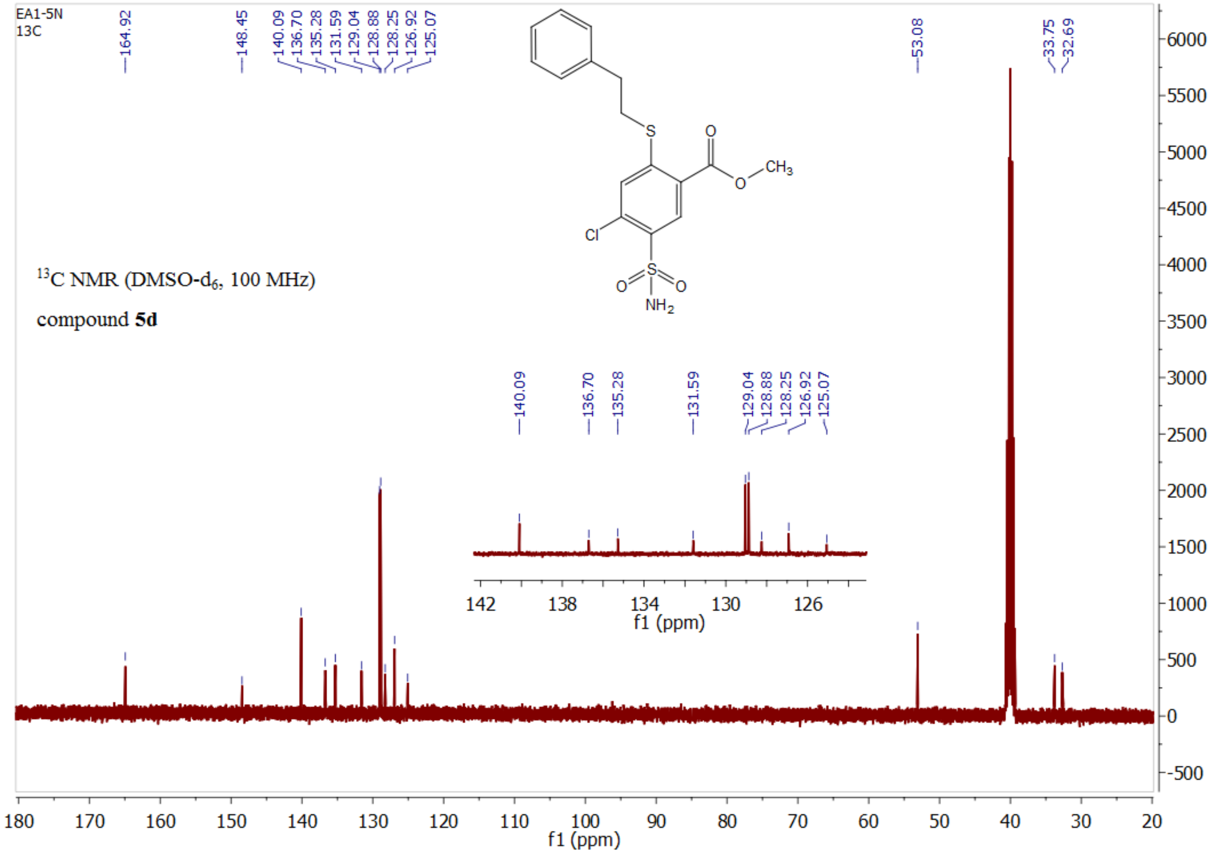
EA1-5N
13C
164.92
148.45
140.09
136.70
135.28
131.59
129.04
128.88
128.25
126.92
125.07
53.08
33.75
32.69
S
O
O
CH3
Cl
O
S
O
NH2
13C NMR (DMSO-d6, 100 MHz)
compound 5d
f1 (ppm)

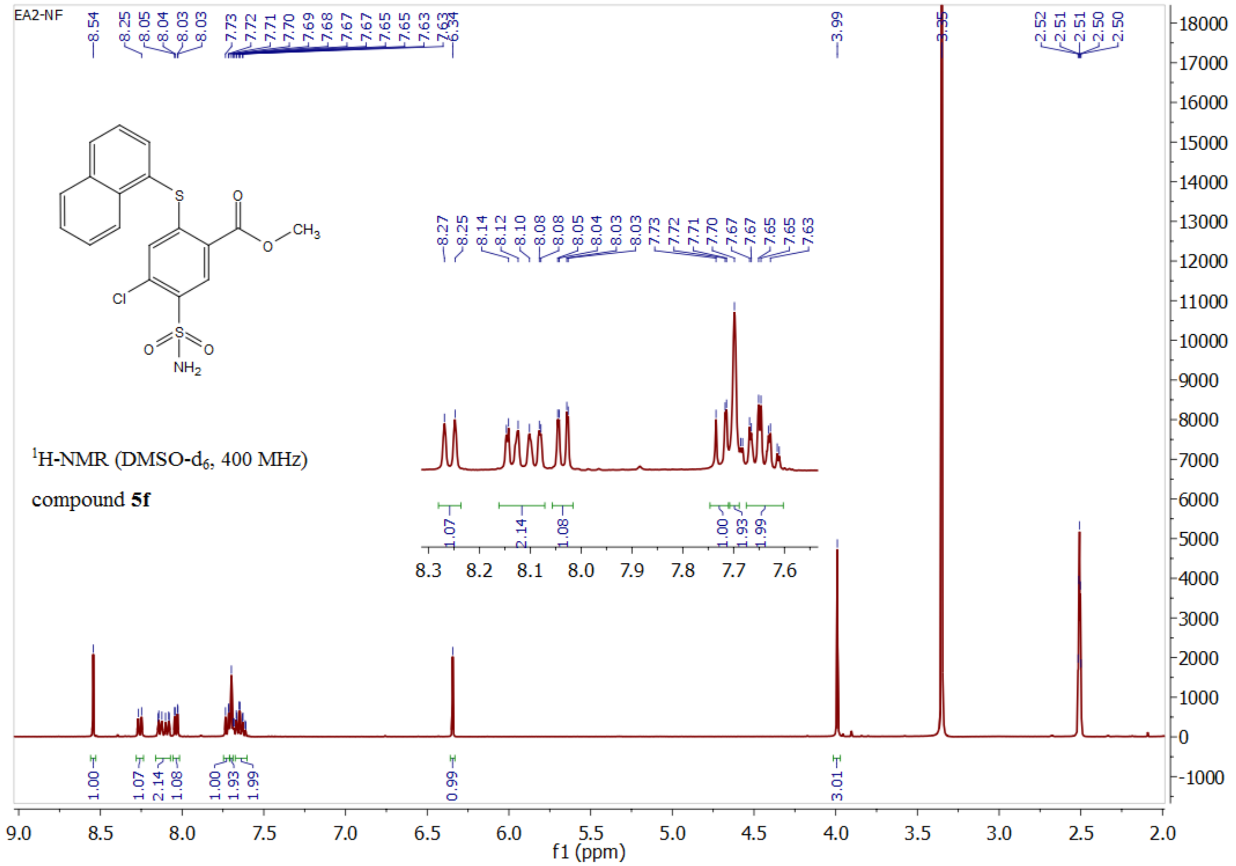
EA2-NF
1H-NMR (DMSO-d6, 400 MHz)
compound 5f
f1 (ppm)

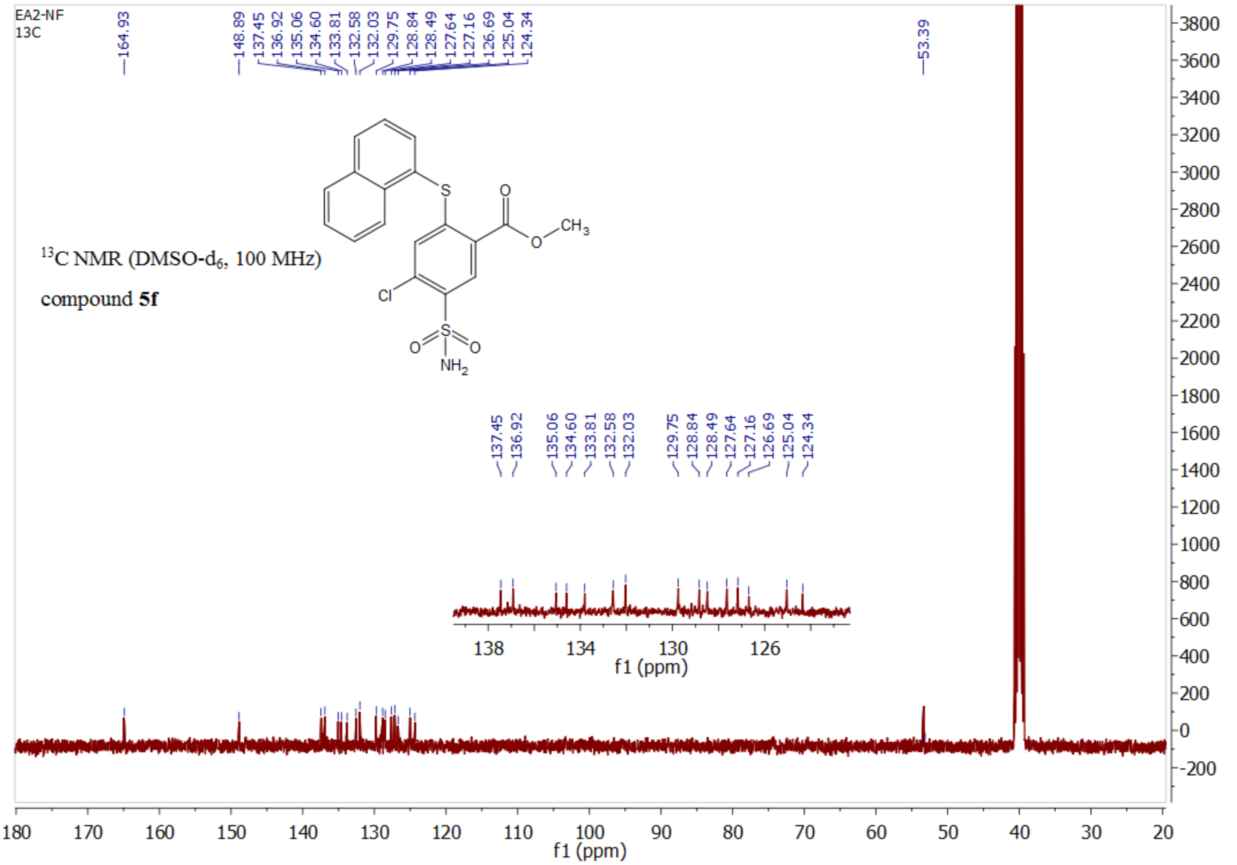
EA2-NF
13C
13C NMR (DMSO-d6, 100 MHz)
compound 5f
f1 (ppm)

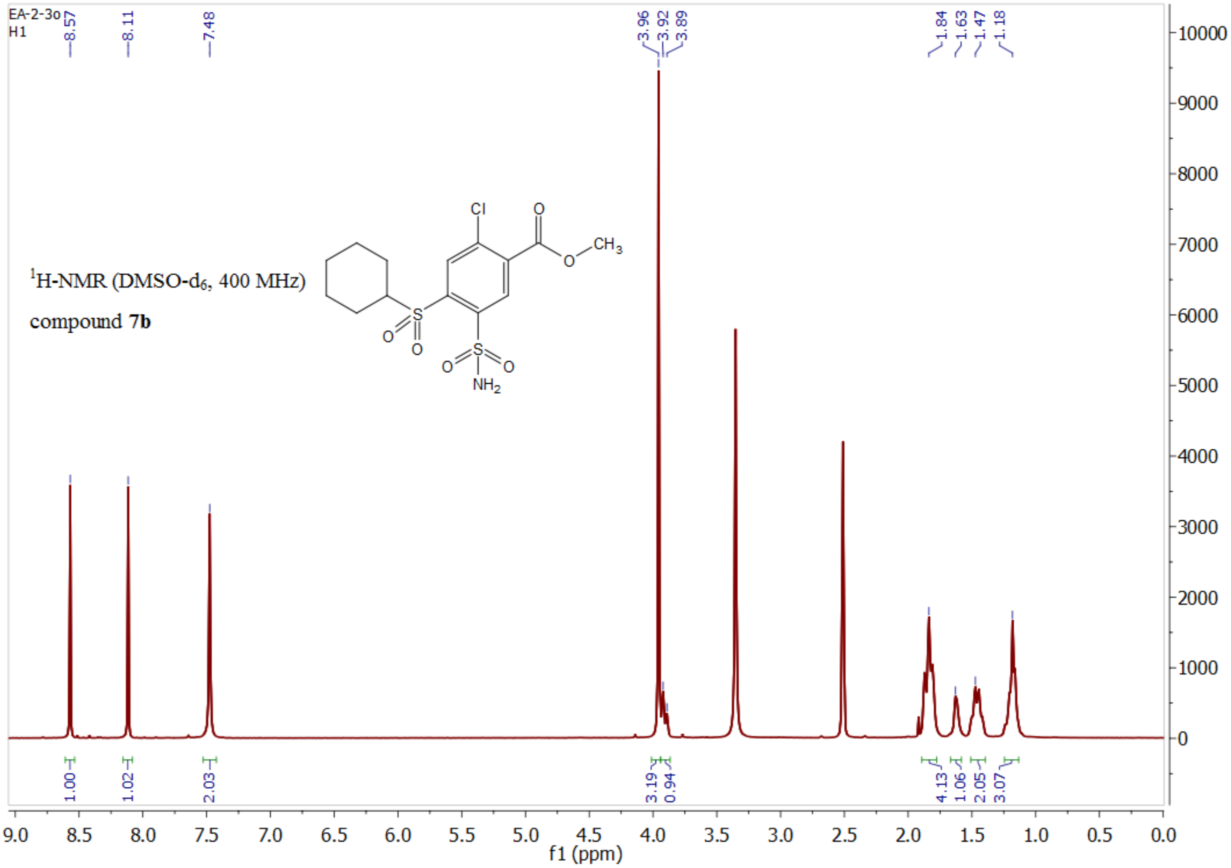
EA-2-3o
H1
8.57
8.11
7.48
3.96
3.92
3.89
1.84
1.63
1.47
1.18
^{1}H-NMR (DMSO-d$_6$, 400 MHz)
compound **7b**
Cl
O
O
CH3
S
O
O
O
S
O
NH2
1.00
1.02
2.03
3.19
0.94
4.13
1.06
2.05
3.07
f1 (ppm)

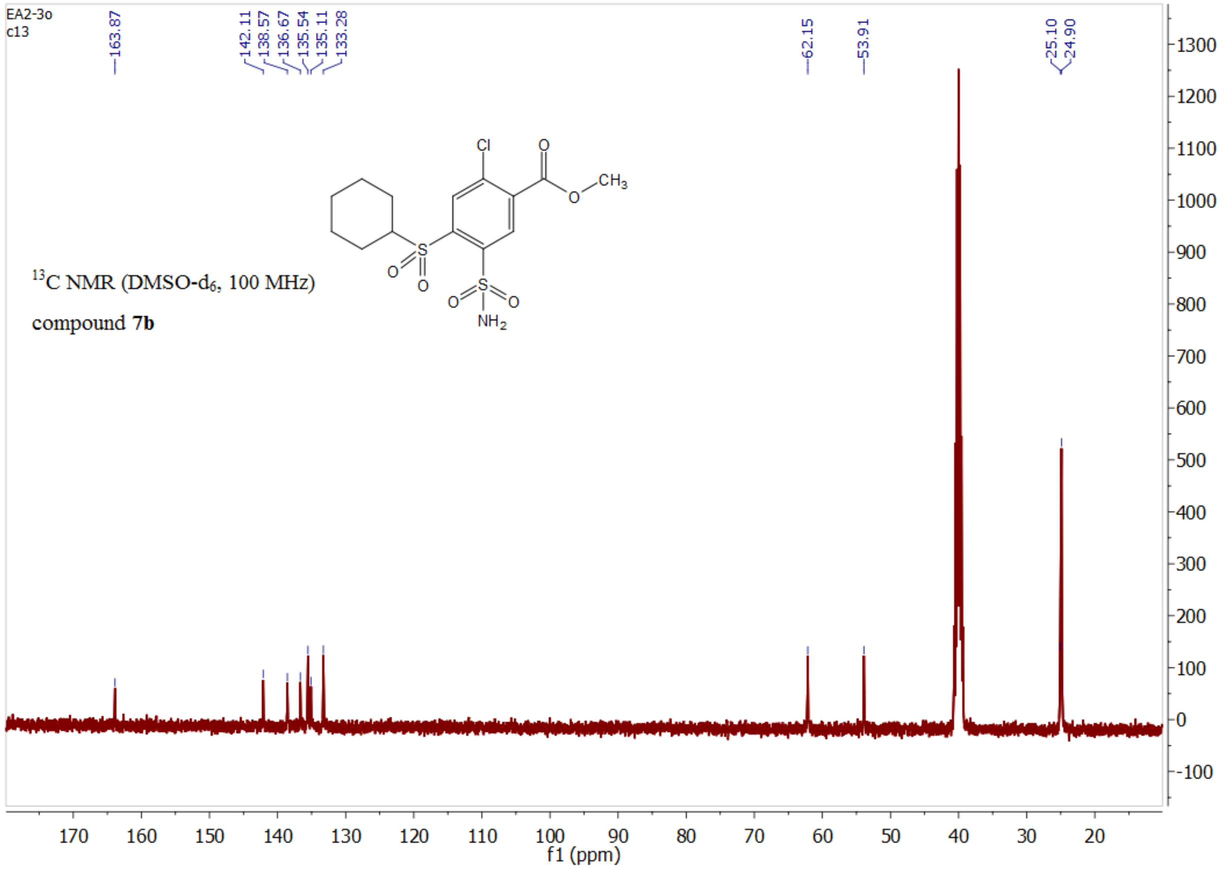
EA2-3o
c13
163.87
142.11
138.57
136.67
135.54
135.11
133.28
62.15
53.91
25.10
24.90
^{13}C NMR (DMSO-d$_6$, 100 MHz)
compound **7b**
Cl
O
O
CH3
S
O
O
O
S
O
NH2
f1 (ppm)

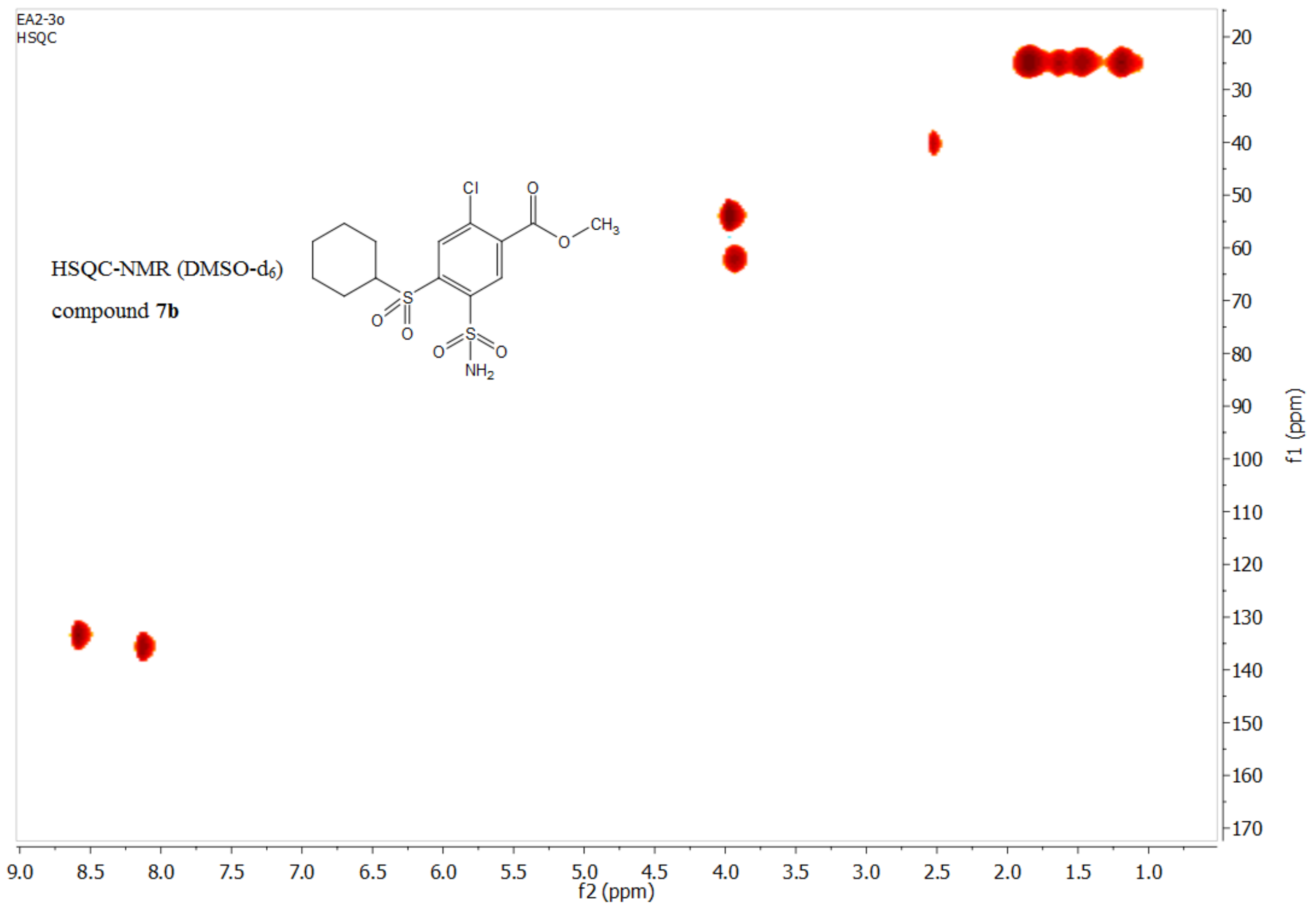
EA2-3o
HSQC
HSQC-NMR (DMSO-d6)
compound 7b
f1 (ppm)
f2 (ppm)

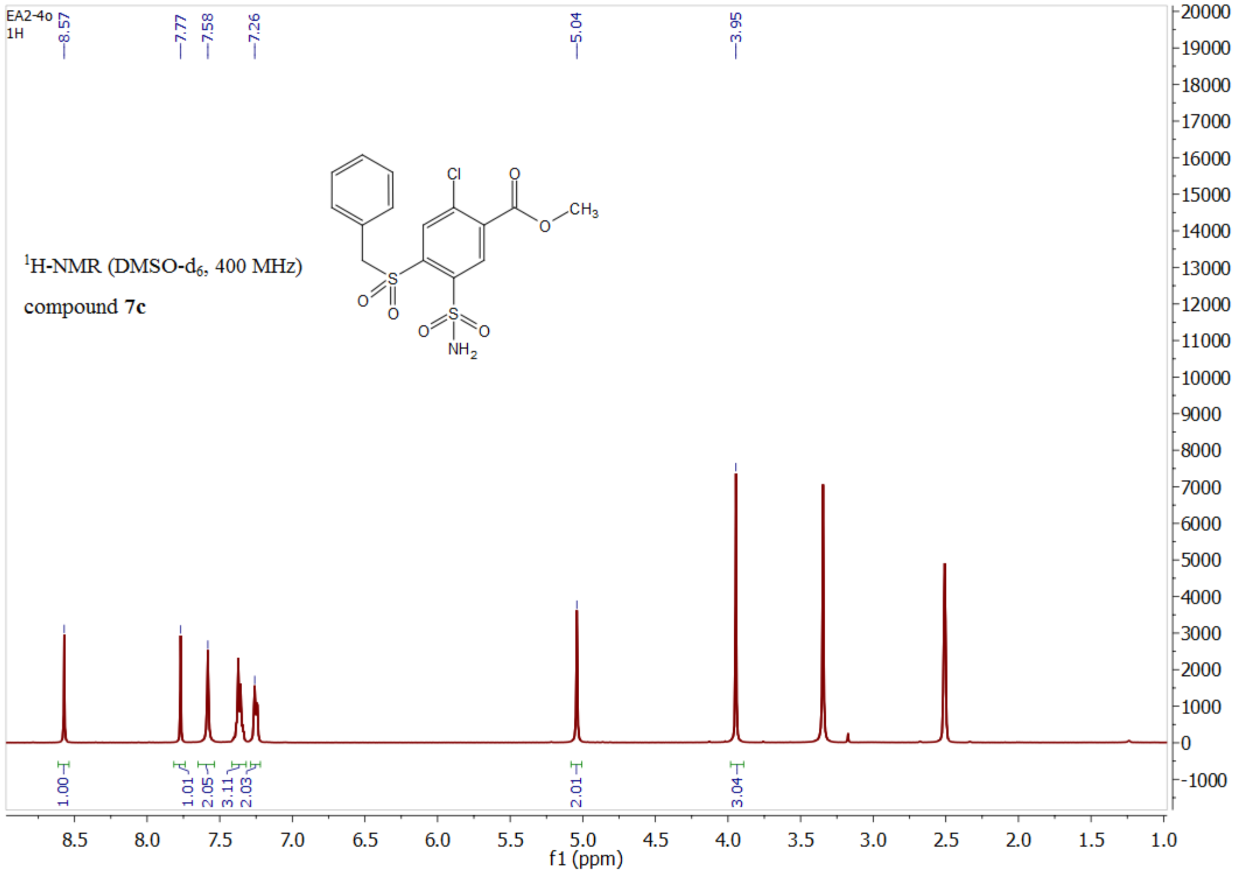
EA2-4o
1H
8.57
7.77
7.58
7.26
5.04
3.95
¹H-NMR (DMSO-d6, 400 MHz)
compound 7c
1.00
1.01
2.05
3.11
2.03
2.01
3.04
f1 (ppm)

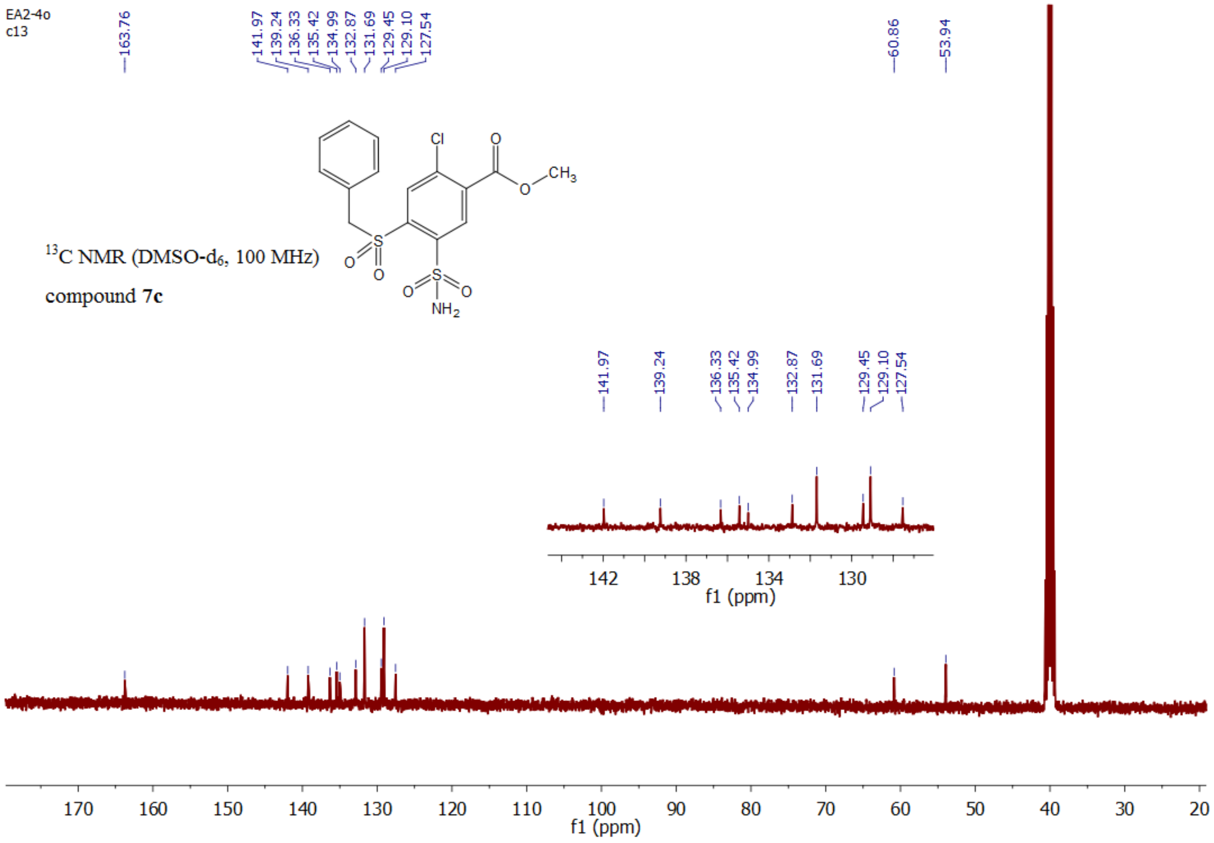
EA2-4o
c13
163.76
141.97
139.24
136.33
135.42
134.99
132.87
131.69
129.45
129.10
127.54
60.86
53.94
¹³C NMR (DMSO-d6, 100 MHz)
compound 7c
f1 (ppm)

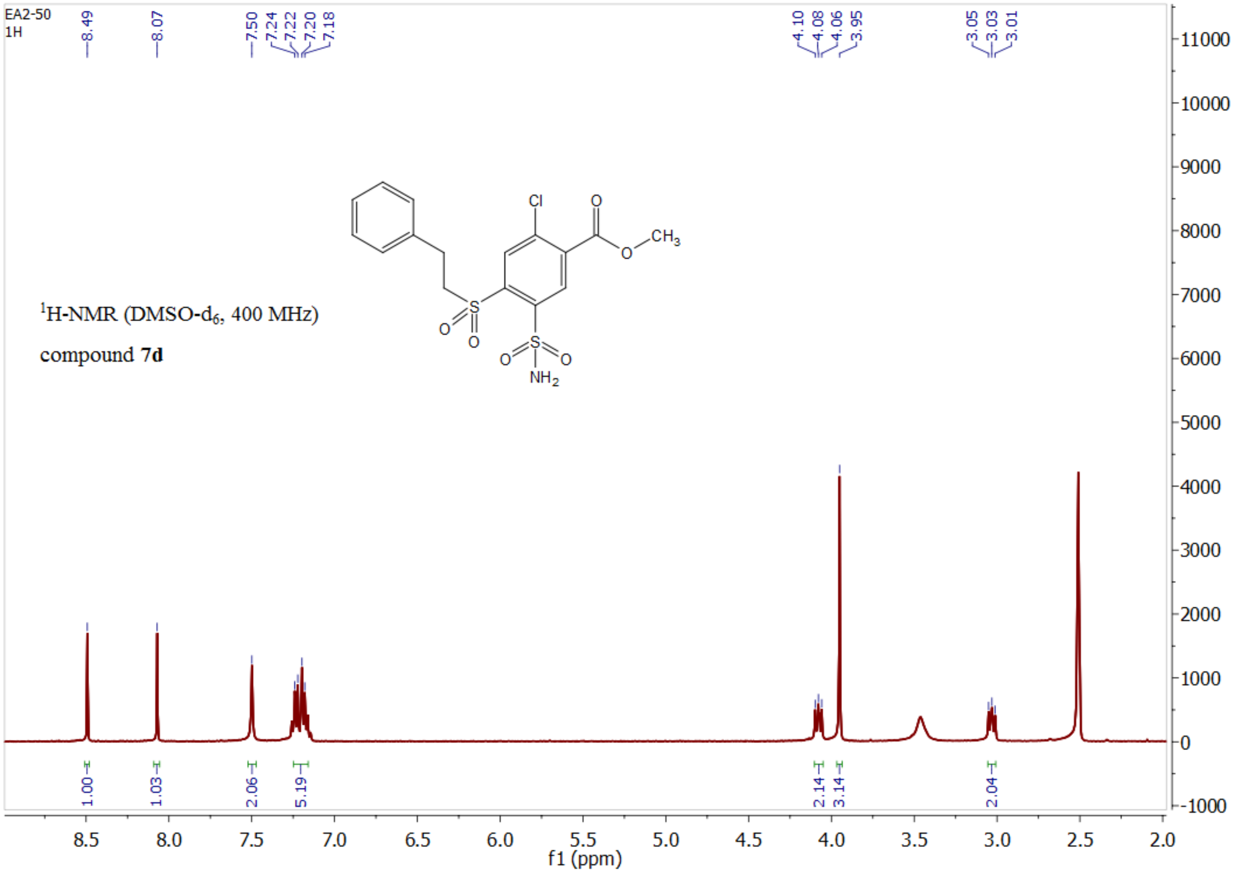
EA2-50
1H
8.49
8.07
7.50
7.24
7.22
7.20
7.18
4.10
4.08
4.06
3.95
3.05
3.03
3.01
^{1}H-NMR (DMSO-d$_6$, 400 MHz)
compound **7d**
1.00
1.03
2.06
5.19
2.14
3.14
2.04
f1 (ppm)

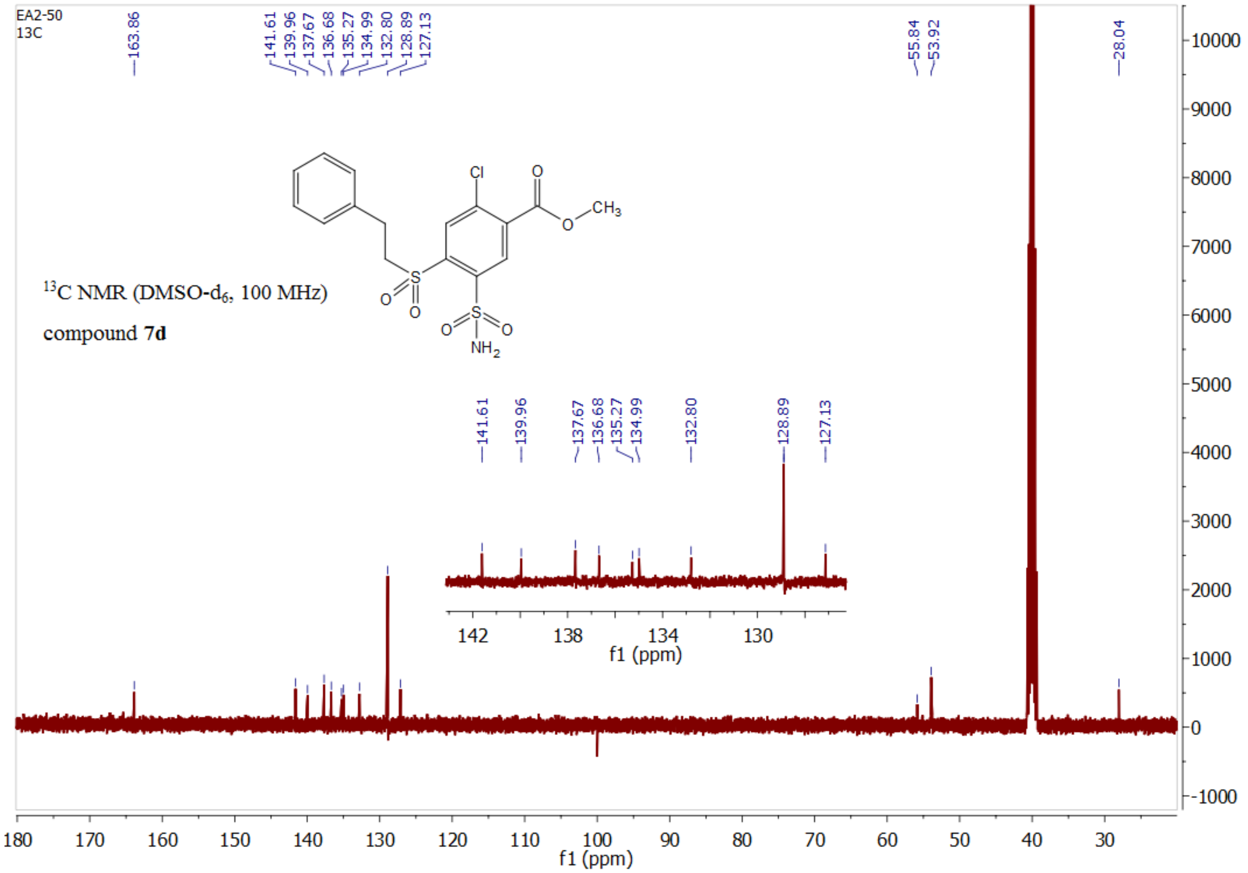
EA2-50
13C
163.86
141.61
139.96
137.67
136.68
135.27
134.99
132.80
128.89
127.13
55.84
53.92
28.04
^{13}C NMR (DMSO-d$_6$, 100 MHz)
compound **7d**
f1 (ppm)

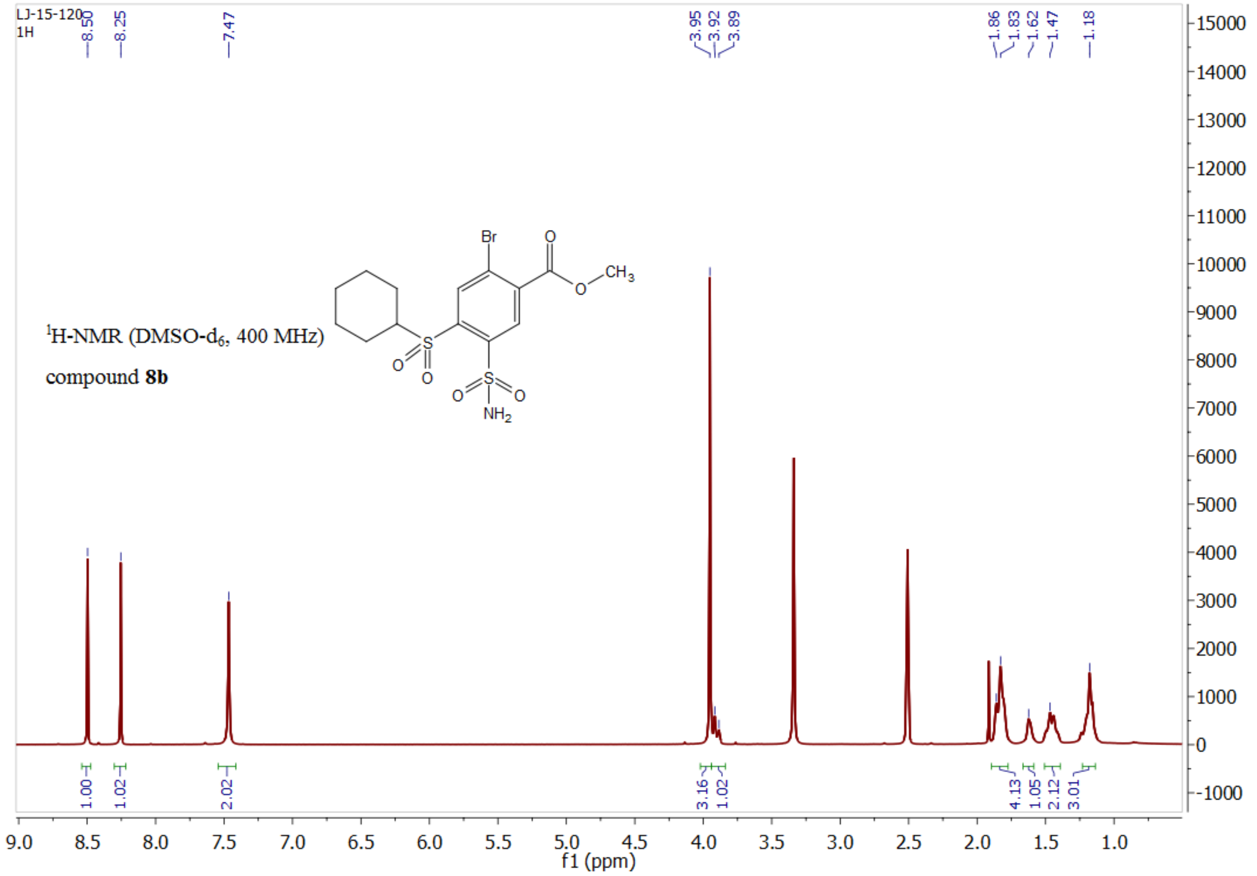

LJ-15-120
1H
8.50
8.25
7.47
3.95
3.92
3.89
1.86
1.83
1.62
1.47
1.18
^{1}H-NMR (DMSO-d$_6$, 400 MHz)
compound **8b**
1.00
1.02
2.02
3.16
1.02
4.13
1.05
2.12
3.01
f1 (ppm)

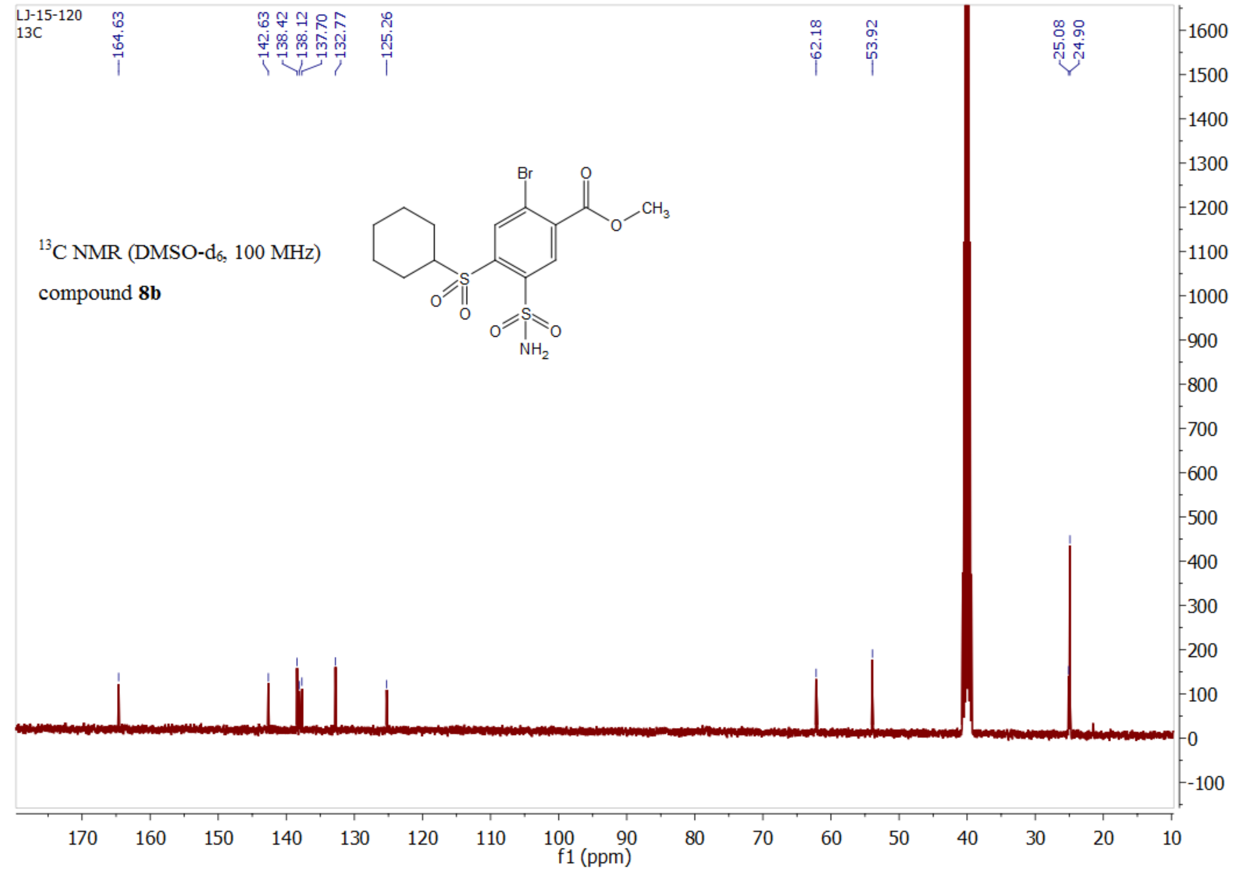

LJ-15-120
13C
164.63
142.63
138.42
138.12
137.70
132.77
125.26
62.18
53.92
25.08
24.90
^{13}C NMR (DMSO-d$_6$, 100 MHz)
compound **8b**
f1 (ppm)

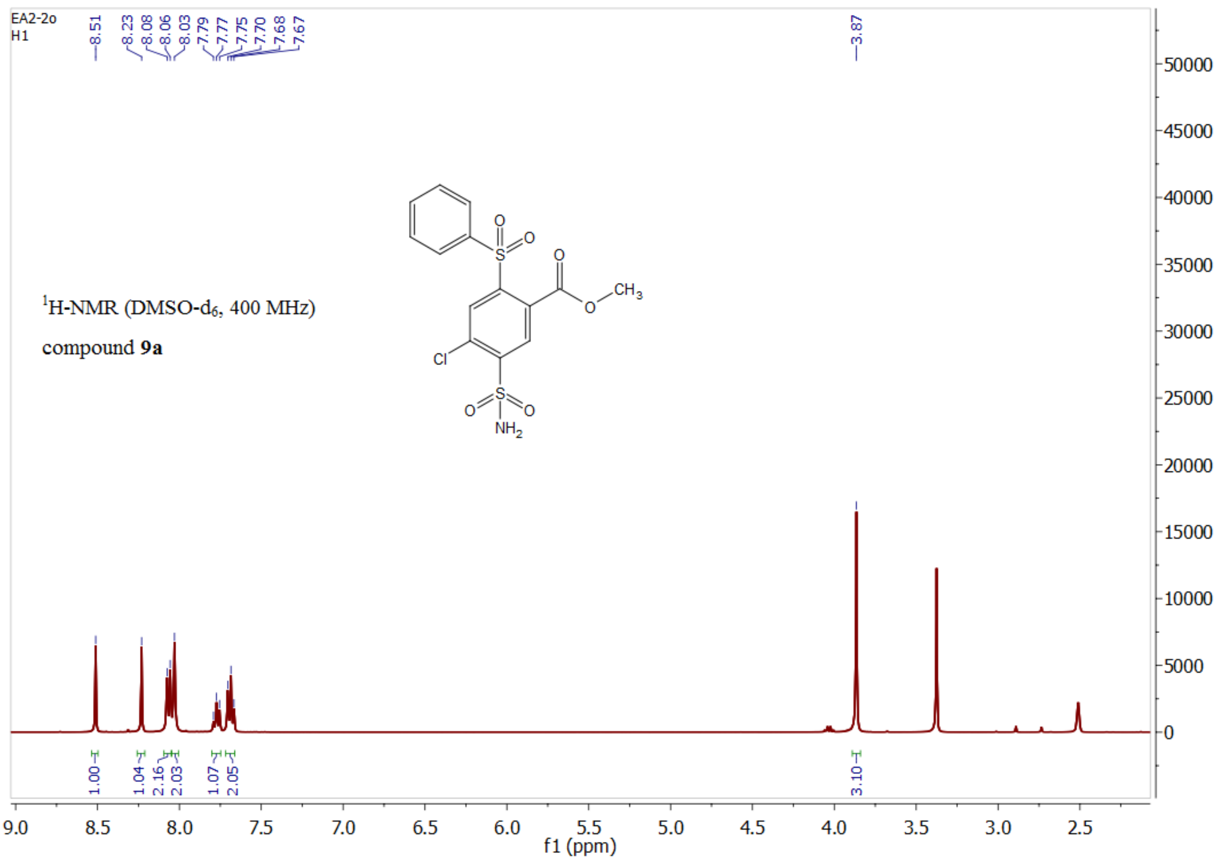
EA2-2o
H1
8.51
8.23
8.08
8.06
8.03
7.79
7.77
7.75
7.70
7.68
7.67
3.87
^{1}H-NMR (DMSO-d$_6$, 400 MHz)
compound **9a**
O
O
S
O
O
CH$_3$
Cl
O
S
O
NH$_2$
1.00
1.04
2.16
2.03
1.07
2.05
3.10
f1 (ppm)

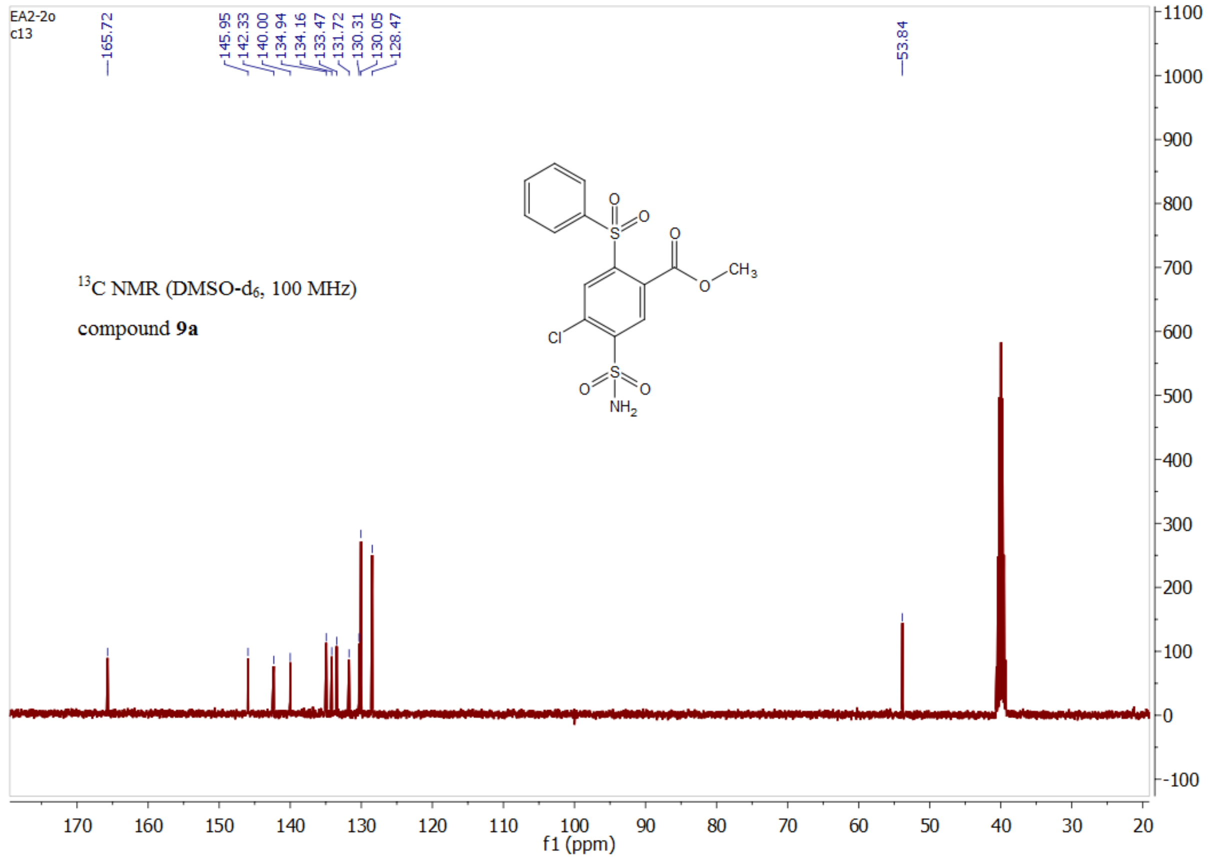
EA2-2o
c13
165.72
145.95
142.33
140.00
134.94
134.16
133.47
131.72
130.31
130.05
128.47
53.84
^{13}C NMR (DMSO-d$_6$, 100 MHz)
compound **9a**
O
O
S
O
O
CH$_3$
Cl
O
S
O
NH$_2$
f1 (ppm)

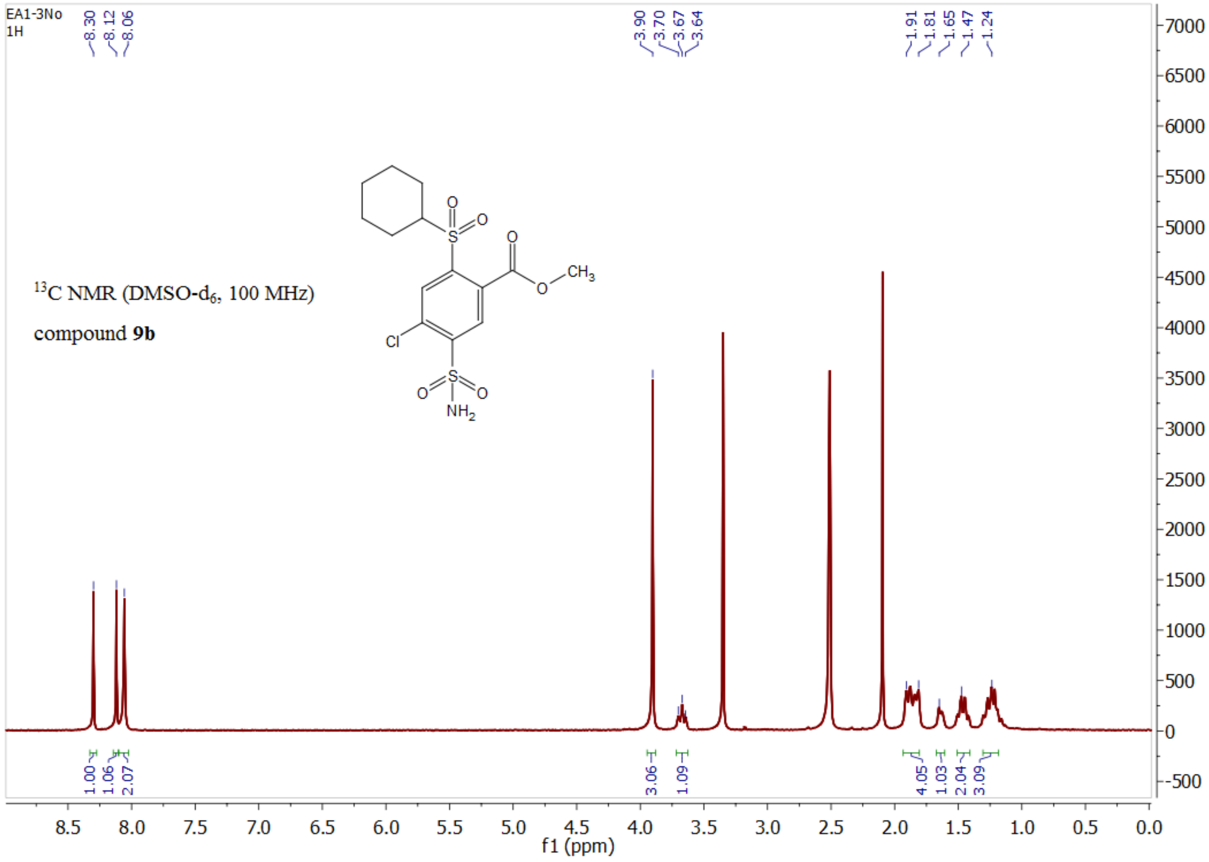
EA1-3No
1H
^{13}C NMR (DMSO-d$_6$, 100 MHz)
compound **9b**
8.30
8.12
8.06
3.90
3.70
3.67
3.64
1.91
1.81
1.65
1.47
1.24
1.00
1.06
2.07
3.06
1.09
4.05
1.03
2.04
3.09
f1 (ppm)

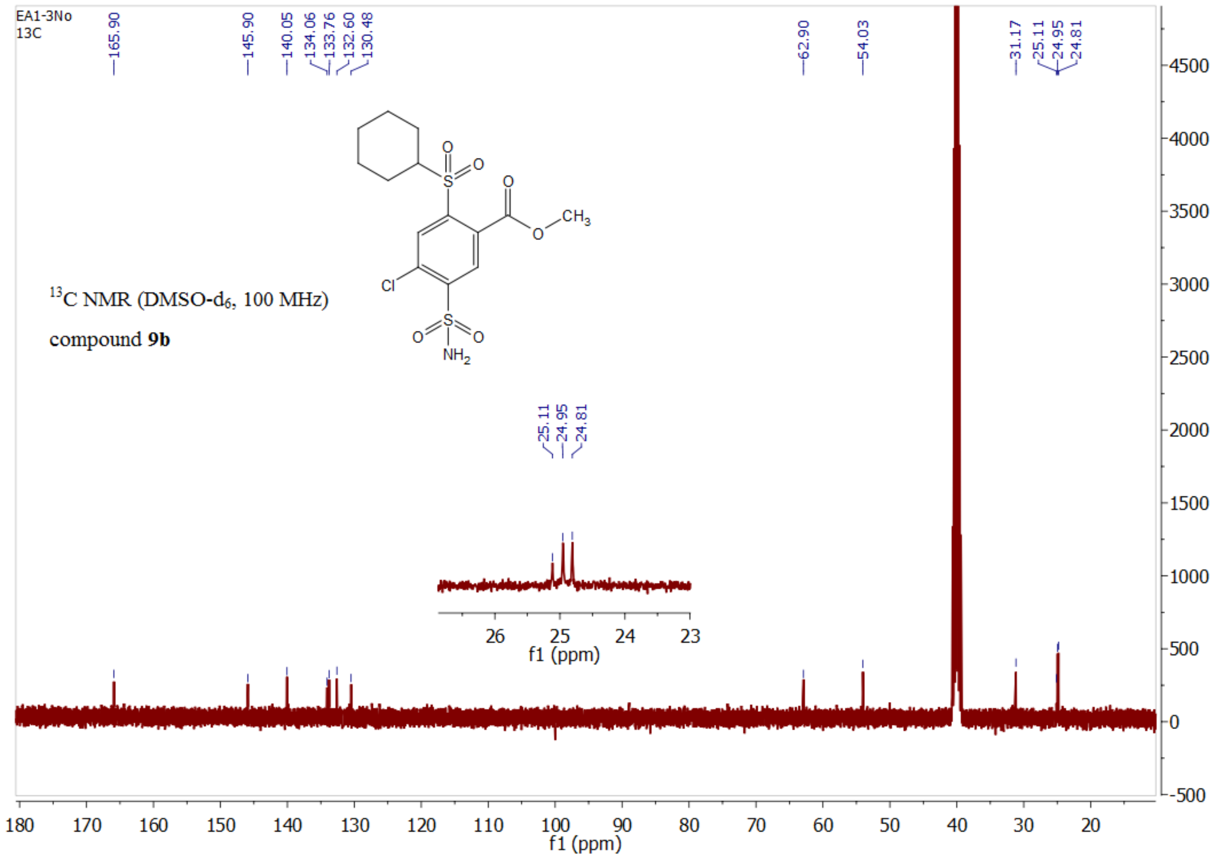
EA1-3No
13C
^{13}C NMR (DMSO-d$_6$, 100 MHz)
compound **9b**
165.90
145.90
140.05
134.06
133.76
132.60
130.48
62.90
54.03
31.17
25.11
24.95
24.81
f1 (ppm)

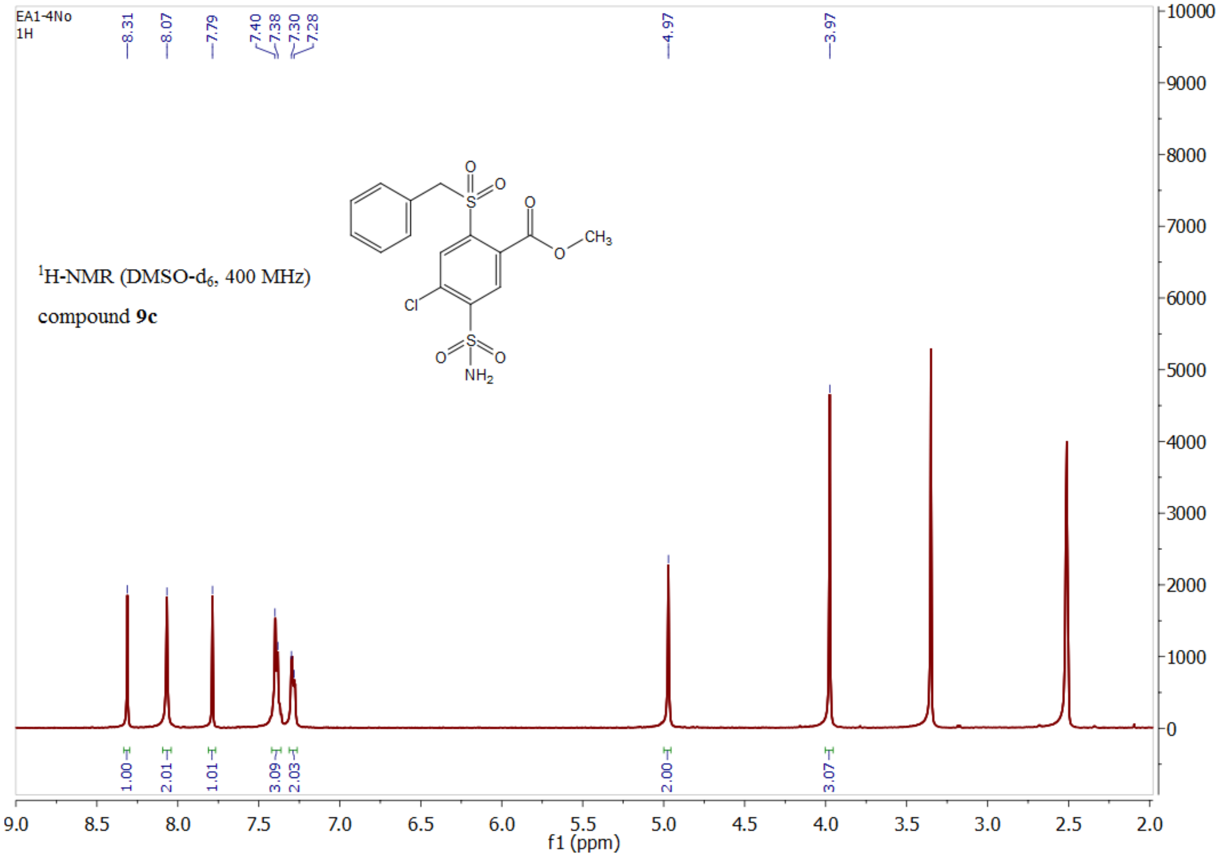
EA1-4No
1H
8.31
8.07
7.79
7.40
7.38
7.30
7.28
4.97
3.97
1H-NMR (DMSO-d6, 400 MHz)
compound 9c
Cl
NH2
CH3
1.00
2.01
1.01
3.09
2.03
2.00
3.07
f1 (ppm)

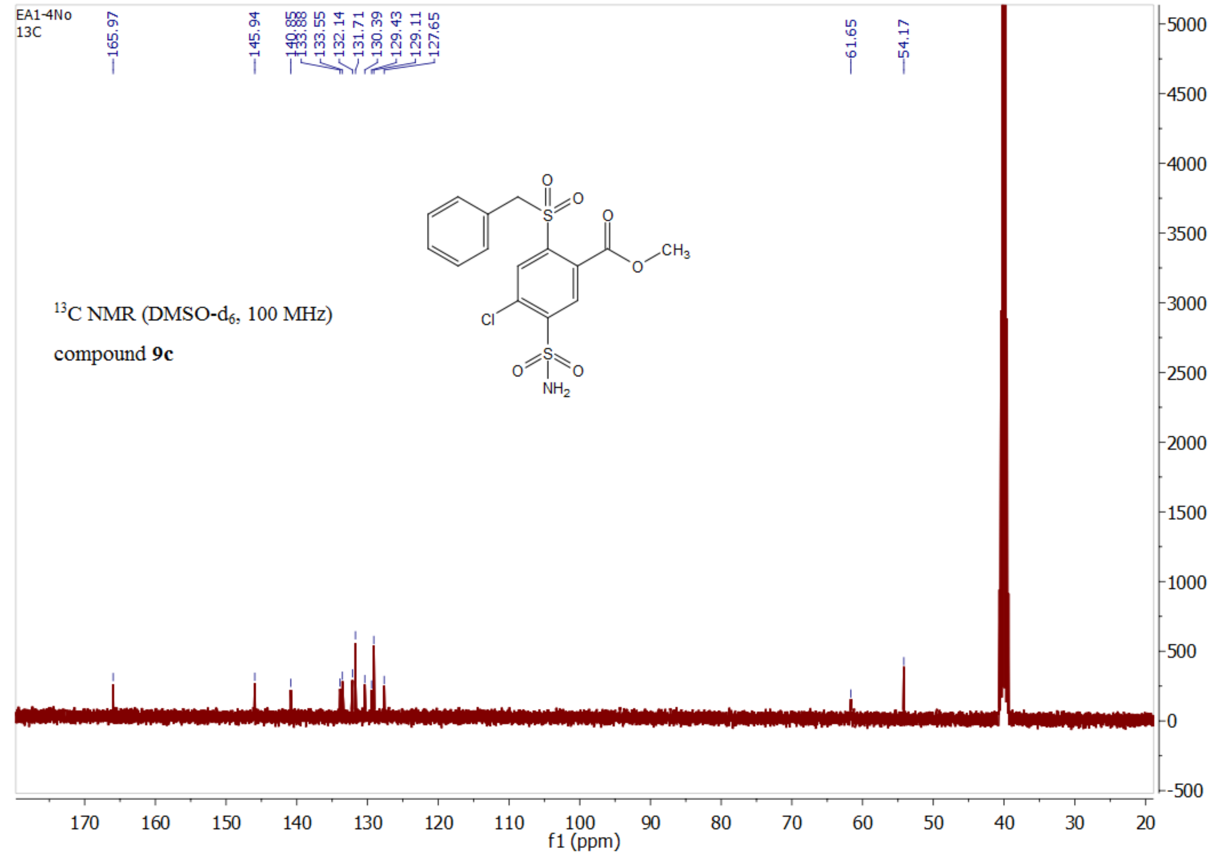
EA1-4No
13C
165.97
145.94
140.85
133.88
133.55
132.14
131.71
130.39
129.43
129.11
127.65
61.65
54.17
13C NMR (DMSO-d6, 100 MHz)
compound 9c
Cl
NH2
CH3
f1 (ppm)

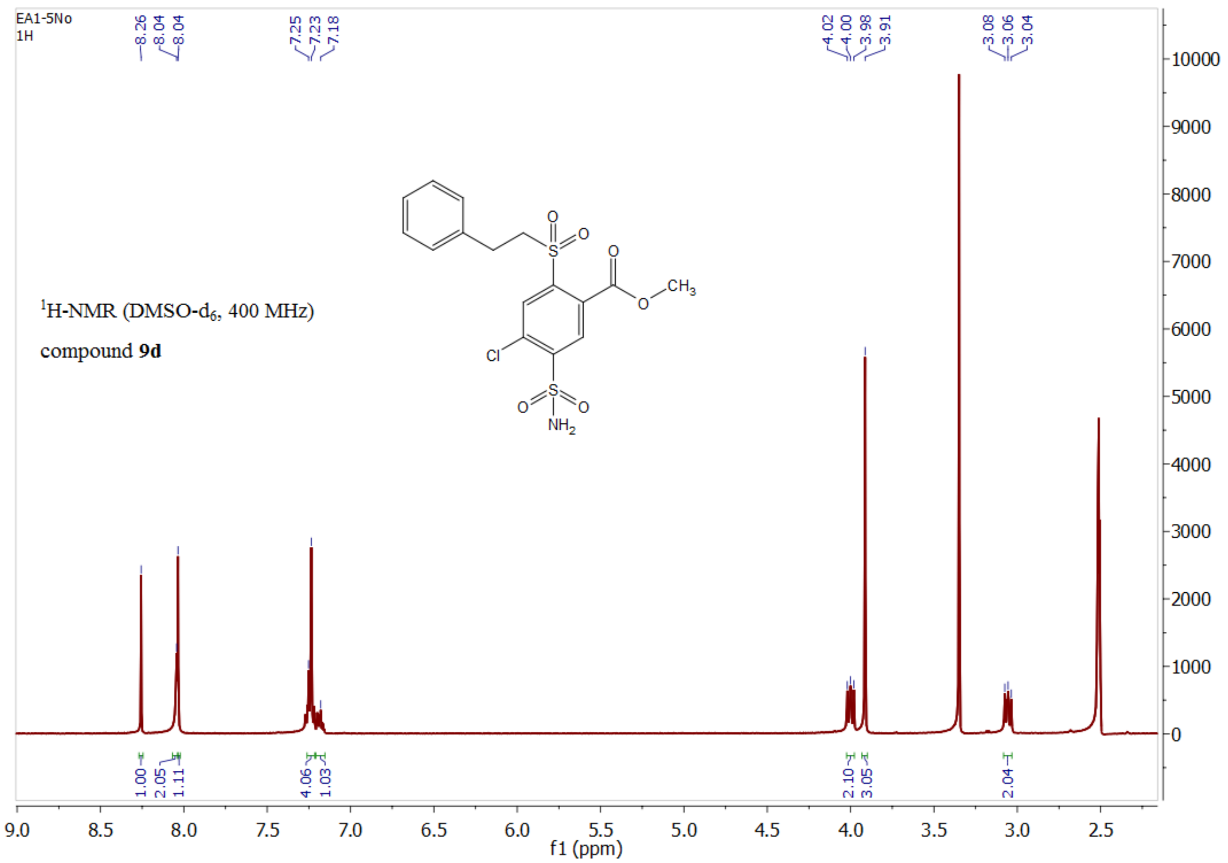
EA1-5No
1H
^{1}H-NMR (DMSO-d$_6$, 400 MHz)
compound **9d**
f1 (ppm)

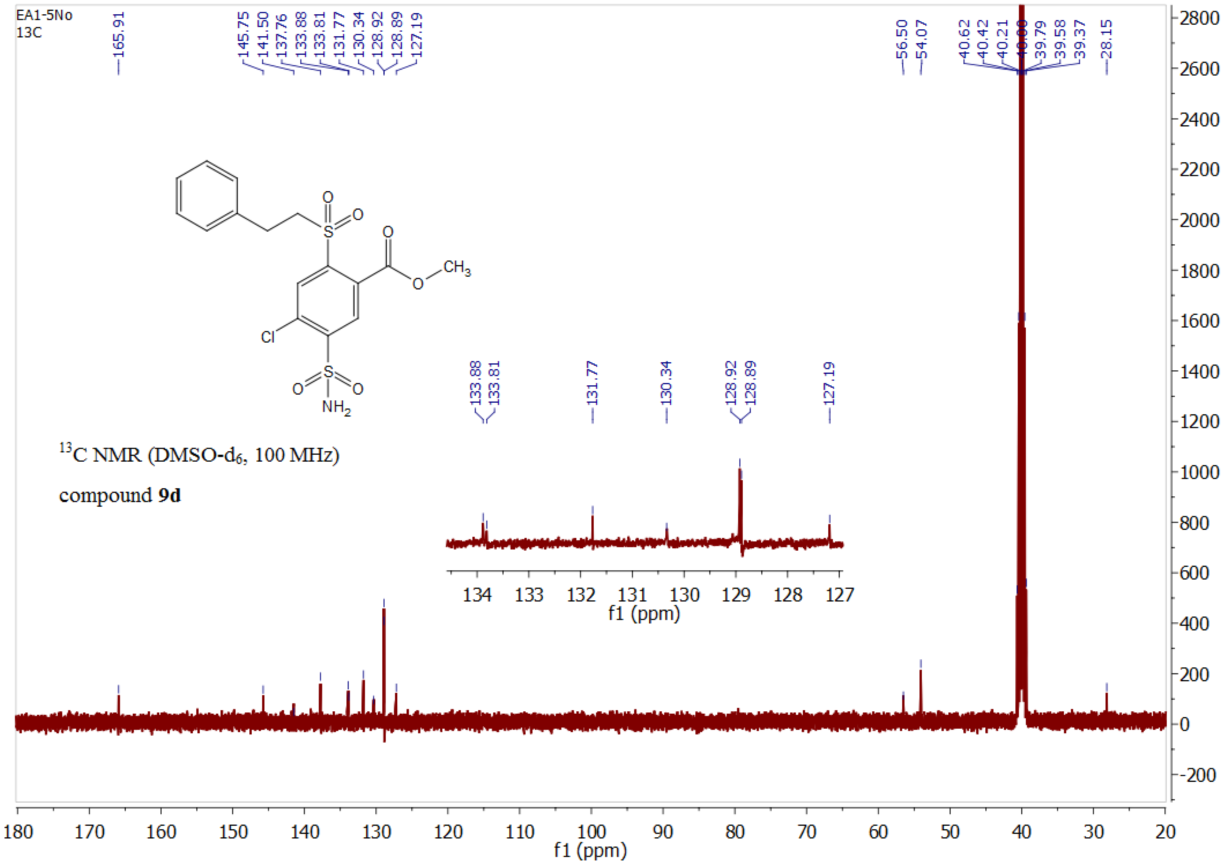
EA1-5No
13C
^{13}C NMR (DMSO-d$_6$, 100 MHz)
compound **9d**
f1 (ppm)

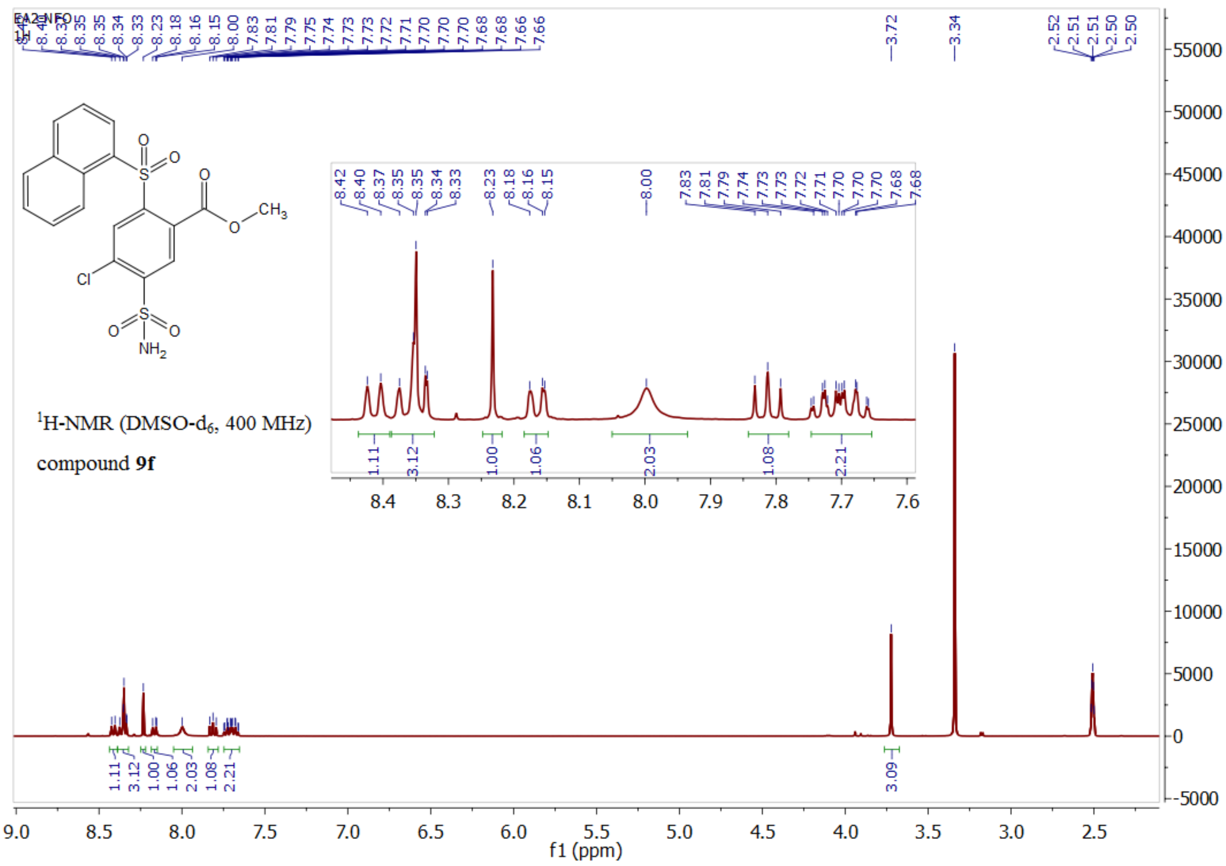
EA2-NFO
1H
¹H-NMR (DMSO-d6, 400 MHz)
compound 9f
Cl
NH2
CH3
f1 (ppm)
8.42 8.40 8.37 8.35 8.35 8.34 8.33 8.23 8.18 8.16 8.15 8.00 7.83 7.81 7.79 7.75 7.74 7.73 7.73 7.72 7.71 7.70 7.70 7.70 7.68 7.68 7.66 7.66
3.72
3.34
2.52 2.51 2.51 2.50 2.50
1.11 3.12 1.00 1.06 2.03 1.08 2.21 3.09

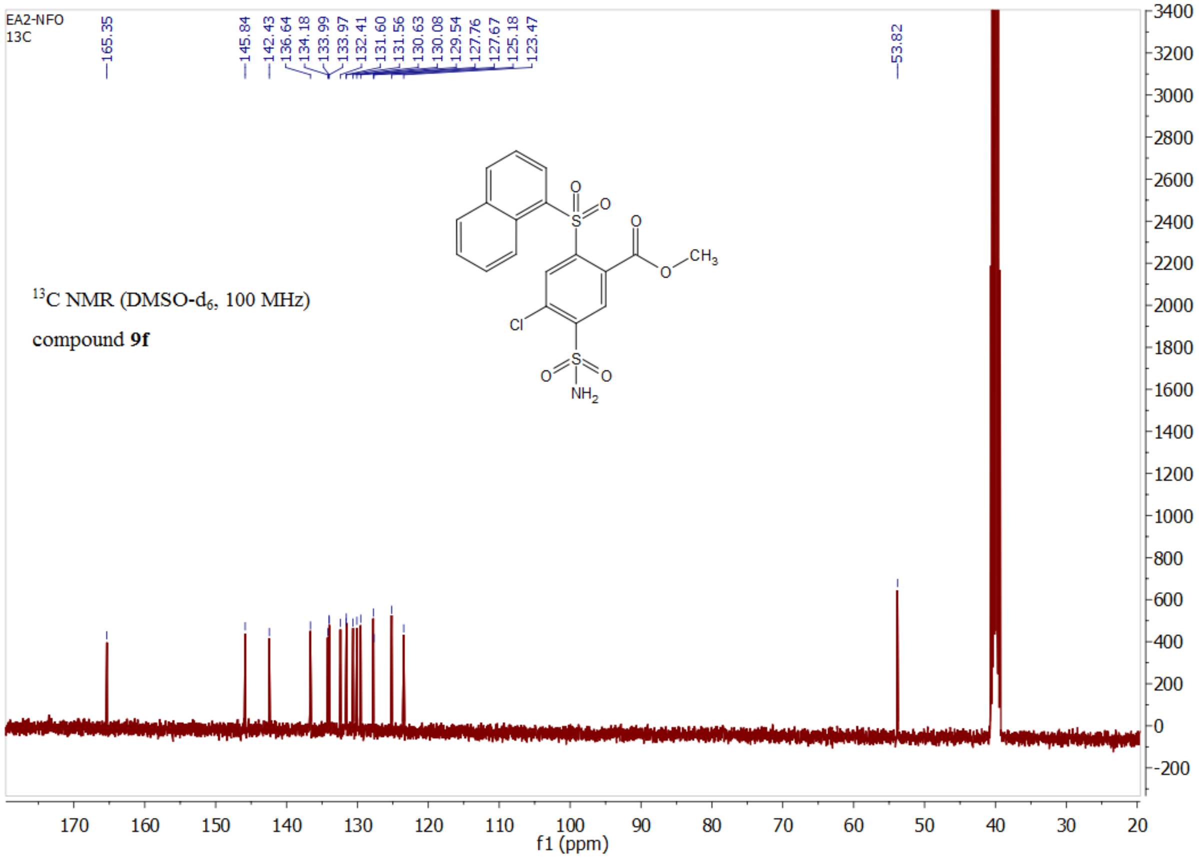
EA2-NFO
13C
¹³C NMR (DMSO-d6, 100 MHz)
compound 9f
Cl
NH2
CH3
f1 (ppm)
165.35
145.84 142.43 136.64 134.18 133.99 133.97 132.41 131.60 131.56 130.63 130.08 129.54 127.76 127.67 125.18 123.47
53.82

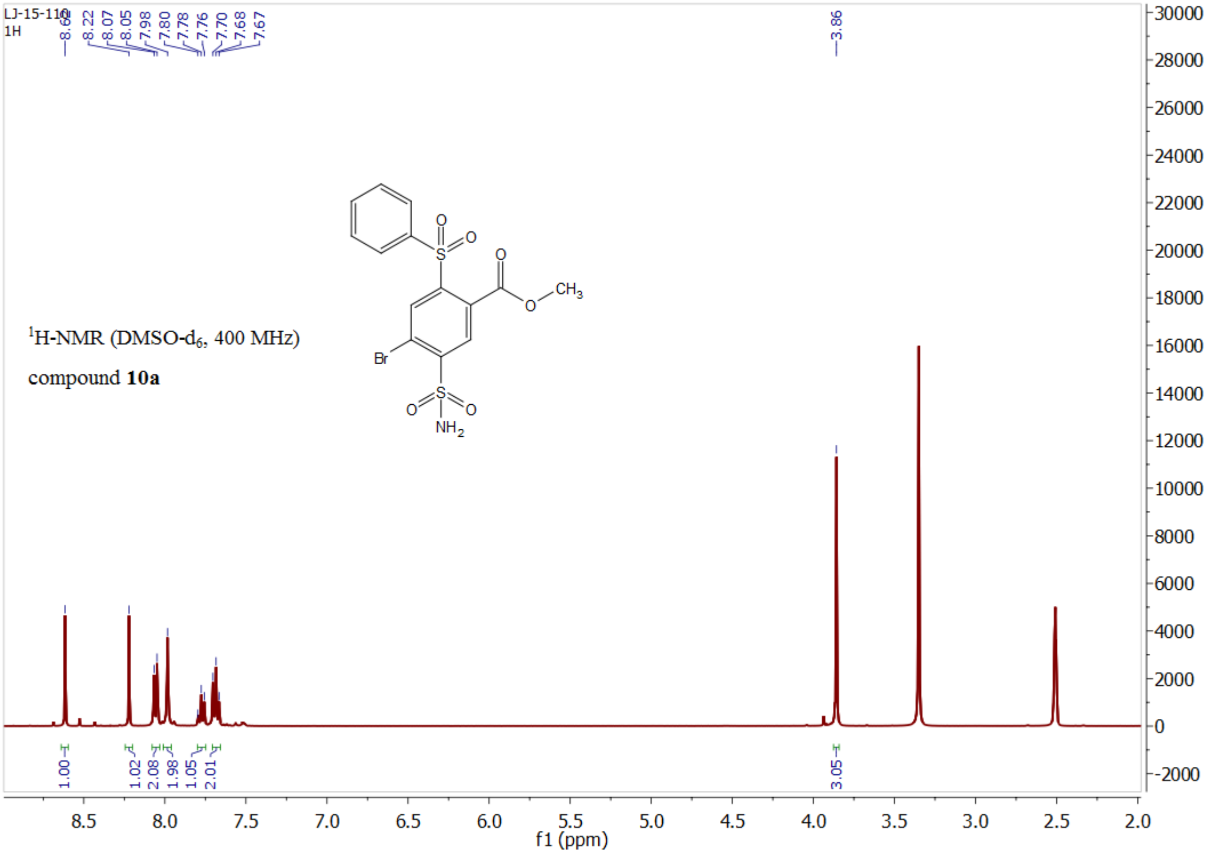
LJ-15-110
1H
8.62
8.22
8.07
8.05
7.98
7.80
7.78
7.76
7.70
7.68
7.67
3.86
1H-NMR (DMSO-d6, 400 MHz)
compound 10a
Br
O
CH3
NH2
S
1.00
1.02
2.08
1.98
1.05
2.01
3.05
f1 (ppm)

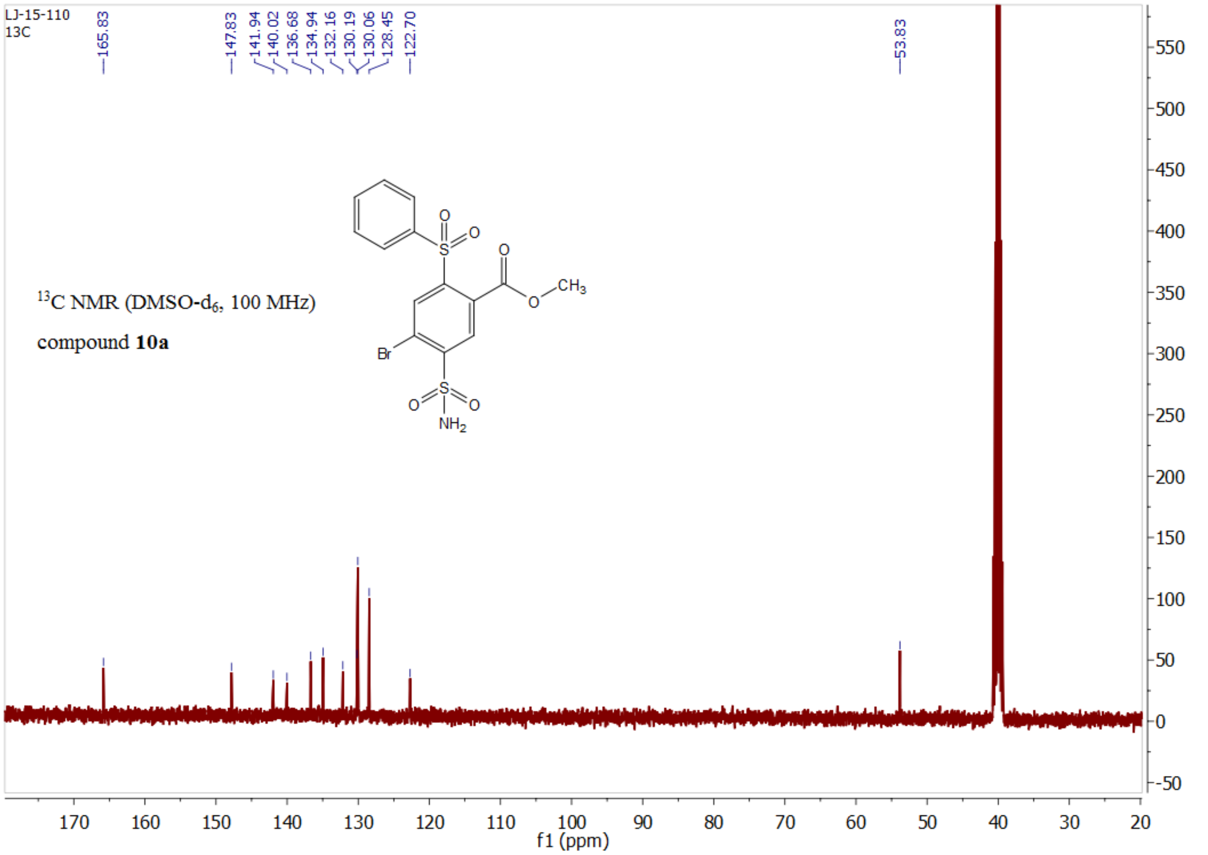
LJ-15-110
13C
165.83
147.83
141.94
140.02
136.68
134.94
132.16
130.19
130.06
128.45
122.70
53.83
13C NMR (DMSO-d6, 100 MHz)
compound 10a
Br
O
CH3
NH2
S
f1 (ppm)

References

Goldberg RN, Kishore N, Lennen RM (2002) Thermodynamic Quantities for the Ionization Reactions of Buffers. J Phys Chem Ref Data 31:231–370. https://doi.org/10.1063/1.1416902

Linkuvienė V, Zubrienė A, Manakova E, Petrauskas V, Baranauskienė L, Zakšauskas A, Smirnov A, Gražulis S, Ladbury JE, Matulis D (2018) Thermodynamic, kinetic, and structural parameterization of human carbonic anhydrase interactions toward enhanced inhibitor design. Q Rev Biophys 51:1–48. https://doi.org/10.1017/S0033583518000082

Zakšauskas A, Čapkauskaitė E, Jezepčikas L, Linkuvienė V, Paketurytė V, Smirnov A, Leitans J, Kazaks A, Dvinskis E, Manakova E, Gražulis S, Tars K, Matulis D (2020) Halogenated and di-substituted benzenesulfonamides as selective inhibitors of carbonic anhydrase isoforms. Eur J Med Chem 185:111825. https://doi.org/10.1016/j.ejmech.2019.111825

Zubrienė A, Matulis D (2019) Observed Versus Intrinsic Thermodynamics of Inhibitor Binding to Carbonic Anhydrases. In: Matulis D (ed) Carbonic Anhydrase as Drug Target: Thermodynamics and Structure of Inhibitor Binding. Springer International Publishing, Cham, pp 107–123
